# A microenvironment-determined risk continuum refines subtyping in meningioma and reveals determinants of machine learning-based tumor classification

**DOI:** 10.1038/s41588-025-02475-w

**Published:** 2026-02-09

**Authors:** Sybren L. N. Maas, Yiheng Tang, Eric Stutheit-Zhao, Ramin Rahmanzade, Christina Blume, Thomas Hielscher, Ferdinand Zettl, Salvatore Benfatto, Domenico Calafato, Martin Sill, Jasim Kada Benotmane, Yahaya A. Yabo, Felix Behling, Abigail Suwala, Helin Kardo, Michael Ritter, Matthieu Peyre, Roman Sankowski, Konstantin Okonechnikov, Philipp Sievers, Areeba Patel, David Reuss, Mirco J. Friedrich, Damian Stichel, Daniel Schrimpf, Thierry P. P. Van den Bosch, Katja Beck, Hans-Georg Wirsching, Gerhard Jungwirth, C. Oliver Hanemann, Katrin Lamszus, Nima Etminan, Andreas Unterberg, Christian Mawrin, Marc Remke, Olivier Ayrault, Peter Lichter, Guido Reifenberger, Michael Platten, Tim Kacprowski, Markus List, Josch K. Pauling, Jan Baumbach, Till Milde, Rachel Grossmann, Zvi Ram, Miriam Ratliff, Jan-Philipp Mallm, Marian C. Neidert, Eelke M. Bos, Marco Prinz, Michael Weller, Till Acker, Felix J. Hartmann, Matthias Preusser, Ghazaleh Tabatabai, Christel Herold-Mende, Sandro M. Krieg, David T. W. Jones, Stefan M. Pfister, Wolfgang Wick, Michel Kalamarides, Andreas von Deimling, Dieter Henrik Heiland, Volker Hovestadt, Moritz Gerstung, Matthias Schlesner, The German “Aggressive Meningiomas” Consortium (KAM), Katrin Lamszus, Christian Mawrin, Marc Remke, Guido Reifenberger, Christel Herold-Mende, Wolfgang Wick, Matthias Schlesner, Felix Sahm, Felix Sahm

**Affiliations:** 1 Department of Pathology, Leiden University Medical Center, Leiden, The Netherlands; 2 Department of Pathology, Brain Tumor Center, Erasmus MC Cancer Institute, University Medical Center Rotterdam, Rotterdam, The Netherlands; 3 Department of Neuropathology, University Hospital Heidelberg, Heidelberg, Germany; 4 Clinical Cooperation Unit Neuropathology, German Cancer Research Centre (DKFZ), German Consortium for Translational Cancer Research (DKTK), Heidelberg, Germany; 5 Division of AI in Oncology, German Cancer Research Centre (DKFZ), Heidelberg, Germany; 6 Department of Radiation Oncology, University of Toronto, Toronto, ON Canada; 7 Hopp Children’s Cancer Center Heidelberg (KiTZ), Heidelberg, Germany; 8 Institute of Pathology, Ludwig Maximilians University Hospital Munich, Munich, Germany; 9 Department of Biostatistics, German Cancer Research Center (DKFZ), Heidelberg, Germany; 10 Department of Pediatric Oncology, Dana-Farber Cancer Institute, Boston, MA USA; 11 Division of Hematology/Oncology, Boston Children’s Hospital, Boston, MA USA; 12 Broad Institute of MIT and Harvard, Cambridge, MA USA; 13 German Cancer Research Center (DKFZ), Heidelberg, Germany; 14 Systems Immunology & Single-Cell Biology, Heidelberg, Germany; 15 Division of Pediatric Neurooncology, German Cancer Consortium (DKTK) and German Cancer Research Center (DKFZ), Heidelberg, Germany; 16 Translational Neurosurgery, Neurosurgical Clinic, Friedrich-Alexander Universität Erlangen-Nürnberg, Erlangen, Germany; 17 Department of Neurosurgery, University Hospital Augsburg, Augsburg, Germany; 18 Translational oncological Neurosurgery, Faculty of Medicine, Augsburg University, Augsburg, Germany; 19 Sorbonne Université - Department of Neurosurgery, Groupe Hospitalier Pitié-Salpêtrière, APHP, Paris, France; 20 Neurovascular interfaces in Brain Tumors and vascular malformations - CRICM INSERM U1127 CNRS UMR 7225 - Brain Institute, Hôpital de la Pitié-Salpêtrière, Paris, France; 21 Institute of Neuropathology, Faculty of Medicine, University of Freiburg, Freiburg, Germany; 22 German Cancer Research Center (DKFZ), JRG Hematology and Immune Engineering, Heidelberg, Germany; 23 German Cancer Consortium (DKTK), DKFZ, Core Center Heidelberg, Heidelberg, Germany; 24 Heidelberg Institute for Stem Cell Technology and Experimental Medicine (HI-STEM gGmbH), Heidelberg, Germany; 25 Department of Hematology, Oncology and Rheumatology, Heidelberg University Hospital, Heidelberg, Germany; 26 Division of Translational Medical Oncology, German Cancer Research Center (DKFZ), Heidelberg, Germany; 27 National Center for Tumor Diseases (NCT), NCT Heidelberg, a partnership between DKFZ and Heidelberg University Hospital, Heidelberg, Germany; 28 Department of Neurology, University Hospital and University of Zurich, Zurich, Switzerland; 29 Department of Neurology, Kantonsspital Winterthur, Winterthur, Switzerland; 30 Department of Neurosurgery, University Hospital Heidelberg, Heidelberg, Germany; 31 Faculty of Health, Peninsula Medical School, University of Plymouth, Plymouth, UK; 32 Department of Neurosurgery, University Medical Center Hamburg-Eppendorf, Hamburg, Germany; 33 Department of Neurosurgery, University Hospital Mannheim, University of Heidelberg, Mannheim, Germany; 34 Department of Neuropathology, University Hospital Magdeburg, Magdeburg, Germany; 35 Department of Pediatric Hematology and Oncology, University Medical Center of Saarland, Saarland University, Homburg/Saar, Germany; 36 Institut Curie, PSL Research University, INSERM U1330/CNRS EMR 8001. Children’s Oncology Research Unit (CONCERT), Paris, France; 37 Université Paris Sud, Université Paris-Saclay, INSERM U1330/CNRS EMR 8001. Children’s Oncology Research Unit (CONCERT), Paris, France; 38 Division of Molecular Genetics, German Cancer Research Center (DKFZ), and NCT Heidelberg, Heidelberg, Germany; 39 Institute of Neuropathology, Heinrich Heine University, Medical Faculty, and University Hospital Düsseldorf, Düsseldorf, Germany; 40 Department of Neurology, University Hospital and Medical Faculty Mannheim, Mannheim, Germany; 41 Chair of Experimental Bioinformatics, TUM School of Life Sciences, Technical University of Munich, Freising, Germany; 42 Institute of Data Science in Biomedicine, Technische Universität Braunschweig, Braunschweig, Germany; 43 Braunschweig Integrated Centre of Systems Biology (BRICS), TU Braunschweig, Braunschweig, Germany; 44 Data Science in Systems Biology, School of Life Sciences, Technical University of Munich, Freising, Germany; 45 LipiTUM, Chair of Experimental Bioinformatics, TUM School of Life Sciences, Technical University of Munich, Freising, Germany; 46 CIOBio, Institute for Clinical Chemistry and Laboratory Medicine, University Hospital and Faculty of Medicine Carl Gustav Carus, Dresden University of Technology, Dresden, Germany; 47 Institute for Computational Systems Biology, University of Hamburg, Hamburg, Germany; 48 Computational Biomedicine Lab, Department of Mathematics and Computer Science, University of Southern Denmark, Odense, Denmark; 49 University Hospital Jena, Department of Pediatrics and Adolescent Medicine, Friedrich Schiller University Jena, Jena, Germany; 50 Comprehensive Cancer Center Central Germany (CCCG), Jena, Germany; 51 Clinical Cooperation Unit Pediatric Oncology, German Cancer Research Center Heidelberg (DKFZ), Heidelberg, Germany; 52 Department of Neurosurgery, Rambam Health Care Campus, Rappaport Faculty of Medicine, Technion, Haifa, Israel; 53 Department of Neurosurgery, Tel Aviv Medical Center, Tel Aviv, Israel, and School of Medicine, Tel Aviv University, Tel Aviv, Israel; 54 Single-cell Open Lab, German Cancer Research Centre (DKFZ), Heidelberg, Germany; 55 Department of Neurosurgery and Clinical Neuroscience Center, University Hospital and University of Zurich, Zurich, Switzerland; 56 Department of Neurosurgery, HOCH Health Ostschweiz, Kantonsspital St. Gallen, St. Gallen, Switzerland; 57 Department of Neurosurgery, Brain Tumor Center, Erasmus MC Cancer Institute, University Medical Center Rotterdam, Rotterdam, The Netherlands; 58 Signalling Research Centres BIOSS and CIBSS, University of Freiburg, Freiburg, Germany; 59 Institute of Neuropathology, Justus-Liebig-University Gießen, Giessen, Germany; 60 Division of Oncology, Department of Medicine I, Medical University of Vienna, Vienna, Austria; 61 Department of Neurology, University Hospital Tübingen, Eberhard-Karls-University Tübingen, Tübingen, Germany; 62 Pediatric Glioma Research Group, German Cancer Research Center (DKFZ), Heidelberg, Germany; 63 Department of Pediatric Hematology and Oncology, Heidelberg University Hospital, Heidelberg, Germany; 64 Department of Neurology and Neurooncology Program, National Center for Tumor Diseases, Heidelberg University & German Cancer Research Center (DKFZ), Heidelberg, Germany; 65 Department of Neurosurgery, Medical Centre-University of Freiburg, Freiburg, Germany; 66 Microenvironment and Immunology Research Laboratory, Friedrich-Alexander Universität Nürnberg-Erlangen, Erlangen, Germany; 67 Department of Neurosurgery, University Hospital Erlangen, Friedrich-Alexander University Erlangen Nuremberg, Erlangen, Germany; 68 Department of Neurological Surgery, Northwestern University Feinberg School of Medicine, and Malnati Brain Tumor Institute, Northwestern Medicine, Chicago, IL USA; 69 Faculty of Medicine, Freiburg University, Freiburg, Germany; 70 Faculty of Mathematics and Computer Science, Heidelberg University, Heidelberg, Germany; 71 Robert Bosch Center for Tumor Diseases, Stuttgart, Germany; 72 Medical Faculty, Eberhard-Karls-University, Tübingen, Germany; 73 University Hospital Tübingen, Tübingen, Germany; 74 Biomedical Informatics, Data Mining and Data Analytics, Faculty of Applied Computer Science and Medical Faculty, University of Augsburg, Augsburg, Germany

**Keywords:** Translational research, Cancer microenvironment, CNS cancer

## Abstract

Classification of tumors in neuro-oncology today relies on molecular patterns (mostly DNA methylation) and their machine learning-supported interpretation. Understanding the process of algorithmic interpretation is essential for safe application in clinical routine. This is paradigmatically true for the most common primary intracranial tumor in adults, meningioma. Here, by applying multiomic profiling and multiple lines of orthogonal computational evaluation in multiple independent datasets, we found that not only tumor cell characteristics but also incremental changes in the tumor microenvironment (TME) have impact on epigenetic meningioma classification and clinical outcome. Besides revealing the decisive role of non-neoplastic cells in the CNS methylation classifier, this challenges the model of distinct meningioma subgroups toward a TME-determined risk continuum. This refines current controversies in molecular meningioma subtyping. In addition, we apply these learnings to devise and validate a simple diagnostic approach for increased clinical prediction accuracy based on immunohistochemistry, which is also applicable in resource-limited settings.

## Abstract (web summary)

This paper uses multiomics to profile a large cohort of meningioma samples to highlight the role of the tumor microenvironment in driving the epigenetic changes underpinning tumor classification and prediction of outcome.

## Main

**Fig. 1:**
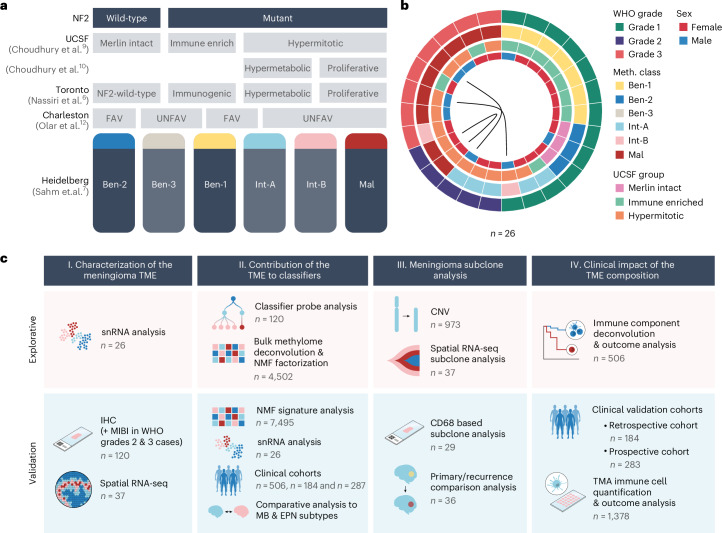
Meningioma molecular classification models and snRNA cohort overview. **a**, In general, meningioma tumors can be divided based on the *NF2* status into *NF2*-wild-type and *NF2*-mutant meningioma. Multiple molecular subclassification models exist covering the spectrum of meningioma tumors with different granularity. **b**, From 26 meningiomas of different WHO grades and methylation classes or groups, single-nucleus RNA (snRNA) data were generated for further analyses. Primary/recurrent pairs are connected through a line. **c**, The different individual experiments presented in the article construct four different parts, each consisting of exploratory and validation experiments. Favorable (FAV), unfavorable (UNFAV), tumor microenvironment (TME), snRNA sequencing (snRNA-seq), immunohistochemistry (IHC), multiplexed ion beam imaging (MIBI), WHO, non-negative matrix factorization (NMF), medulloblastoma (MB), ependymoma (EPN), copy-number variation (CNV) and tissue microarray (TMA).

Machine learning (ML) approaches to molecular data, foremost DNA methylation profiles, have transformed diagnostics in neuro-oncology^1^, including meningiomas^2^. In 2021, the homozygous loss of *CDKN2A/B* and/or a *TERT*-promotor mutation were added as criteria for World Health Organization (WHO) grade 3 (ref. ^3^). Beyond risk prediction based on single markers, several molecular classification systems incorporating genome-wide information have been proposed^4–12^, mostly based on DNA methylation^2^. Only three subtypes overlap in the various schemes: an *NF2*-wild-type group, an *NF2-*mutant low-grade group (according to two publications with immune cell enrichment^6,9^) and an *NF2-*mutant high-grade group (Fig. 1a)^13^. For example, we have proposed and validated a classification encompassing these three “consensus” classes and in total six subclasses^7^. Other proposed systems contain only two to four subtypes, with necessarily incomplete overlap in morphological, molecular and, importantly, clinical characteristics. Understanding the background of the divergent signal for number and type of relevant subgroups in DNA methylation profiling could provide the basis for a global consensus in meningioma.

## Results

We first investigated meningioma cellular composition by generating single-nucleus RNA sequencing (snRNA-seq) of 26 cases from all WHO grades and all three consensus groups: *NF2* wild-type, *NF2*-mutant low-grade and *NF2*-mutant high-grade (Fig. 1a–c). Herein, we applied the terminology that reflects the clinical behavior and is used in the Heidelberg Brain Tumor Classifier. Accordingly, *NF2*-mutant low-grade is referred to as methylation class (MC) ben-1, *NF2* wild-type as MC ben-2 and *NF2*-mutant high-grade as MC mal^5,7^. We also included cases from the MCs that are exclusive to our six-tiered high-granularity grading scheme: MC int-A/B (*NF2*-mutant, clinically between ben-1 and mal). Samples and their characteristics are depicted in Fig. 1b.

### Cellular composition of the tumor-microenvironment

**Fig. 2:**
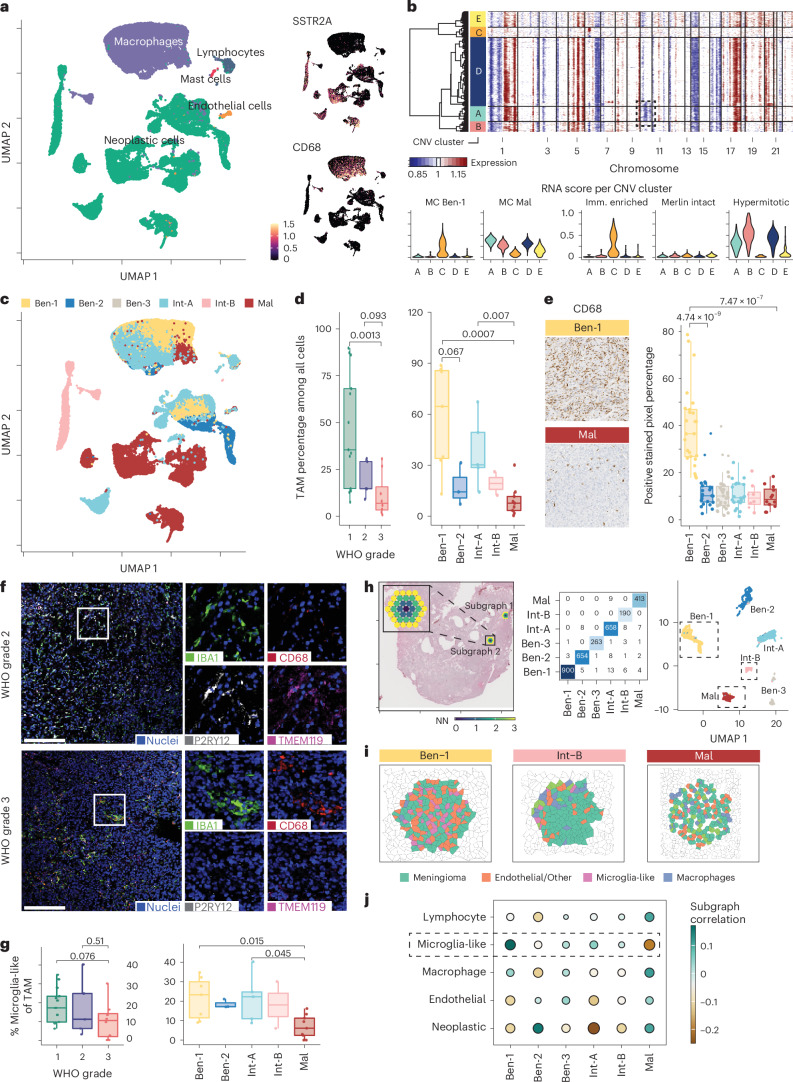
Tumor-associated macrophages are the largest subset of non-neoplastic cells that differ in number and phenotype by meningioma grade and MC. **a**, UMAP of 44,266 meningioma scRNA nuclei identified multiple cellular clusters of different origins. Neoplastic cells, determined based on the presence of CNVs and/or *SSTR2A* expression, are the largest set of cells, followed by tumor-associated macrophages (TAMs), as determined by *CD68* expression. **b**, CNV analysis of all neoplastic cells of a representative case identified five CNV clusters, for example based on the presence of an additional loss of chromosome 10 in a subset of cells (top panel). MC or methylation group (MG) prediction for cells within a CNV cluster based on RNA expression levels as shown on the *y*-axis (bottom panel). **c**, Within the non-neoplastic clusters, for example TAMs, cells from different MCs cluster together, indicating differences in TAM gene expression between MCs. **d**, Number of TAMs, as determined by snRNA expression levels, decreased with an increase in meningioma aggressiveness as determined by WHO grade or MC. Percentage of TAMs relative to all cells within each sample is displayed. Significance levels were tested with two-sided Mann-Whitney *U* tests (snRNA cohort composition in Fig. 1b). **e**, At the protein level, MC ben-1 *NF2-*mutant benign meningioma likewise contain the highest level of CD68-positive TAMs, as quantified by the CD68-positive surface area per case. Furthermore, the dotted CD68 staining pattern in low-risk meningioma indicates an enrichment of microglia-like cells in those cases. Significance levels were tested with two-sided ANOVA tests (MC ben-1 *n* = 25, ben-2 *n* = 23, ben-3 *n* = 31, int-A *n* = 21, int-B *n* = 8 and mal *n* = 12). **f**, A multiplex antibody panel performed on meningioma from six different patients dividing TAMs into microglia-like cells (CD68^low^, IBA1, TMEM119 and P2RY12 positive) versus infiltrating macrophages (CD68^high^, IBA1 positive, TMEM119 and P2RY12 negative) indicates that lower grade (grade 2) meningioma has a mixed population of TAMs, whereas the high number of microglia-like in lower grade meningioma is strongly reduced in aggressive grade 3 meningioma. Scale bar indicates 200μm. **g**, The difference in TAM composition between low-risk and high-risk meningioma was confirmed at the snRNA level when the percentage of microglia-like cells was calculated from the overarching TAM population for each individual sample. Significance levels were tested with two-sided Mann-Whitney *U* tests (snRNA cohort composition in Fig. 1b). **h**, Overlay of spatial RNA-seq subgraph examples on an hematoxylin and eosin (H&E) staining of an analyzed meningioma sample (left panel, inset: example subgraph built of individually connected “3-hop” nodes). Confusion matrix of the predicted (from spatial transcriptomics) versus ground truth (MC from epigenetic profiling) (middle panel). UMAP dimensional reduction of the latent space representation of the GAN, indicating clear overlay of the identified TMEs defined by spatial RNA-seq and the MCs these subgraphs were derived from (right panel). **i**, Visual representation of the single-cell distribution within three example subgraphs. Cell deconvolution was performed by Cell2location based on the reference single-cell sequencing data. **j**, Correlation analysis of the subclass logit (logarithm of the odds) scores (*x*-axis) and the cell-type abundance (*y*-axis) of all subgraphs. Diameter and color both represent the correlation. Boxplots represent the upper and lower quartiles, whereas whiskers reach to the most outlying samples that are within 1.5 interquartile range (IQR) of the quartiles. Median values are labeled as horizontal lines in the boxes.

**Extended Data Fig. 1:**
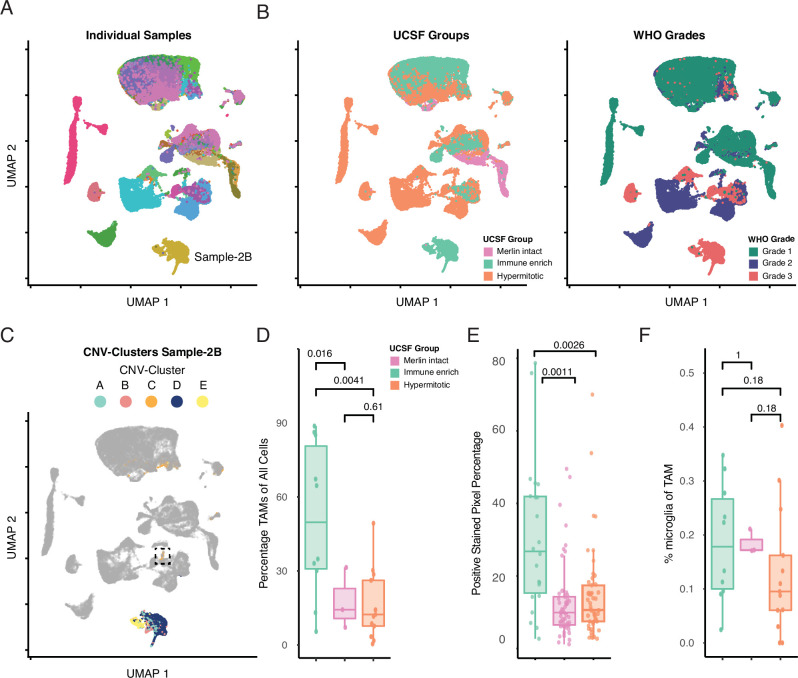
snRNA meningioma sample visualization and tumour-associated macrophage quantification. Uniform Manifold Approximation and Projection (UMAP) visualizations of the individual samples of the 26 cases from which nuclei were isolated (**A**), the different methylation groups (**B**, left panel) or the different WHO grades (**B**, right panel). In one illustrative case, within the neoplastic cells, five CNV clusters were identified. Cells from CNV cluster C are located away from the other neoplastic cells (**C**). Distribution of the number of combined tumour-associated macrophages (TAMs) as calculated by the TAM percentage for each individual sample (**D**), the percentage of surface area positively stained for CD68 for each individual sample (**E**) and percentage of microglia-like cells within the overarching TAM population for each sample (**F**) for the methylation groups. Boxplots represent the upper and lower quartiles, while whiskers reach to the most outlying samples that are within 1.5 interquartile ranges (IQR) from the quartiles. Median values are labeled as horizontal lines in the boxes. For subfigures **D** and **F**, Immune enriched *n* = 10, Merlin intact *n* = 3 and Hypermitotic *n* = 13. For subfigure **E**, Immune enriched *n* = 22, Merlin intact *n* = 49 and Hypermitotic *n* = 49.

In transcript data from 44,266 single nuclei, the largest cluster consisted of meningioma tumor cells as defined by *SSTR2A* expression and presence of typical copy-number variations (CNVs) such as chromosome 22q, 1p, 6p/q, 14p/q and 18p/q loss (Fig. 2a, b)^4,14^. Tumor cells from individual samples, WHO grade and MC largely clustered together (Fig. 2c and Extended Data Fig. 1a, b). Single-cell CNVs within a given sample were highly diverse in some cases. For example, one sample contained a majority of tumor cells harboring losses in chromosomes 1p, 6q, 14q, 16p/q and 22q and only a subclone showing additional 10q loss (Fig. 2b)^4,15,16^. With hierarchical clustering, five CNV clusters (clusters A–E) were identified. Although CNV cluster A contains the additional loss of chromosome 10q, cells from this cluster still localize closely with other neoplastic cells according to uniform manifold approximation and projection (UMAP) (Extended Data Fig. 1c). CNV cluster C cells harbor a limited number of CNVs and were identified as both stromal and neoplastic cells. In contrast to CNV cluster A, CNV cluster C cells cluster separately in the UMAP (Extended Data Fig. 1c). When predicting the MC for individual cells based on RNA expression, CNV cluster C cells obtain the highest scores for MC ben-1 or methylation group (MG, as defined by the UCSF classification scheme) immune enriched^9^. In line with matching bulk methylation analysis, other CNV clusters harboring risk-associated CNVs obtain the highest RNA score for MC mal, with CNV cluster A having the most pronounced score (Fig. 2b). Even though the UCSF classification methodology accounts for CNVs, CNV clusters A, B and D all obtained high, yet slightly different, RNA scores for the hypermitotic MG also indicating high risk. Together, these data indicate that meningiomas contain subclones and that MC/MG RNA expression association can vary among subclones.

The second largest group contained tumor-associated myeloid-derived cells (referred to here as tumor-associated macrophages, TAMs, for simplicity) expressing *CD68* (Fig. 2a). The TAM cluster was enriched for WHO grade 1 cases. Within TAMs, cells from higher WHO grades and/or more aggressive MC in general clustered together, suggesting that TAMs in high-risk meningioma have distinct RNA expression (Fig. 2c and Extended Data Fig. 1b). Stratifying TAM abundance across WHO grade and MCs revealed that TAM prevalence decreases with increased risk (Fig. 2d and Extended Data Fig. 1d for MGs). This is contrary to diffuse gliomas, where TAM numbers increase with WHO grade^17^.

As RNA expression analysis (especially single-cell/snRNA experiments) can introduce bias, including myeloid-derived cell enrichment^18,19^, we orthogonally validated these findings. CD68 immunohistochemistry on 120 different meningiomas confirmed the high number of TAMs in MC ben-1 (Fig. 2e and Extended Data Fig. 1e for MGs). Of note, CD68 expression is shared between both infiltrating myeloid-derived monocytes/macrophages and brain-resident microglia-like cells^20^. By examination of the CD68 staining, we noted that the characteristic dotted/striped pattern of microglia-like cells and other homeostatic macrophages was more abundant in MC ben-1, whereas larger cells with more cytoplasm (typical for foamy myeloid-derived infiltrative macrophages) were largely confined to more aggressive meningiomas. As yet another validation, we performed multiplexed ion beam imaging (MIBI) on separate WHO grade 2 and 3 meningiomas (Fig. 2f). In grade 2 meningioma, cells in which CD68 was expressed (albeit at low levels) also showed the microglia markers P2RY12 and TMEM119 (refs. ^21–24^). In grade 3 meningioma, however, TAMs demonstrated strong CD68 expression but no microglial markers, identifying the cells as myeloid-derived infiltrative monocytes/macrophages^20,21^. Hence, TAM composition differs between meningioma stages. For further validation, snRNA TAMs were in silico sorted for either brain-resident microglia-like cells (*TMEM119*, *P2RY13*, *P2RY12*, *GPR34* and *SLC2A5*) or myeloid-derived monocyte/macrophage-like cells (*C3*, *F10*, *EMILIN2*, *F5*, *GDA*, *MKI67*, *SELL* and *HP*)^21,25–27^. Here, we detected a decrease in microglia-like cell percentage in WHO grade 3 compared to grade 1, although this decrease was not statistically significant. This difference was statistically significant when comparing MCs, particularly mal to ben-1 (Fig. 2g and Extended Data Fig. 1f for MGs). This discrepancy likely results from WHO grade 1 *NF2-*wild-type (MC ben-2) cases that do not harbor similar high fractions of microglia-like cells.

**Extended Data Fig. 2:**
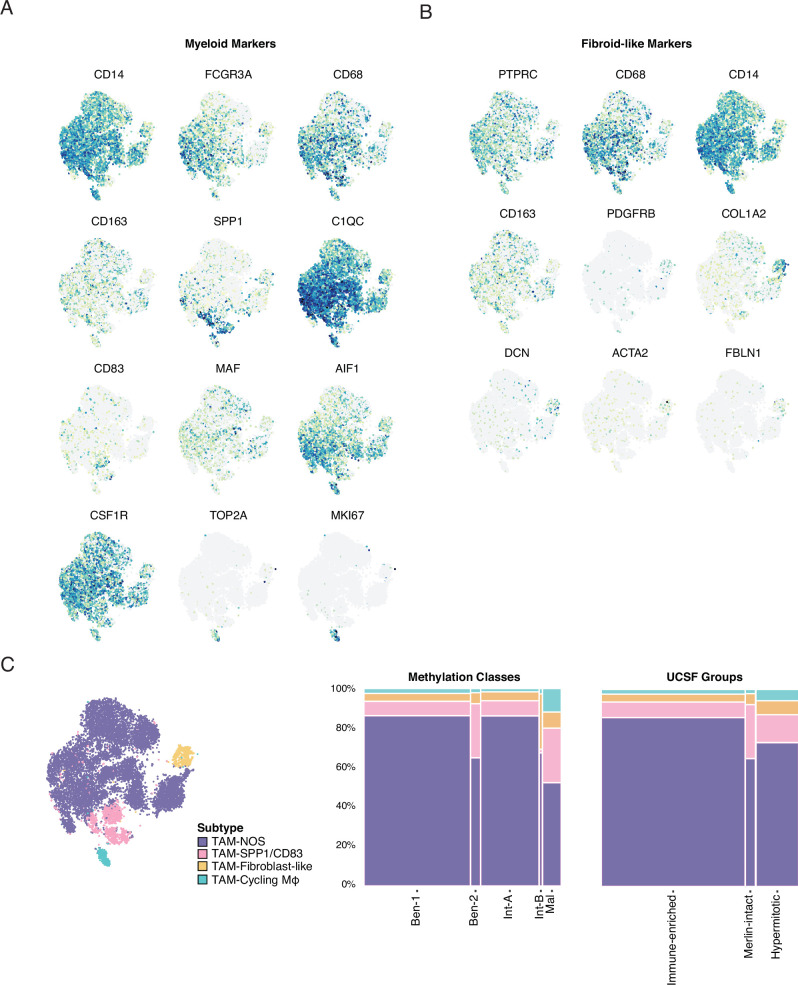
snRNA meningioma tumour-associated macrophage analyses. Further investigation of the tumour-associated macrophages (TAMs) in a separate clustering analysis including different myeloid (**A**) and fibroid-like (**B**). Four separate clusters were identified (**C**, left panel). Subgroup distribution and relative quantification of the myeloid clusters for the methylation classes and methylation groups (**C**, right panel).

**Extended Data Fig. 3:**
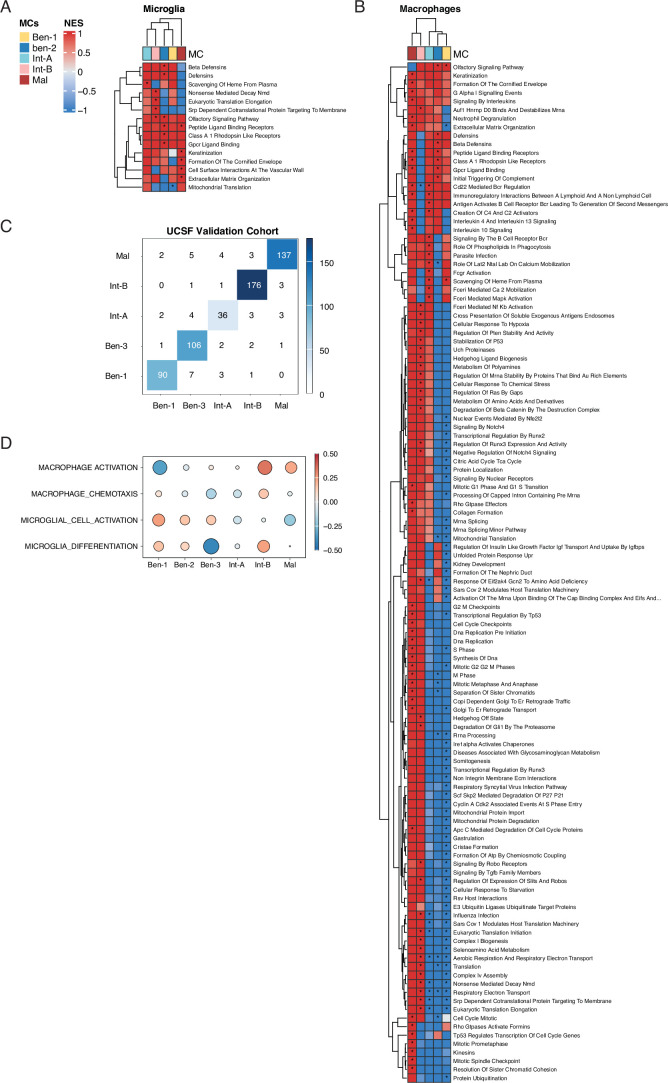
Gene set enrichment analyses of meningioma microglia-like cells and macrophages. Gene set enrichment analysis (GSEA) comparing snRNA differential expression between microglia-like (**A**) and macrophage-like cells (**B**) from different methylation classes. Macrophages from MC mal show increased expression of proliferation and mitotic pathways as well as the known tumour-supportive macrophage activation states of the interleukin-10 (IL-10) and interleukin-/13 (IL-4/IL-13) pathways. Confusion matrix of the predicted (from spatial transcriptomics) vs ground truth (MC from epigenetic profiling) for the validation cohort (**C**). Correlation plot from spatial analysis in the discovery set indicating microglia differentiation and activation in MC ben-1 and macrophage activation in MC mal. Diameter and color both represent the correlation (**D**). Normalized enrichment score (NES). Astrix (*) indicates a false discovery rate (FDR) adjusted p-value < 0.05.

Based on gene expression patterns, MC mal meningiomas were enriched with cycling macrophages as defined by *TOP2A* and *MKI67* expression and by *SPP1*/*CD83*-positive TAMs (Extended Data Fig. 2a–c). This aligns with increased chemoresistance and inferior outcome in different epithelial cancers and with tumor progression in diffuse gliomas that harbor high amounts of SPP1-positive macrophages^28–30^. Next, we investigated TAM activation by gene set enrichment analysis (GSEA). Although gene set expression between low-risk MC ben-1 and aggressive MC mal microglia-like cells was minimal, MC mal macrophages showed significant enrichment of multiple proliferation/mitosis-associated sets (Extended Data Fig. 3a, b). Additionally, MC mal macrophages showed increased expression of interleukin-10 (IL-10) and IL-4/IL-13 pathways, both described to be associated with a TAM tumor-supportive phenotype in multiple neoplasia, including diffuse glioma (Extended Data Fig. 3b)^31–33^. These results suggest that in low-risk *NF2*-mutant meningioma, innate immune cells are mostly of microglia or microglia-like phenotype, whereas in high-risk cases, those cells are less abundant, but myeloid-derived proliferative macrophages are enriched. At a functional level, these myeloid-derived cells show characteristics associated with tumor growth (that is, IL-10 and IL-4/IL-13 pathways) and therapy resistance (that is, SPP1).

To elucidate the tumor microenvironment (TME) further, we conducted spatial transcriptomics on 42 samples from 37 different meningiomas. We used a supervised neural network model to predict MCs based on local TME as defined by local gene expression. Specifically, we extracted 3-hop subgraphs from the spatial transcriptomic data, where each spot (node) is interpreted in relation to its neighboring and next-neighboring nodes, and trained a graph attention network (GAN) (Fig. 2h, left panel)^34^. The trained GAN achieved an overall accuracy at MC predicting of 0.974 (precision: 0.978, recall: 0.967, F1 score: 0.972) (Fig. 2h, middle panel and Extended Data Fig. 3c). Validation in an independent set of 16 published meningiomas resulted in a similar level of accuracy (0.93) but reduced precision (0.76), recall (0.78) and F1 score (0.76), possibly due to overfitting in the training dataset or due to the small validation dataset^35^. Dimensionality UMAP reduction of the latent space of spatial gene expression values further confirmed a clear separation of the different microenvironments (that is, spatial gene expression ecosystems) corresponding to the defined MCs (Fig. 2h, right panel). To explore individual cellular compositions associated with MCs, we performed single-cell deconvolution of each microenvironment subgraph. Here, for every node in the subgraph, the most likely cell of origin is determined by gene expression deconvolution (three examples are shown in Fig. 2i). Subsequently, subgraphs from individual samples were stratified by risk level to quantify and compare microenvironmental cellular compositions. This revealed and confirmed microglia-like cell reduction and enrichment of TAMs, reaching its highest abundance in MC mal subgraphs (Fig. 2j). This finding was validated when investigating activation pathways, as microglia differentiation and activation pathways were enriched in MC ben-1 networks, whereas we observed macrophage activation in MC mal (Extended Data Fig. 3d). These findings underscore that the TME is distinct when comparing low-risk to high-risk meningioma.

### Stromal signal contribution to meningioma ML classification

**Extended Data Fig. 4:**
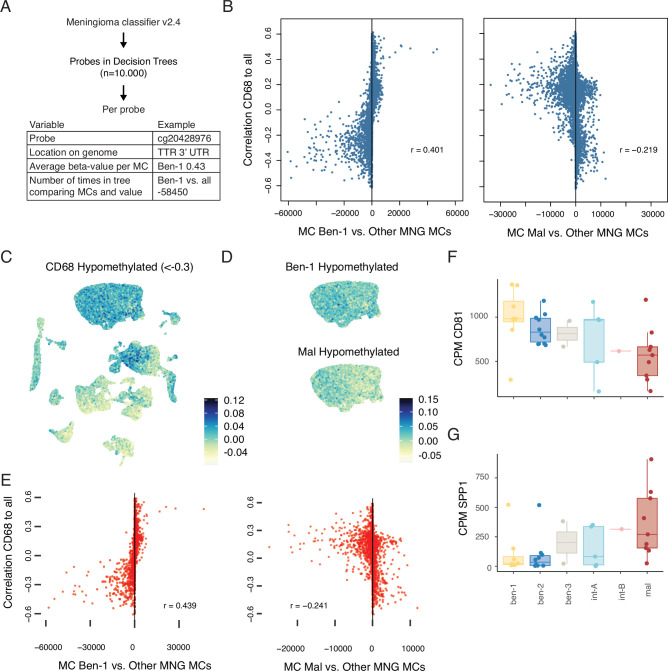
Meningioma classifier probes usage and CD68 staining patterns. Example of the working of the dedicated meningioma classifier that is based on 10,000 individual methylation probes. Probe usage values are inferred from the trained classifier, with probes that are used more often providing a better separation between classes. Negative values indicate that probes are hypomethylated, positive values indicate that probes are hypermethylated in the indicated class (**A**). Scatter-plot showing the correlation between the methylation probe beta-value and CD68 staining levels (y-axis) versus the probe usage in the classifier to separate MC ben-1 from other classes (x-axis). Scatter-plot on the right is showing the comparison of MC mal to other classes. (**B**). Genes associated with probes that are negatively correlated with CD68 protein level (that is, hypomethylated in samples with high CD68 abundance) show increased gene expression in TAMs (**C**). Visualization of snRNA gene expression in TAMs for genes associated with hypomethylated probes that separate MC ben-1 and mal (**D**). Genes with RNA-seq differential expression values of Log_2_FC > 1 or <1 and false discovery rate (FDR) adjusted p-value of 0.1 mapped to predictive probes for MC ben-1 (left) or mal (right) determination (**E**). Bulk RNA-seq expression levels show high expression of *CD81* in MC ben-1 (**F**) and high expression of *SPP1* in MC mal cases (**G**). Boxplots represent the upper and lower quartiles, while whiskers reach to the most outlying samples that are within 1.5 interquartile ranges (IQR) from the quartiles. Median values are labeled as horizontal lines in the boxes. For subfigures F and G, ben-1 *n* = 8, ben-2 *n* = 10, ben-3 *n* = 2, int-A *n* = 5, int-B *n* = 1 and mal *n* = 9.

Currently, molecular meningioma subtype classification is based on bulk DNA methylation and its interpretation by ML classifiers. Abundant TAMs, particularly in low-risk meningioma raises the question whether stromal DNA plays a role in molecular subtyping. To this end, we re-evaluated the random forest Brain Tumor Classifier that divides meningioma into the six MCs^7,36^. This subclassification is based on decision trees that split the dataset based on hypo- or hypermethylated probes (defined by the probe beta-value; Extended Data Fig. 4a)^37,38^. Design and outcomes of those systems have been described before and are endorsed for diagnostic application by the 2021 WHO classification of CNS tumors, National Comprehensive Cancer Network guidelines and European Association of Neuro-Oncology molecular testing guideline^3,37–39^. Even though correlations in relevant probes may not directly translate to biological processes, we wanted to investigate the role of TAMs in this classification. First, we investigated the correlation between CD68 TAM protein expression from the 120 stained meningioma and methylation states. Second, we quantified methylation probe usage during random forest classifier training (“probe usage” for every MC in Supplementary Table 1). This revealed that probes that separate ben-1 from other MCs were also correlated to CD68 abundance (Extended Data Fig. 4b). Interestingly, most of these probes were hypomethylated in ben-1, and their methylation state was negatively correlated to CD68 abundance, indicating that probes specifically hypomethylated in TAMs may underlie separation of ben-1. Conversely, probes separating MC mal from other subtypes were predominantly hypomethylated and positively correlated to CD68 abundance. To verify this, we visualized snRNA expression for genes associated with all CD68-hypomethylated probes or probes specific to MC ben-1 and mal. This revealed increased expression in TAMs, especially for MC ben-1-specific probes (Extended Data Fig. 4c, d).

Because MCs are identified based on bulk epigenetic profiles, we then screened probes mapping to up- or downregulated genes when comparing bulk RNA expression levels of MC ben-1 to MC mal (Extended Data Fig. 4e). This identified *CD81*, a gene involved in microglia proliferation (Extended Data Fig. 4f)^40^. By contrast, *SPP1* is present among the probes separating MC mal and highly expressed in MC mal bulk RNA-seq (Extended Data Fig. 4g), aligning with its aforementioned snRNA accumulation in MC mal-specific TAMs. However, due to the limited number of bulk RNA-seq profiles available, the *SPP1* expression difference compared to MC ben-1 did not reach statistical significance (log_2_ fold change 1.7, *P* = 0.10, false discovery rate (FDR) *P* = 0.21). Taken together, these results suggest that a large share of probes that underlie meningioma subclassification relate to genes with increased expression in TAMs, especially in MC ben-1, indicating that ML-based subclassification may rely on microenvironment-determined signals.

### Stromal and neoplastic epigenetic signatures

**Fig. 3:**
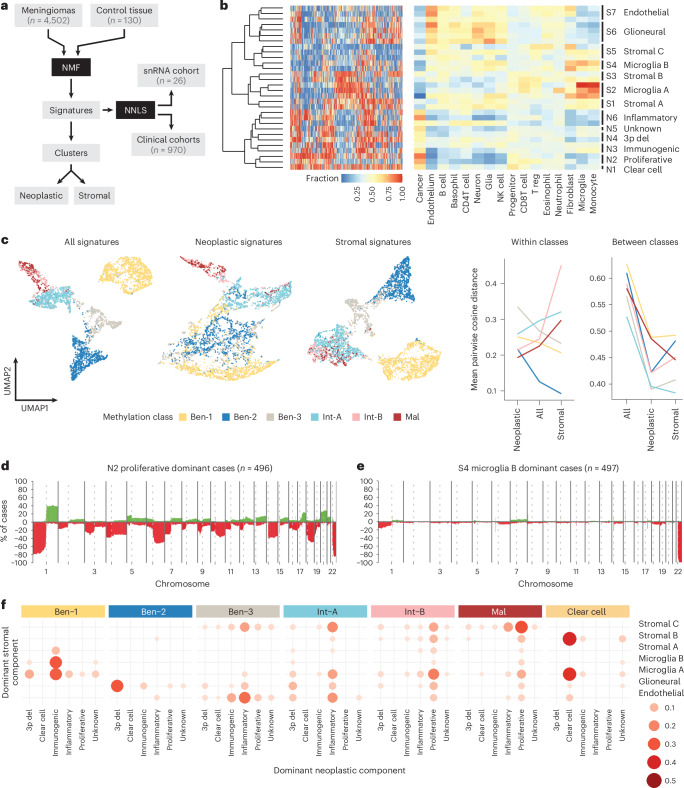
*NF2*-mutant meningiomas contain a mixture of neoplastic and stromal epigenetic signatures. **a**, Deconvolution using non-negative least squares (NNLS) and NMF analyses on a set of 4,502 meningiomas excluding (clinical) reference cases or epigenetic profiles from the snRNA cohort was performed to identify deconvoluted epigenetic neoplastic and stromal signatures. **b**, By matching the six neoplastic and seven stromal signatures to methylation profiles obtained from published isolated cell types, the signatures were annotated accordingly. **c**, UMAP visualization of 4,431 meningiomas using data from all signatures, or neoplastic signatures or stromal signatures alone, respectively, confirmed that TME stromal signatures contribute to subgrouping of meningioma (**c**, left panel). Mean pairwise cosine distance within or between MC (**c**, right panel). **d**,**e**, CNV profiles obtained from bulk methylomes dominated by a specific signature confirm either the high-risk profiles with multiple CNVs for the N2 proliferative signature (**d**) or the absence of many CNVs except for 22q where the *NF2* gene is located for S4 Microglia(-like) B stromal signature (**e**). **f**, Two-dimensional dotplots showing the dominant stromal and neoplastic signatures identified that the *NF2*-wild-type ben-2 and clear cell meningiomas feature a relatively homogeneous signature profile. The *NF2*-mutant meningioma, however, shares signals with multiple neoplastic and stromal signatures; the immunogenic signature peaks in MC ben-1, the low-grade *NF2*-mutant, and vanishes toward MC mal, in which the proliferative signature is dominant. del, deletion.

**Extended Data Fig. 5:**
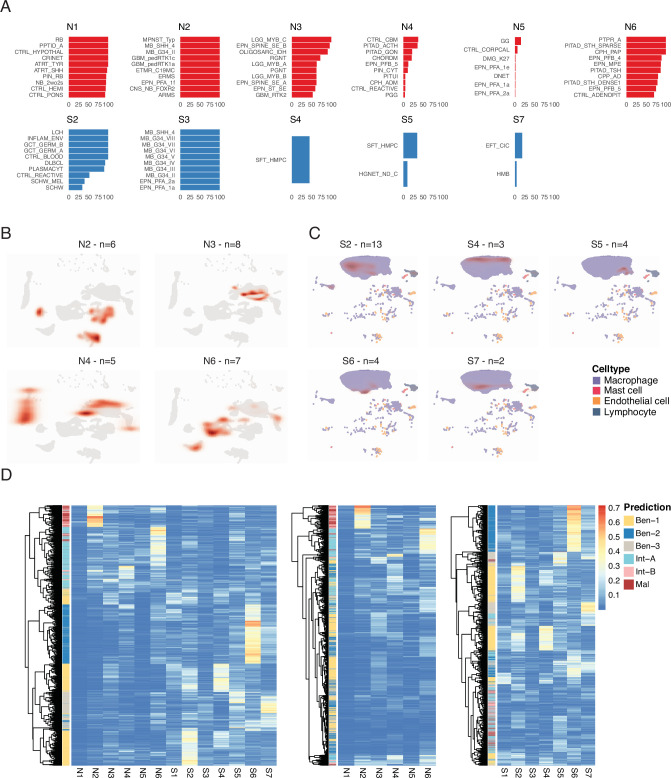
Deconvoluted neoplastic and stromal signatures in different epigenetic tumors and controls. The presence of the identified stromal and neoplastic signatures was investigated in 184 different (brain) tumour classes and control tissue in 7,495 methylation samples (**A**). Overlaying the location of cells originating from samples dominated by a specific neoplastic or stromal signature onto the snRNA UMAP identifies that cells from specific signatures cluster together confirming different expression patterns for the different signatures (**B** and **C**). Hierarchical clustering based on either combined (left panel), neoplastic (middle panel) or stromal signatures (right panel) identify that MC intermediate (int-A/int-B) and mal cluster together based on neoplastic signatures whereas benign MC cases cluster based on stromal signatures (**D**).

To further investigate the influence of microenvironmental DNA on subclassification, we performed deconvolution and non-negative matrix factorization (NMF) analyses on 4,502 bulk meningioma methylomes. These samples were not included in (sub)classification training in prior studies. Methylomes of snRNA cases and well-characterized clinical cohorts (discovery *n* = 506, retrospective validation *n* = 181 and prospective validation *n* = 283)^5,41^ were held back for validation (Fig. 3a). By applying NMF analysis, different methylation “signatures” were identified that were further subclustered and matched with published isolated cell types, yielding six neoplastic signatures (N1–N6) and seven stromal signatures (S1–S7) (Fig. 3b). In a different 7,495-methylome dataset of 184 CNS tumor and control tissues, similar enrichment was noted, including a higher signal for immune cells in mesenchymal glioblastoma subtypes (Extended Data Fig. 5a). Though suggesting similar relevance of non-neoplastic signal in the methylation classification of other tumor types, the initial deconvolution on meningioma data prevent exact feature identification. To confirm that the identified signatures represent the cellular composition, we overlaid signature dominance in matched snRNA/methylation array cases in either neoplastic cells (Extended Data Fig. 5b) or stromal cells (Extended Data Fig. 5c). This confirmed that different signatures cells cluster together, validating that the signatures share not only epigenetic but also transcriptional features. Next, we generated UMAP plots of 4,431 meningiomas (4,502 discovery cases minus 71 distinct *SMARCE1*-altered clear cell meningiomas) where either all signatures or only neoplastic or stromal signatures were included. Although sole neoplastic signatures cluster for individual MCs to some degree, this grouping was substantially more distinct when data from only the stromal signatures or the combined signatures was included (Fig. 3c). We quantified these differences by the mean pairwise cosine distance within and between MCs, where lower within-group and higher between-group distances generally suggest tighter within-MC clustering. Examining all signatures together generally accentuates between-class differences, but not within-class differences. Benign MCs, however, cluster more tightly when examining stromal signatures alone, and conversely, MCs int-A, int-B and mal cluster more tightly when examining neoplastic signatures alone (Fig. 3c). Furthermore, hierarchical clustering based on the combined, stromal or neoplastic signatures confirmed that MCs int-A, int-B and mal cases clustered together when using the neoplastic signatures and the benign MCs for stromal signatures (Extended Data Fig. 5d). This further demonstrates the contribution of TME signals to meningioma subclassification, especially in benign MCs.

**Extended Data Fig. 6:**
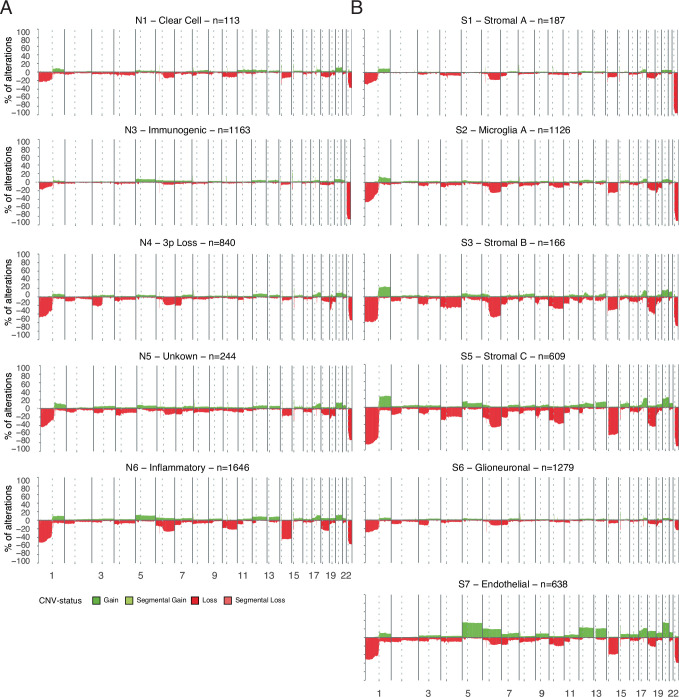
Copy-number profiles of cases dominated by specific neoplastic or stromal signatures. Meningioma cases dominated by a specific neoplastic (**A**) or stromal (**B**) signature were identified and corresponding copy-number profiles were plotted.

CNVs are characteristic features of meningioma MCs, and the number of CNVs increases with aggressiveness^5,42^. We therefore compared CNV profiles between signature enriched cases. Here, profiles from signature N2-dominated cases were associated with findings typical for high-risk meningiomas, namely numerous chromosomal deletions, including 1p, 6q and 14q (Fig. 3d and Extended Data Fig. 6a)^5^. Comparatively, stromal signature domination, such as S4 (“microglia(-like) B”), harbors a widely flat copy-number profile with an isolated loss of chromosome 22q (where *NF2* is located; Fig. 3e and Extended Data Fig. 6b)^43,44^. This confirms that high- and low-risk stromal and neoplastic signatures correspond to established risk-associated CNV profiles.

**Extended Data Fig. 7:**
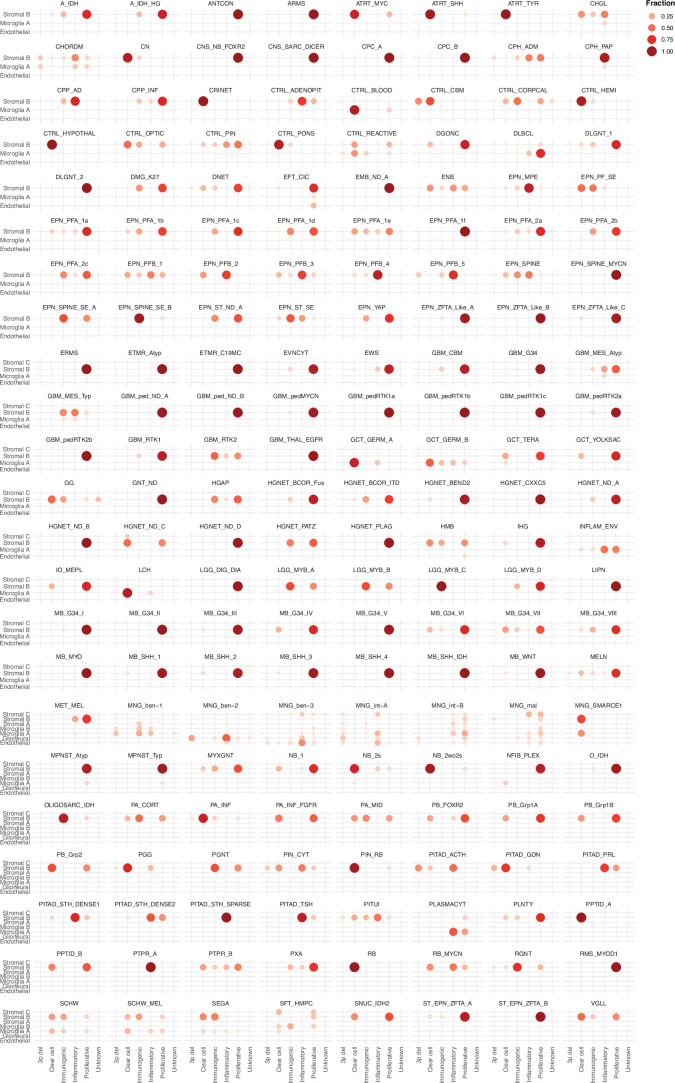
Stromal and neoplastic signatures identified in 184 different epigenetic classes. Two-dimensional dotplots showing the presence of the stromal and neoplastic signatures in 184 different (brain) tumour classes and control tissue in a total of 7,495 methylation samples.

**Extended Data Fig. 8:**
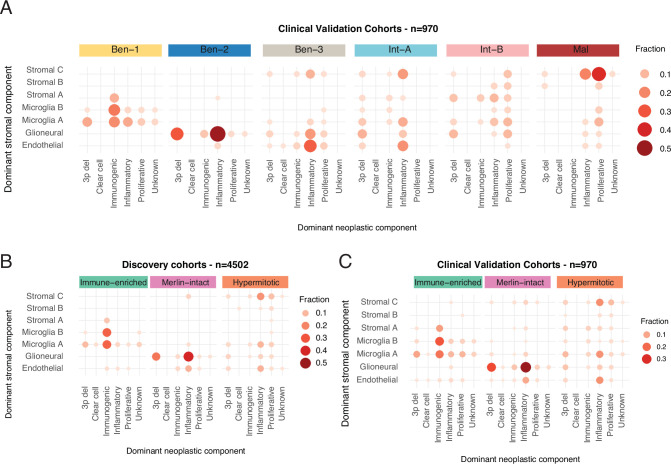
Stromal and neoplastic signatures in the methylation classes and methylation groups. Two-dimensional dotplots showing the presence of the stromal and neoplastic signatures in the methylation classes for 970 cases from the clinical validation cohorts (**A**), the UCSF methylation groups in the discovery set of 4,502 cases (**B**) or the UCSF methylation groups in the clinical validation cohorts (**C**).

Within the six MCs, and the rare, recently described MC of clear cell meningioma^45^, the number of dominant signatures varied strongly; *NF2*-wild-type, MC ben-2 and clear cell meningioma reflect a rather homogeneous profile (the full signature set for 184 CNS tumor classes is shown in Extended Data Fig. 7). *NF2*-mutant meningiomas, however, feature signals from multiple neoplastic and stromal signatures (Fig. 3f). Consistently, the N2 proliferative signature is the dominant neoplastic signal in MC mal, whereas MC ben-1 meningiomas have a high signal from microglia(-like) A and B signatures. Similar patterns were observed in the 970 validation cohort samples (Extended Data Fig. 8a) or when comparing MGs (Extended Data Fig. 8b, c).

### Meningioma subclones

**Fig. 4:**
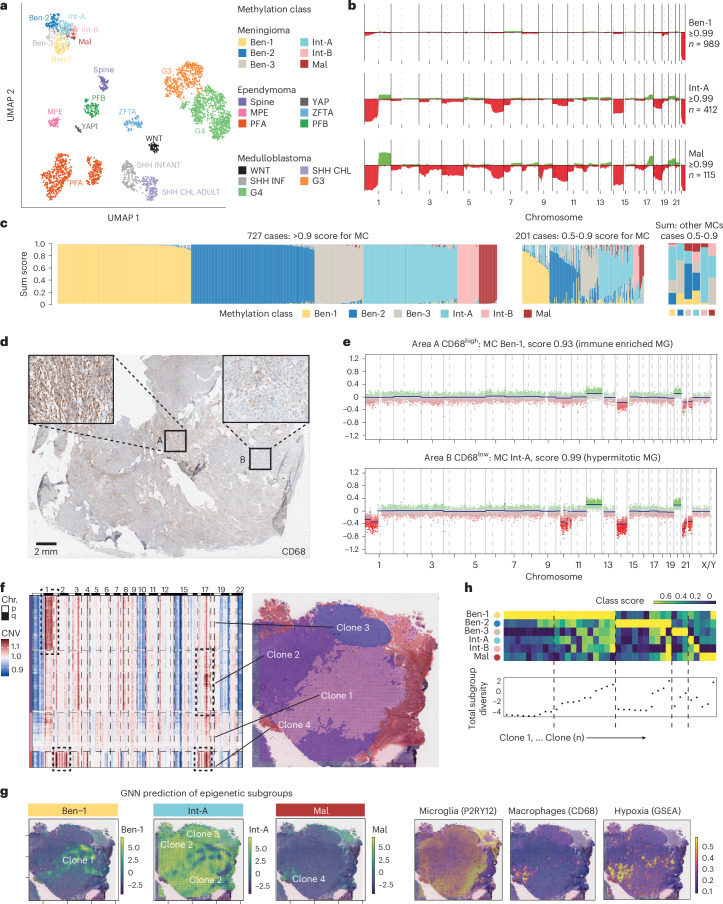
*NF2*-mutant meningioma is a spectrum rather than distinct tumor subtypes. **a**, To investigate if meningioma MCs are distinct tumor subtypes similar to the subtypes of ependymoma and medulloblastoma defined by different molecular driver alterations, 3,076 meningioma, ependymoma and medulloblastoma were plotted in a UMAP space. Medulloblastoma and ependymoma subtypes cluster individually, whereas meningioma MCs are confluent (spine, spinal NF2-mutant ependymoma; PFA/PFB, posterior fossa ependymoma type A/B; MPE, myxopapillary ependymoma; YAP, YAP1-fusion-positive ependymoma; ZFTA, ZFTA-fusion-positive ependymoma; sonic hedgehog (SHH) inf, medulloblastoma, SHH activated, infant type; SHH CHL AD, medulloblastoma SHH-activated adult; WNT, medulloblastoma WNT activated; G3/G4, medulloblastoma non-WNT, non-SHH group 3/group 4). **b**, Although meningiomas show this overlap at the epigenetic level, distinct CNVs are present in cases with a high, unequivocal prediction score of ≥0.99. **c**, In the clinical cohorts of 970 cases, a total of 928 cases have a score of ≥0.5 for one of the MCs. The majority of those cases have a score for one of the MCs of ≥0.9 (left panel). In a minority of the cases, there is a score between 0.5 and 0.9 for one MC and a lower score for one or multiple other MCs (middle panel). The *NF2-*mutant MCs cases 0.5 to 0.9, in general, have more leftover scores for other *NF2-*mutant MCs than the *NF2-*wild-type ben-2 MC (right panel). **d**, In a case with heterogeneous CD68 staining, indicative of heterogeneous TAM localization, two areas were selected with either a high (area A) or low (area B) number of TAMs (results from 29 additional analyses from different patients discussed in the main text). **e**, Methylation array data isolated from the two areas result in different MCs with a score >0.9. Additionally, the MC int-A area also contains an additional loss of 1p. **f**, Copy-number heatmap of an example case with the corresponding clonal hierarchical clustering of 4 distinct intratumoral subclones. **g**, Surface heatmaps demonstrate the subclass logit (logarithm of the odds) for the different MCs within the example case (left side). Additional surface heatmaps showing microglia (*P2RY12*) or macrophage (*CD68*) markers show co-localization of *CD68*-positive macrophages with the MC-mal prediction areas also enriched for hypoxia as determined by GSEA (right side). **h**, Heatmap of the clonal epigenetic subgroup logit-score diversity per clone out of 16 different tumors (top panel) and the total diversity (standard deviation of the logit score from each clone) (bottom panel).

**Extended Data Fig. 9:**
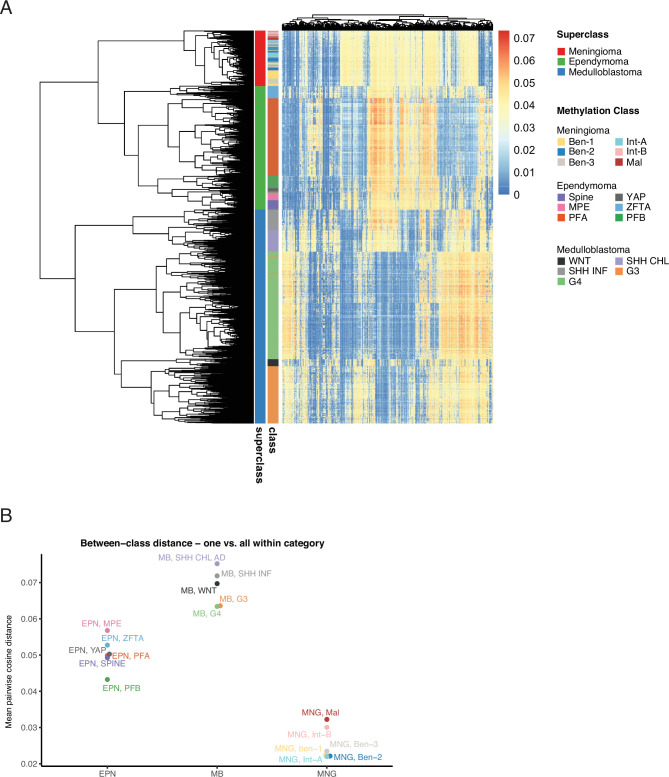
Hierarchical clustering and mean pairwise cosine distance analyses. We performed hierarchical clustering across 3,076 meningioma (MNG), ependymoma (EPN) and medulloblastoma (MB) cases which demonstrates how subclasses of MB and EPN cluster more neatly than those of MNG. An example of tree cut clustering (*k* = 20) shows that MB and EPN divide into subclusters whereas MNG stays uniform (**A**). The mean pairwise cosine distance for each methylation class within one disease group (ependymoma, medulloblastoma or meningioma). Spine – spinal *NF2*-mutant ependymoma, PFA/PFB – posterior fossa ependymoma type A/B, MPE – myxopapillary ependymoma, YAP – YAP1-fusion positive ependymoma, ZFTA – ZFTA-fusion positive ependymoma, sonis hedgehog (SHH) inf – medulloblastoma, SHH activated, infant type, SHH CHL AD – medulloblastoma SHH-activated adult, WNT – medulloblastoma WNT activated, G3/G4 – medulloblastoma non-WNT, non-SHH group 3/group 4 (**B**).

**Extended Data Fig. 10:**
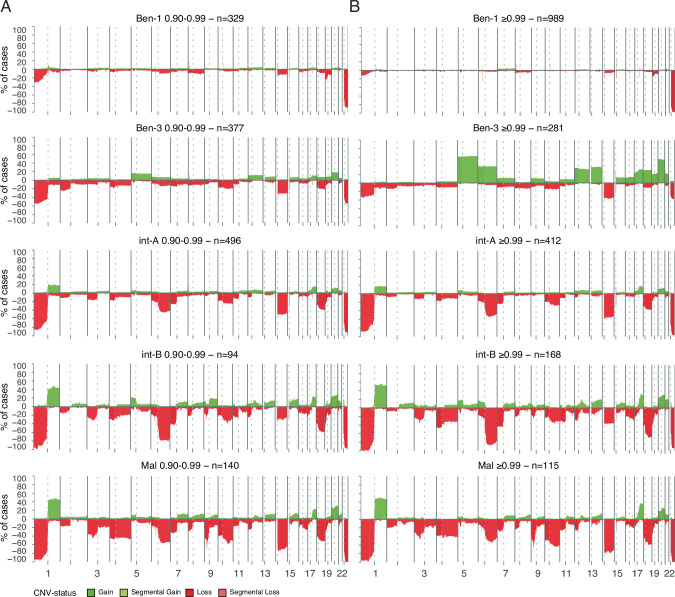
Copy-number variation (CNV) profiles in the different meningioma methylation classes divided by prediction score. Meningioma cases identified by calibrated prediction scores over the 0.9 threshold were split based on a score of 0.9-0.99 and ≥0.99. The corresponding CNV profiles were plotted for the different 0.9-0.99 cases (**A**) or the ≥0.99 cases (**B**).

Epigenetic meningioma subtyping is impacted by non-neoplastic cells. This is contrary to the concept of clear-cut tumor subtypes that are determined by different molecular driver alterations. The clinically highly relevant concept of molecular subtypes for CNS tumors, prototypically known from medulloblastoma (MB WNT-activated, SHH-activated, group 3/group 4)^46^ or ependymoma (EPN PFA, PFB, ZFTA fusion positive and YAP1 fusion positive)^47^, is rooted in distinct patterns of all determining tumor-intrinsic (CNVs, mutations and expression) and extrinsic factors (TME and localization) and results in a significantly different outcome. Although this applies to MC ben-2 (*NF2*-wild-type, skull base)^7^ meningiomas and clear cell (*SMARCE1*-altered)^45^ cases, the *NF2*-mutant meningiomas stretch out across a continuum of differentiation, host response and outcome. Accordingly, clustered MNG subgroups are evidently less segregated than medulloblastoma or ependymoma subtypes on the UMAP (Fig. 4a), as well as the hierarchical clustering dendrogram (Extended Data Fig. 9a), confirmed when calculating the mean pairwise cosine distances (Extended Data Fig. 9B). Even though meningioma may be epigenetically less distinct in subgroups, several previous studies have confirmed the predictive value of MCs^5,9,11^. Accordingly, different MCs indeed differ distinctively when comparing CNV profiles, especially when comparing cases with very high (≥0.99) calibrated prediction scores (Fig. 4b). Meningioma with scores above the clinically relevant 0.9 threshold but below 0.99 already show less distinct CNV profiles. Ambiguous CNV profiles may indicate underlying heterogeneity (Extended Data Fig. 10a, b). We therefore investigated whether tumor heterogeneity can be identified within bulk epigenetic profiles. In the brain tumor classifier, the total score for the meningioma methylation “family” is the sum of the different MCs^41^. First, for clinical cohort cases, we plotted MC scores in cases with at least one dominating (≥0.5) MC. Here, in 928 out of 970 cases, we identified that most profiles are dominated by a single MC with a score ≥0.9 (*n* = 727, 78.3%; Fig. 4c, left panel). Some 0.5–0.9 score cases have considerable scores for one or more additional MCs (Fig. 4c, middle panel). Further investigation of these cases (*n* = 201, 21,7%), revealed that only a small subset of *NF2-*mutant MC cases had concurrent scores for the *NF2-*wild-type MC ben-2. In turn, MC ben-2 cases with scores of 0.5 to 0.9 had admixed scores for MC ben-3, which includes both *NF2*-wild-type and mutant cases (Fig. 4c, right panel). In stark contrast, the purely *NF2*-mutant MCs ben-1, int-A/B and mal showed major overlaps. These data again suggest that *NF2-*mutant and *NF2-*wild-type meningiomas have distinct, separate epigenetic profiles, whereas *NF2*-mutant cases are only vaguely separable based on the tumor-intrinsic signal.

Having identified that the TAM number is associated with different MCs, we investigated meningioma cases with CD68 heterogeneity. Differential DNA extraction and methylation profiling obtained from either CD68^high^ or CD68^low^ areas revealed distinct, ≥0.90 scores for different MCs (Fig. 4d, e) in a case with ambiguous score in bulk whole-slide analysis. Specifically, the int-A CD68^low^ area contained the prognostic chromosome 1p loss (Fig. 4b)^48^.

To further characterize heterogeneity, we expanded the spatial RNA-seq analyses by applying InferCNV followed by hierarchical clustering exhibiting samples with genomic subclonal heterogeneity (an example with four subclones is shown in Fig. 4f, g and Supplementary Fig. 1a, b for MGs). Leveraging our pretrained spatial GAN model, we assessed the likelihood of MC assignment based on spatial architecture and cell-type subgraph composition (Fig. 4h). Analysis of 16 cases containing 38 different clones revealed that 44.7% of all samples exhibited heterogeneous spatial MC distribution, predominantly involving ben-1 and int-B. MC mal was generally homogeneously distributed in malignant samples, suggesting an homogeneous “end stage” of progression (Fig. 4h). This finding underscores the fact that meningioma can be heterogeneous with different subclones that individually may match to separate MCs.

Next, paired epigenetic analyses were performed on 29 representative tissue blocks split based on CD68^high^ and CD68^low^ areas. As expected, MC ben-1 cases obtained higher scores in the CD68^high^ isolations versus higher scores for MC mal in CD68^low^ isolations. In 8 of 29 cases (27.6%), a score >0.9 was obtained for only one of the isolations, and in five isolations, the effective molecular grading would have been higher. This finding underscores the value of CD68 staining to identify relevant areas for epigenetic analysis.

We then investigated how stable MCs are among primary and recurrent cases in 36 patients with more than one resection available. Naturally, cases that recur often initially present with higher risk MCs (that is int-A/B or mal) and show the same MC upon recurrence. However, there was significant MC switching between primary and recurrent cases within *NF2-*mutant MCs, whereas the only switching from a primary MC ben-2 case occurred to ben-3, the combined *NF2-*mutant and wild-type MC (Supplementary Fig. 1c and Supplementary Fig. 1d for MGs). This emphasizes the possibility of early or initial coexistence of molecular states, aligning with the snRNA data, the methylome deconvolution data and the strong correlation with TAM presence. In contrast, MB or EPN subgroups virtually never change within one case, even upon recurrence and under therapy^49^.

### Clinical impact of TME composition

**Fig. 5:**
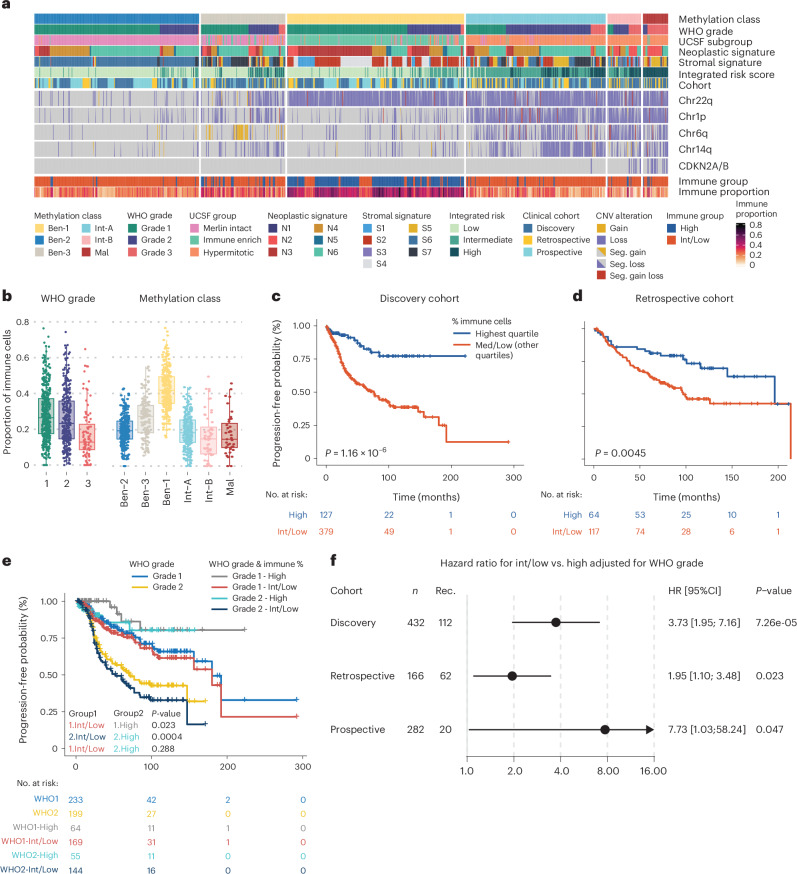
The number of immune cells in a meningioma can predict the risk for recurrence. **a**, To investigate the predictive value of the immune component in the different MCs, three previously described clinical cohorts, including a retrospective discovery, retrospective validation and prospective validation cohort, were investigated harboring cases from each WHO grade, MC, UCSF subgroup, stromal and neoplastic signature. For each case, the stromal and neoplastic signature with the highest score is annotated. **b**, Similar to the snRNA data, the number of immune cells as determined by methylation deconvolution decreased with aggressiveness of the WHO grade or MC (WHO grade 1 *n* = 527, grade 2 *n* = 353 and grade 3 *n* = 90. MC ben-1 *n* = 178, ben-2 *n* = 252, ben-3 *n* = 68, int-A *n* = 330, int-B *n* = 56 and mal *n* = 86). **c**,**d**, Kaplan-Meier analysis of progression-free survival for cases stratified by the quartile distribution of immune cell presence into a high (top quartile) or low (bottom)/intermediate (middle two quartiles) group resulted in a significantly different outcome in the discovery (log-rank test, Χ^2^ test statistic = 23.7, 1 degree of freedom) (**c**) and retrospective validation cohort (log-rank test, Χ^2^ test statistic = 8.1, 1 degree of freedom) (**d**). **e**, In the discovery cohort, the WHO grade 2 cases with high quartile immune cells had more favorable outcome than the combined WHO grade 1 group. Log-rank tests, all with 1 degree of freedom. Χ^2^ test statistic: 1.Int/low versus 1.High = 5.2, 2.Int/low versus 2.High = 12.6 and 1.Int/low versus 2.High = 1.1. **f**, Forest plot of WHO grade 1 and 2 meningioma indicating that the immune cell component remains a significant predictor after correction for the WHO grade in all 3 cohorts. Cox proportional hazard regression models were fitted to test the immune cell component using the Wald test. All tests were two sided, and no adjustment for multiple testing was done. Points indicate the hazard ratio, and horizontal lines indicate the corresponding 95% confidence intervals as estimated by the Cox proportional hazard regression model. For panels **c** to **e**, all tests were two sided. Boxplots represent the upper and lower quartiles, whereas whiskers reach to the most outlying samples that are within 1.5 IQR from the quartiles. Median values are labeled as horizontal lines in the boxes.

To investigate if TME composition impacts clinical outcome, we assessed bulk methylation data from the three clinical cohorts^5,41^ using a non-custom deconvolution method where each case was annotated for immune cell enrichment (that is, sum of TAMs and lymphocytes) defined as high versus intermediate/low (based on quartile distribution, Fig. 5a). Again, the highest immune cell enrichment was identified for WHO grade 1 and *NF2*-mutant low-risk MC ben-1 cases (Fig. 5b and Supplementary Fig. 2a for MGs). We then compared the clinical course between low, intermediate and high level cases. In all three cohorts, this stratification was significantly associated with differences in clinical course (Supplementary Fig. 2b, c). To simplify the model further and make it easier to apply in clinical practice where a dichotomous model is more applicable, we grouped the intermediate and low immune cases together. Again, a significant correlation was identified in all three cohorts (Fig. 5c, d and Supplementary Fig. 3a). In clinical practice, the accurate delineation between WHO grades 1 and 2 is the most pressing diagnostic issue. We therefore investigated whether immune cell levels can assist in this diagnostic challenge. This was true for the discovery cohort in which a high number of immune cells predicted a benign course for both WHO grade 1 and 2 cases, although the number of events was low (Fig. 5e). For the retrospective and prospective validation cohorts, the number of immune cells again separated cases within the WHO grades, but this was only significant for grade 1 in the retrospective and grade 2 in the prospective cohort (Supplementary Fig. 3b, c). Nevertheless, for WHO grade 1 or 2 cases, in each clinical cohort there was an increased risk for recurrence in intermediate/low immune cell meningioma, even when adjusting for the WHO grade (Fig. 5f). This effect is even more pronounced when leaving out the *NF2*-wild-type MC ben-2 cases (Supplementary Fig. 3d).

**Fig. 6:**
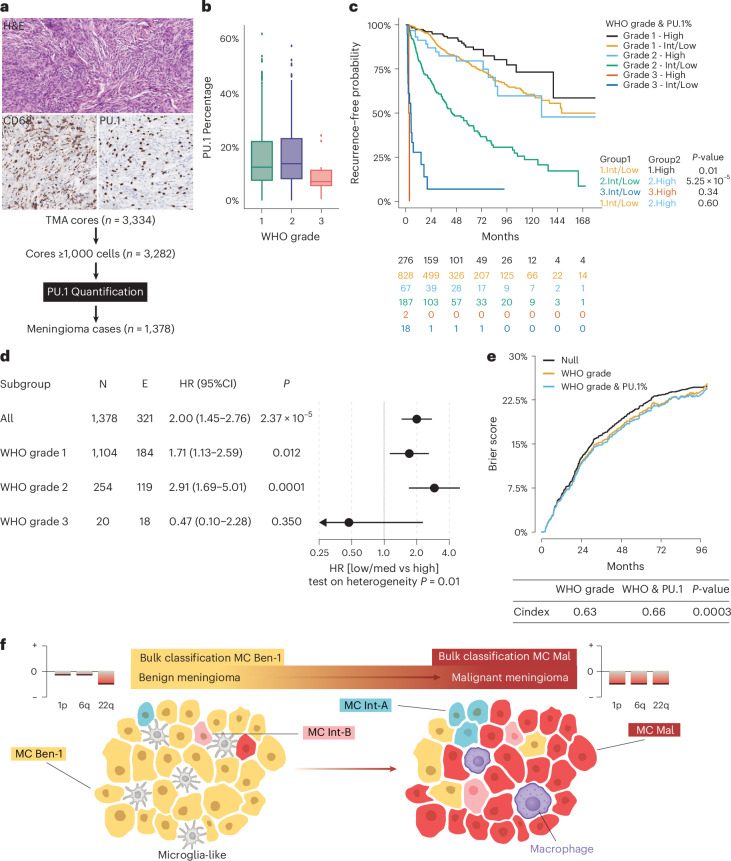
TAM cell quantification by immunohistochemical PU.1 staining can facilitate risk prediction in meningioma. **a**, The number of TAMs was quantified using CD68 and PU.1 immunohistochemical staining. PU.1, a nuclear staining, was selected for further evaluation of TAMs in a separate meningioma cohort of 1,378 patients. **b**, The percentage of PU.1 cells decrease with an increase of the WHO grade (WHO grade 1 *n* = 1104, grade 2 *n* = 254 and grade 3 *n* = 20). **c**, Kaplan-Meier analysis of progression-free survival for cases stratified by the quartile distribution of TAM/PU.1 cell percentage into a high (top quartile), or low (bottom)/intermediate (middle two quartiles). Log-rank tests, all with 1 degree of freedom. Χ^2^ test statistic: 1.Int/low versus 1.High = 6.5, 2.Int/low versus 2.High = 16.4, 3.Int/low versus 3.High = 0.9, 1.Int/low versus 2.High = 0.3. **d**, Forest plot indicating that the immunohistochemical PU.1 TAM quantification add significant value in WHO grade 1 and 2 cases. Cox proportional hazard regression models were fitted to test the immune cell component using the Wald test. All tests were two sided, and no adjustment for multiple testing was done. Points indicate the hazard ratio, and horizontal lines indicate the corresponding 95% confidence intervals as estimated by the Cox proportional hazard regression model. **e**, Brier prediction score analysis shows lower error rate, thus higher prediction accuracy in WHO grade 1 and 2 cases, for WHO + PU.1 versus WHO grading alone. There is also a significant difference in c-index accuracy observed (z-score test statistic −3.64). **f**, Graphical summary: *NF2-*mutant meningioma form a spectrum from benign low-risk tumors to malignant, high-risk meningioma. Within this spectrum, subclones of MC ben-1 (yellow), MC int-A (light blue), MC int-B (pink) and MC mal (red) exist as well as differences in both the number and composition of TAMs (that is, microglia-like dominated in benign cases to a limited number of macrophages in malignant meningioma). When only a limited number of malignant tumor cells are present that harbor CNVs associated with higher risk (for example, loss of 1p and/or 6q), these changes may not be detected in the bulk CNV plot but are present in individual aggressive subclones. For panels **c** and **e**, all tests were two sided. Boxplots represent the upper and lower quartiles, whereas whiskers reach to the most outlying samples that are within 1.5 IQR of the quartiles. Median values are labeled as horizontal lines in the boxes.

Quantifying the number of immune cells does not require methylation array deconvolution. We used simple immunohistochemistry to quantify the number of immune cells in an additional independent clinical cohort of *n* = 1,378 meningioma patients in a tissue microarray (TMA). To quantify TAM numbers, we stained for PU.1. PU.1 is a protein encoded by the *SPI1* gene, an ETS-domain transcription factor regulating gene expression in B cells, monocytes, macrophages and microglia^50^. The main advantage is the fact that PU.1 is a nuclear stain, facilitating accurate automated quantification (Fig. 6a). Again, a reduction in immune cells was observed in grade 3 meningioma over lower grade cases (Fig. 6b). More importantly, for both WHO grade 1 and grade 2 cases, there was a clear difference in progression-free survival after splitting the cases based on high versus intermediate/low quartiles (Fig. 6c). This difference in outcome is most pronounced in the often diagnostically ambiguous and clinically challenging WHO grade 2 cases with a hazard ratio of 2.91 [1.69–5.01, *P* < 0.001] for intermediate/low immune cases (Fig. 6d). The Q3 cutoff was identified at 22%, close to the optimal cutoff of 21% as identified by binary grouping of the data. The number of WHO grade 3 cases with a high number of immune cells was low (*n* = 2), forcing us to exclude WHO grade 3 cases from further analyses. For WHO grade 1 and 2 cases, the addition of PU.1-positive cells to WHO grade resulted in significantly more accurate prognostication compared to WHO grade alone (c-index 0.66 versus 0.63, *P* = 0.0003) (Fig. 6e). Together, these data show that assays for the number of TAMs can help predict risk for recurrence in meningioma patients.

## Discussion

In contrast to other tumor entities segregating into distinct subgroups, *NF2-*mutant meningioma consists of a continuum rather than previously proposed strictly separate subgroups. Based on this concept, the varying number of proposed subtypes is merely a result of granularity within this continuum. Likewise, the subgrouping does not identify a stable state within a tumor but one that is subject to change. Hence, progression despite prior “low-grade subgroup” assignment is not an outlier but is expected to occur in some instances. Our subclonal heterogeneity data also show that there is no group, subgroup or other marker that robustly predicts definitive long-term low risk among *NF2*-mutant meningioma, as for example the WNT group in medulloblastoma or the PFB group in ependymoma reliably indicates^46,47^. Of note, even though our results and conclusions are identified from multiple lines of investigations, functional experiments identifying the mechanism of the continuum are currently lacking. Our multimodal analyses, however, triggered further investigations into the TME. As TAMs are increasingly recognized as potential immunotherapy targets in neoplasia in general^51^, differences in meningioma TME components between low-risk and high-risk cases may indicate potential interventions may be subset specific.

By exploiting the fact that the number of immune cells differs between low-risk and high-risk meningioma, we devised a risk-stratification approach. Automated digital PU.1 staining percentage may be most accurate when available. More pragmatic may be the pattern of CD68, as homogeneous reticular microglial staining and elongated macrophages assure of low risk even in borderline cases between WHO grade 1 and 2, while focal or general lack of staining should alert for higher risk. Immunohistochemical markers for specific molecular subgroups have been proposed, but independent validation failed^6,52^. Rather than using immunohistochemistry to identify molecular subgroups, of which the existence is already debatable and a continuum may be better, the proposed PU.1/CD68 staining can be used for screening to identify areas of increased risk. This result may then trigger chromosome 1p analysis, sequencing or methylation assessment^2,5,53^.

It is intriguing to speculate on the mechanisms behind this phenomenon. Concurrent chromosome 1p/22q loss is now regarded as indicative of increased risk regardless of histology^2,53^. Chromosome 1p harbors the colony stimulating factor 1 (*CSF1*) gene. CSF1/CSF1R signaling is vital for microglial survival^54^, and thus, reduced CSF1 protein may be the cause for reduced microglia-like cell numbers in malignant meningioma. Further studies are warranted to investigate the CSF1/CSF1R axis in meningioma progression at a mechanistic level.

Together, our results show that not only the number but also the differentiation and activation of immune cells differ between low-risk and high-risk meningiomas (Fig. 6f). TME change has a similar influence on outcome as tumor cell-intrinsic features and is subject to incremental, continuous changes, challenging the model of subgroups among *NF2*-mutant meningioma. To align with established grading practice, a stratification for three risk groups, analogous to the three meningioma WHO grades, is most applicable. Given the wide clinical variation in *NF2*-mutant cases, as here shown stretching across a continuum, grading should cater for *NF2*-mutant cases in every tier. Additionally, our data suggest avoiding pinpointing risk on one molecular marker or parameter and further support the multiparameter risk prediction models combining histological and molecular data^4,5^. These combined requirements are best reflected in the integrated risk score (histology, CNV and methylation profiling)^5^.

More generally, deconvolution of methylation data, supported by single-cell data, provided unprecedented insight into the decisive features in epigenetic classifier approaches. This marks an essential advance in the explainability of this ML tool that is already used in clinical guidelines.

## Methods

### Ethics statement

Archival clinical cohort data analyses^5,41^, including data from collaborating hospitals in Germany, Austria, Switzerland, the United Kingdom and the United States, as well as tissue processing for RNA-seq, were performed in accordance with local ethics regulations (IRB Heidelberg, S-318/2022). Collection and generation of the meningioma TMA were performed in accordance with local regulations (IRB Tübingen, 618/2014BO2 and 191/2021BO2). No financial compensation was offered to the included patients. No exclusions were made based on age, sex, race or other (social) variable.

### Nuclei isolation from fresh frozen tissue

Samples were obtained from the archives of the Department of Neuropathology in Heidelberg, Germany for RNA isolation^55^. A total of 3 sections of 150 µm thickness were cut and transferred to an Eppendorf tube and resuspended in lysis buffer (320 mM sucrose, 5 mM CaCl_2_, 3 mM Mg(CH_3_COO)_2_, 2 mM EDTA, 0.5 mM EGTA, 10 mM Tris-HCl (pH 8.0), 0.1 % Triton-X and 1 mM DTT in water). The tissue was then grounded by using pestle A and B (Roth, product number CXE2.1). The subsequent lysate was then filtered twice (100 µm filter, 40 µm filter). To increase the recovery of nuclei, the tubes and filters were washed using a washing buffer in a total of 1 ml volume (320 mM sucrose, 5 mM CaCl_2_, 3 mM Mg(CH_3_COO)_2_, 2 mM EDTA, 0.5 mM EGTA and 10 mM Tris-HCl (pH 8.0) in water). To precipitate the nuclei, the buffer was centrifuged and the supernatant was then removed and the pellet resuspended in 600 µl washing buffer. To count, the nuclei were stained with AO/PI and subsequently quantified in a Luna-FL cell counter. The single-nuclei transcriptomics workflow was performed using the CG000315 Rev C (10x Genomics) protocol. Finally, sequencing was performed on an Illumina NovaSeq 6000 machine targeting at least 20,000 reads per nucleus.

### snRNA-seq data processing

Reads were aligned and quantified with CellRanger (v.6.1.1). We filtered cells that have more than 10% mitochondrial read counts or less than 200 expressed genes, as well as cells with the number of expressed genes more than 3 median absolute deviations greater than the median. We used DoubletFinder (v.2.0.3)^56^ to remove doublets, which was run on the first 15 principal components (PCs) with an expected doublet rate of 8%. Only samples that were processed in one batch were included, avoiding the need for batch correction.

Data processing was performed on the Seurat platform (v.4.3.0)^57^. Data were normalized with the SCTransform function under default parameters^58^. PC analysis was performed using the 3,000 most variable genes with a total of 50 PCs. The top 15 PCs served as the basis for UMAP and t-distributed stochastic neighbor embedding (t-SNE) visualizations, as well as Louvain clustering with a resolution of 0.6. The resulting clusters were manually annotated based on their canonical cell-type markers.

We identified meningioma class marker genes from neoplastic cells in the snRNA-seq data using COSGR (v.0.9.0)^59^, obtaining an RNA signature of 50 genes per class that are the most highly scoring and are expressed in at least 10% of neoplastic cells. RNA scores for each meningioma class were quantified at the single-cell level with the AddModuleScore function in Seurat.

Additionally, we constructed pseudobulk expression profiles by sample and cell type by calculating the sum of UMI counts across all cells. We performed differential expression analysis on the pseudobulk counts with the glmQLFit function in edgeR (v.3.32.1)^60^. Within each cell type, every MC is compared to all other classes.

### snRNA-seq CNV inference

We used InferCNV (v.1.17.0, https://github.com/broadinstitute/infercnv) to infer CNV variations from snRNA-seq data. Microenvironmental cells from all samples were used as reference. However, the number of TAMs were downsampled to 1,000 for balance. CNV levels were predicted with the HMM 3-state model. CNV-based tumor subclusters were identified using Leiden clustering on the inferred CNV matrix with resolution 3 × 10^−5^.

### TAM classification and analysis

We performed PC analysis on all tumor-associated microglia-like cells/macrophages with a total of 50 PCs. Cells from different samples were integrated with Harmony (v.1.0.3). UMAP and t-SNE visualizations were performed on the top 15 corrected PCs. Cells were clustered using Louvain clustering on the top 15 corrected PCs with a resolution of 0.3. Microglia-like cells were distinguished from peripheral macrophages using a set of markers: *C3, F10, EMILIN2, F5, GDA, MKI67, SELL* and *HP* for peripheral macrophages, and *TMEM119, P2RY13, P2RY12, GPR34* and *SLC2A5* for microglia-like cells. Expression levels of the marker sets were quantified with the AddModuleScore function in Seurat. TAM cells were labeled as macrophage-like or microglia-like based on the higher expression score between the two gene sets.

GSEA was performed using the fgsea R package (v1.24.0), where the genes were ordered based on the −log_10_(*P* value + 0.0000001)*logFC) statistic. The default hallmarks and reactome GSEA gene sets were used with *nPermSimple* = *100,000*, *minSize* = *10* and *maxSize* = *500* settings. The ComplexHeatmap package^61^ (v.2.14.0) was used for data visualization.

### Bulk RNA-seq data analysis

RNA data from 35 meningioma cases from the clinical cohorts of this study were extracted and processed as reported by Paramasivam et al.^62^. Differential expression analysis was performed on the count matrix with the glmQLFit function from the edgeR package. Genes with an FDR of less than 0.05 were considered differentially expressed.

### MIBI

Primary antibodies (Supplementary Table 2) were purchased in carrier-free solution (1× PBS, free of BSA and sodium azide) and subsequently validated by standard immunohistochemistry. Validated antibodies were conjugated to isotopic metal reporters^63^. Tumor sections were cut at 5 μm thickness, mounted on gold- and tantalum-coated microscopy slides and dried overnight. Tissue staining was subsequently performed; slides were baked at 70 °C, followed by deparaffinization and a standard rehydration series. Rehydrated tissue sections were subjected to antigen retrieval at pH 9 (Dako Agilent), 97 °C for 40 min. Unspecific antibody binding was prevented using a blocking solution for 1 h at room temperature.

Next, the antibody master mix was prepared in an antibody diluent (1× TBS-Tween 20 and 3% (v/v) normal horse serum). The master mix was then filtered with a 0.1 μm centrifugal filter and added to the samples for an overnight incubation at 4 °C (ref. ^64^). Lastly, slides were washed and fixed for 5 min in 2% glutaraldehyde solution prepared in low-barium PBS. After fixation, slides were washed in 0.1 M Tris at pH 8.5 and ddH_2_O and then consecutively dehydrated in a dehydration series. Vacuum-dried slides were stored until imaging.

Tissues were imaged using state-of-the-art multiplexed ion beam imaging (IonPath), which uses a bright ion source and orthogonal time-of-flight mass spectrometry to obtain ion counts with spatial coordinates from metal-tagged antibodies detected within 800 × 800 μm fields of view. Images were extracted and compensated using Toffy (https://github.com/angelolab/toffy) and visualized using ImageJ (v.1.5).

### Sources of bulk methylation data

We sought to perform cell-specific inference across a large number of bulk methylome profiles using data from a previous validated CNS tumor methylation classifier^37^. Methylation array data from all probe sites shared between Infinium HumanMethylation450K and MethylationEPIC v1.0 arrays were obtained across both training data samples and classified samples with a minimum random forest classification probability of greater than 0.9. To identify cell-type distinguishing probes, we obtained purified cell-type-specific methylation data from the Gene Expression Omnibus (GEO). Datasets included were GSE110554 (ref. ^65^) (B cells, CD4^+^ and CD8^+^ T cells, monocytes and natural killer cells), GSE112179 (ref. ^66^) (neurons), GSE122126 (ref. ^67^) (blood endothelium and neurons), GSE140295 (ref. ^68^) (blood endothelium), GSE167998 ref. ^65^ (B cells, CD4^+^ and CD8^+^ T cells, eosinophils, monocytes, natural killer cells, neutrophils and regulatory T cells), GSE184269 ref. ^69^ (B cells, CD4^+^ and CD8^+^ T cells, monocytes and natural killer cells), GSE191200 (ref. ^70^) (microglia), GSE63409 (ref. ^71^) (blood cell progenitors), GSE74877 (ref. ^72^) (endothelium and fibroblasts), GSE82234 (ref. ^73^) (blood endothelium), GSE84395 (ref. ^74^) (blood endothelium), and GSE88824 (ref. ^75^) (B cells, CD4^+^ and CD8^+^ T cells, monocytes, natural killer cells and neutrophils).

### Methylation post-processing

Raw signal intensities were extracted from IDAT files using the minfi Bioconductor package. Samples were normalized using background correction (shifting the 5% percentile of negative control probe intensities to 0) and a dye-bias correction (scaling the mean of normalization control probe intensities to 10,000) for both color channels. Correction for material type (formalin-fixed paraffin-embedded versus frozen) was performed by fitting univariate linear models to the log_2_-transformed intensity values using the removeBatchEffect function from the limma package. Methylated and unmethylated signals were corrected individually.

### Feature selection

To identify probes to delineate cell types present in CNS samples, we first identified pure CNS tumor samples from the MNP classifier dataset by applying RF_purify^76^ and a developed deconvolution^77^ to select the 500 most pure samples across all 124,162 distinct methylomes to represent the cancer cell set. We then pooled this with methylation data from all other cell types downloaded from GEO. We applied Methylcibersort^78^ to perform feature selection, which identified 1,574 probes that best distinguished cell types. We then paired this with probes that distinguish meningioma classes. To do so, we took the 10,000 probes utilized by the classifier and kept the 1,574 probes with the highest variance amongst all meningiomas and an additional 130 normal tissue control samples. Combining these with the 1,574 deconvolution probes, this defined a 3,148-probe set to distinguish between both cell types and meningioma subtypes.

### NMF

We performed non-negative matrix factorization with the aim of identifying latent methylation signatures operative in specific neoplastic and stromal programs. In matrix algebra, the problem is described by *V* ≈ *W*×*H*, where *W* represents the matrix of methylation signatures and, like beta values, its elements take on values between 0 and 1. Meanwhile, *H* represents the relative contributions from each signature to each sample, and its values also fall between 0 and 1, with the additional constraint that its columns each sum to 1.

For a *k*-component NMF, the values of *W* and *H* were initialized to random values drawn from the uniform distribution between 0 and 1, with the columns of *H* then normalized to sum to 1. We undertook a variant on the multiplicative update rules first proposed by Lee and Seung^79^ but applying corrective rules within each iteration to maintain compliance with the constraints.

1. $$W\leftarrow W\times \frac{V\times {H}^{{\rm{T}}}}{W\times H\times {H}^{T}}$$
2. $$W\left[W > 1\right]\leftarrow 1$$
3. $$H\leftarrow H\times \frac{{W}^{{\rm{T}}}\times V}{{W}^{T}\times W\times H}$$
4. Normalize columns of *H* to sum to 1

These steps were run repeatedly until convergence, defined as a change in the Frobenius norm by less than 0.01%.

To select the number of features, *k*, we undertook bicrossvalidation as described by Owen and Perry^80^. For each value of *k*, the matrix *V* is divided into four quadrant matrices: *A*, *B*, *C* and *D*. NMF is run as described above on quadrant D. Through self-consistency, quadrant A can then be derived as follows:

$${A=W}_{A}{H}_{A}=\left(B{H}_{D}^{+}\right)\left({W}_{D}^{+}C\right).$$

where ${Z}^{+}$ denotes the Moore-Penrose pseudoinverse of $Z$ and ${W}_{A},{H}_{A}$ and ${W}_{D},{H}_{D}$ denote the NMF fit to *A* and *D*, respectively. We repeated this operation four times for each value of *k*, rearranging rows and columns to yield four entirely distinct quadrant matrices for *A*, and sought the number of dimensions, *k*, which minimized the mean of the four Frobenius norms:

$${{\rm{argmin}}}_{k}{{||}A-{W}_{A}^{(k)}{H}_{A}^{(k)}{||}}_{2}.$$

### Deriving neoplastic and stromal signatures

NMF was performed on all methylomes available within the MNP classifier, which were classified as meningioma with a random forest probabilistic confidence of greater than 0.9. We held out 26 methylomes from cases in which we also performed snRNA-seq and 970 methylomes comprising cases in which we also had clinical follow-up data for use as validation cohorts. This yielded 4,502 meningioma cases for NMF. Signatures derived from NMF could thus be used to independently deconvolute these held-out methylomes in an unbiased manner. We performed hierarchical clustering over the matrix of NMF-derived patterns, *W*, and landed upon a sensible distance for merging similar patterns by examining the resulting dendrogram and the correlations of each pattern against deconvolution cell-type contributions. After merging, we classified each resulting signature as either predominantly stromal (S) or neoplastic (N) based on its relative similarity to cancer versus non-cancer cell signatures. We performed deconvolution using non-negative least squares (NNLS), using *W* as a reference matrix. Deconvolution was performed across many datasets: (1) the clinically annotated cohort and (2) the single-cell cohort, which were both held out from initial NMF, and (3) the complete labeled training data of the MNP methylation classifier consisting of 7,495 labeled methylomes from 184 distinct tumor types and normal control tissues. By examining the dominant signatures across these various classes, as well as associations with cell types and CNV profiles, we assigned a biologically motivated name to each of the neoplastic and stromal signatures.

### Meningioma classifier probe contribution

To identify probes that separate meningioma classes within the random forest classifier, we used the trained random forest model that is based on 10,000 CpGs^7^. We calculated the probe usage as the number of times a feature (that is, a DNA methylation probe) is selected to perform a split between tumor classes in the model^38^. Probe usage values were stored in a 3D array in which each layer (10,000 probes) is composed of a symmetric matrix representing the total probe usage of all six by six possible class comparisons. We further summarized probe usage values for one class versus all other classes. Negative values indicate that probes are hypomethylated at the splits for the indicated class, whereas positive values indicate that probes are hypermethylated at the splits for the indicated class. For these steps, the randomForest (v.4.7), data.table (v.1.14.2) and iterpc (v.0.4.2) R packages were used.

### Epigenome cell-type deconvolution for clinical modeling

Methylation data were deconvoluted to predict the proportion of the infiltrating immune cell compartment using the Edec R package v.0.9. To this end, a set of methylation array data from macrophages, CD3^+^ T cells, endothelial cells and fibroblasts^81–84^ served as cell-type references. Loci were restricted to those that were most informative by selecting the 500 most hyper- or hypomethylated loci (*run_edec_stage_0()*) with a *P* value of less than 0.00001 (*max_p_val* = *1e-5*) when comparing each reference cell type with the remaining reference cell types (*version* = *“one.vs.rest”*) in a *t*-test. Subsequently, the number of constituent cell types in the bulk methylation array samples was estimated (*estimate_stability()*) based on the informative loci by creating five random subsets of the samples (*num_subsets* = *5*) and including 80% of the data in each of these subsets (*subset_prop* = *0.8*) with a maximum of 800 iterations (*max_its* = *800*). Thereby an optimal number of three constituent cell types was obtained, which correlated to a population of immune cells including macrophages and T cells that could not be further discriminated, as well as two distinct populations of fibroblasts. The informative loci were further used to estimate the average methylation profile for each of the three constituent cell types and the respective proportion of the constituent cell types in the bulk methylation array samples (*run_edec_stage_1()*). The estimated proportion of the immune cell type was the basis for further analysis.

To perform unsupervised nonlinear dimension reduction of major ependymoma, meningioma and medulloblastoma known entities, 3,076 samples that achieved a calibrated score >0.9 with version 11b4 of the Heidelberg methylation array classifier^37^ were selected from our database. The 10,000 CpG probes with the highest standard deviation were selected, and a UMAP projection was calculated using the UMAP function available in the R package uwot (https://github.com/jlmelville/uwot). To show the methylation subclasses of meningioma, samples have been annotated using subclasses predicted by version 12.8 of the Heidelberg brain tumor classifier (https://www.molecularneuropathology.org/mnp/).

### Immunohistochemistry and quantification

To stain CD68 or PU.1 positivity via automated immunohistochemistry using the Ventana Benchmark ULTRA (Ventana Medical Systems), 4 µm-thick formalin-fixed paraffin-embedded sections were stained using CD68 antibody (Dako M0876, clone PG-M1) or PU.1 antibody (Abcam ab76543, clone EPR3158Y). Following deparaffinization and heat-induced antigen retrieval with CC1 (#950-500, Ventana), tissue samples were incubated with antibody of interest for 36 min for CD68 or 32 min for PU.1. Incubation was followed by OV or UV detection and hematoxylin II counterstain for 4 min and 20 min with blue coloring reagent. CD68-stained slides were digitized using an Aperio AT2 scanner (Leica), and PU.1 TMA slides were scanned using the Hamamatsu Nanozoomer HT2.0 using ×40 magnification.

Immunohistochemistry quantification was performed using the QuPath image analysis software. CD68 pixel surface quantification was performed as follows: after normalizing the stain vectors using “Preprocessing->Estimate stain vectors”, the following QuPath commands were executed: “Preprocessing → Simple tissue detection”, followed by “positive pixel count” in QuPath.

TMA slides (*n* = 57) stained for PU.1 were analyzed in QuPath. All cores were manually examined to control the accuracy of the cell detection function to exclude the cores or areas that do not feature enough meningioma tissue. Accordingly, *n* = 52 cores were excluded from analyses because less than 1,000 meningioma cells were present. Furthermore, areas within cores with extensive bleedings were excluded from analyses by drawing regions of interest to exclude PU.1-positive blood cells. The number of nuclear stains was subsequently assessed using the TMA dearrayer function followed by normalization using the “Preprocessing → Estimate stain vectors” commands for each TMA slide. Then, the percentage of positive nuclei was measured using the QuPath command “Analyze → Cell detection → positive cell detection”.

### Data import, preprocessing, filtering and normalization for spatial data analysis

For data analysis and quality control, we utilized the CellRanger pipeline (10x Genomics). A custom-built framework was implemented to facilitate the subsequent spatial data analysis. Spatial transcriptomic data was imported into the SPATA tool using the function SPATA::initiateSpataObject_10X, or manually by providing count matrices, barcode-coordinate matrices and hematoxylin and eosin (H&E) staining images. During the import process, gene expression normalization was conducted using the Seurat v.4.0 package, which involved scaling the transcript counts per spot to 10,000 and applying a natural log transformation. Batch effects were adjusted by regressing out sample-specific batch influences, along with the percentage of ribosomal and mitochondrial gene expression.

### Spatial sequencing CNV estimation

CNA analysis was conducted using a pipeline integrated into the SPATA2 R tool (development version available at https://github.com/theMILOlab/SPATA2). The estimation of CNVs was performed by mapping genes to their chromosomal locations and calculating a moving average of expression values across a 100-gene sliding window, with gene order determined based on genomic position via the InferCNV package. As a reference, spatial transcriptomic data from non-malignant cortical tissue were used. To enhance computational efficiency, optional down-sampling was used. Outlier gene expression values were capped within the range of [−2.6, 2.6] to mitigate their impact on CNV estimation, with InferCNV used for these adjustments. Following estimation, the CNV values were spatially aligned and clustered using SPATA’s data slots and functions. The function SPATA::joinWithFeatures() was used for cluster-specific analysis, and additional CNV analysis was facilitated via the command SPATA2::runCnvAnalysis(). Chromosomal bins were created using the SPATAwrapper Create.ref.bins() function, with a bin size of 1 Mbp. For this study, rescaling and interpolation were performed over a 10-kbp window, and data normalization was achieved using a loess regression model via SPATAwrappers::runCNV.Normalization().

### Spatial autocorrelation Moran’s *I*

We evaluated spatial dependencies between spots using Moran’s I statistic, which helps determine whether gene expression patterns within the sample are spatially clustered, randomly distributed, or dispersed. A positive Moran’s *I* value indicates spatial clustering, whereas a negative value suggests spatial dispersion. The Moran’s *I* index is calculated using the following formula: $I=\frac{N}{W}\times \frac{{\sum }_{i}{\sum }_{j}{w}_{{ij}}\left({X}_{i}-\bar{X}\right)\left({X}_{j}-\bar{X}\right)}{{\sum }_{i}{\left({X}_{i}-\bar{X}\right)}^{2}}$ Where $N$ is the total number of spatial spots, and ${X}_{i}$ and ${X}_{j}$ are the gene expression values at spots *i* and *j*, respectively. $\bar{X}$ is the mean gene expression value across all spots. ${w}_{{ij}}$ represents the spatial weight between spots *i* and $j$. $W$ is the sum of all spatial weights ${w}_{{ij}}$. The computation of Moran’s *I*, including the spatial weights matrix, is performed using the ‘inferSpatial.ac()’ function from the SPATAwrappers package.

### Single-cell deconvolution using Cell2location

Our analysis began by converting the SPATA object into the AnnData format to ensure compatibility with the Scanpy toolkit in Python. This conversion served as a preparatory step for the Cell2location model setup^85^. Mitochondrial genes, identified by their ‘MT-’ prefix, were extracted and stored in the obsm[‘MT’] matrix. We then aligned the variable names in the AnnData object with those in the inferred average expression data frame (inf_aver), which represents single-cell estimations, by filtering for shared genes. The AnnData object was then prepped for Cell2location using the setup_anndata function, where we defined key parameters such as the number of cells per spatial location and detection sensitivity. To ensure computational efficiency, we trained the Cell2location model for 500 iterations on a GPU. After model training, the export_posterior function was used to retrieve the posterior distribution of cell-type proportions, based on 1,000 samples. These samples provided accurate estimates of cell-type distributions across the spatial framework. The median estimates of cell-type abundance were recorded in adata_vis.obsm[“q05_cell_abundance_w_sf”]. Finally, the cell-type abundance data were integrated back into the SPATA object using SPATA2’s addFeature function, allowing for downstream analysis with this newly incorporated information.

### Construction of spatial graphs from Visium spatially resolved transcriptomic data

Each spatial transcriptomic dataset was preprocessed using SPATA2, which included log-transforming the count matrix and aligning the H&E image dataset. Nucleus positions were annotated using an automated segmentation algorithm based on a pre-trained model in Ilastik. For samples with lower imaging quality where automated segmentation was unsuccessful, we applied an adapted version of the CytoSpace algorithm. We then extracted the spot coordinates using the getCoordsDf function from the SPATA2 package and computed a pairwise distance matrix based on the ‘x’ and ‘y’ coordinates of each cell. To prevent computational errors from zero values (representing a cell’s distance to itself), these were replaced with a constant value of 1000, ensuring that thresholding steps wouldn’t mistakenly consider a cell as its own neighbor. The next step involved determining a distance threshold to define cell adjacency. This threshold was set to one unit greater than the smallest non-zero distance in the adjusted distance matrix. Using this threshold, we constructed an adjacency matrix, where cells within the threshold distance were marked with ‘1’ (indicating adjacency) and those beyond the threshold were marked with ‘0’ (indicating no adjacency). This adjacency matrix was converted into an undirected graph using the graph_from_adjacency_matrix function from the igraph package, with rows and columns labeled by unique barcodes identifying each cell, as retrieved from getCoordsDf. To link the spatial data with gene expression, we extracted the gene expression matrix from the object (obj) and transposed it to align with the graph vertices. We subsetted the expression matrix to include only the 5,000 most variable genes, corresponding to the labeled vertices of the graph. This integration of spatial and expression data enabled further analyses of gene expression patterns in relation to spatial cell distribution. To characterize the local topology around specific spots, we defined a ‘query spot’ and identified its n-hop neighborhood, focusing on the 3-hop neighborhood (spots reachable within three edges from the query spot). This approach provided insights into the immediate spatial context by considering the connectivity of spots within a defined graph distance.

### GNNs from the NePSTA

The graph neural network (GNN) used in this study is adapted from the NePSTA project (available at https://github.com/heilandd/NePSTA). To evaluate NePSTA’s performance and compare it with alternative methods, we used our Visium dataset. A stratified approach was implemented to partition the datasets into training and evaluation subsets. For data split, the dataset included 42 samples from 37 meningioma tumors, which were divided into training and validation subsets. For datasets containing only a single specimen, spots were manually segmented using the createSegmentation function from the SPATA2 package. The training dataset was created using the PyTorch Geometric library by selecting up to 500 subgraphs from the training split. These subgraphs were categorized based on epigenetic tumor types (for example, ben-1, ben-2 and ben-3), resulting in a total of 36,300 subgraphs for training. To evaluate dataset construction, we utilized validation subsets spanning a range of epigenetic classes. From each validation dataset, up to 200 subgraphs were extracted using the 3-hop neighborhood method, providing comprehensive coverage for model evaluation.

### Evaluation metrics

To assess the performance of the GNN in predicting clinical and histological parameters, we used a range of evaluation metrics.

#### Accuracy

Accuracy reflects the proportion of correct predictions among the total number of predictions, providing a measure of overall model performance.

$$\mathrm{Accuracy}=\frac{\text{Number of Correct Predictions}}{\text{Total Number of Predictions}}$$

#### Precision (macro average)

Precision evaluates the model’s exactness, calculated as the ratio of true positives (TP_*i*_) to the total positive predictions, where FP_*i*_ represents false positives. In our case, a macro-average approach was used to treat all classes equally, irrespective of class imbalance:

$${\mathrm{Precision}}_{\mathrm{macro}}=\frac{1}{N}\mathop{\sum }\limits_{i=1}^{N}\frac{{\rm{TP}}_{i}}{{\rm{TP}}_{i}+{\rm{FP}}_{i}}$$

#### Recall (macro average)

Also known as sensitivity, recall measures the model’s completeness, calculated as the ratio of true positives to the total actual positives, where FN_*i*_ represents the false negatives:

$${\mathrm{Recall}}_{\mathrm{macro}}=\frac{1}{N}\mathop{\sum }\limits_{i=1}^{N}\frac{{\rm{TP}}_{i}}{{\rm{TP}}_{i}+{\rm{FN}}_{i}}$$

#### F1 score (macro average)

The F1 score is the harmonic mean of precision and recall, providing a balanced measure between false positives and false negatives:

$$\mathrm{F}1\,{\mathrm{Score}}_{\mathrm{macro}}=2\times \frac{{\mathrm{Precision}}_{\mathrm{macro}}\times {\mathrm{Recall}}_{\mathrm{macro}}}{{\mathrm{Precision}}_{\mathrm{macro}}+{\mathrm{Recall}}_{\mathrm{macro}}}$$

Additionally, we presented a confusion matrix, which offers a detailed breakdown of the model’s predictions across different classes, including true positives, true negatives, false positives, and false negatives.

### NePSTA GNN architecture

The NePSTA network consists of a graph attention network (GAN) backbone, with multiple multilayer perceptron (MLP) heads designed for various prediction tasks. Inputs to NePSTA consist of local spatial graph structures based on gene expression values from the 3-hop neighborhood of Visium spots. Each spot is defined as a node, and edges are established between direct neighbors, all implemented using the PyTorch Geometric library. Node Features: The node features are encapsulated by an $N{\times }G$ matrix, where $N$ denotes the number of nodes in a given subgraph and $G$ represents the set of genes. These features are derived from the log-scaled, normalized expression values of the top 2,000 most variably expressed genes across the cohort. To account for the zero-inflated distribution typically observed in spatially resolved gene expression datasets, nonexpressed genes (that is, those with zero counts) are masked. This step prevents the inclusion of noninformative features, ensuring that the analysis remains focused on meaningful variation. Edge Features: Edges in the graph represent spatial connections between nodes, with each node linked to up to six neighbors to reflect the physical arrangement of cells in the spatial transcriptomics data. To maintain structural complexity, subgraphs with fewer than 15 nodes are excluded from analysis. Additionally, self-loops—where each node is connected to itself—are incorporated as part of the graph’s edge features. These self-loops are essential for retaining the node’s original feature information during the message-passing phases of the GNN, particularly during the forward pass.

### GNN architecture

The GNN contains two layers of GANs with a latent space of 1,024×5 hidden features, five attention heads and two sequential layers. For each node *i* in a graph with its neighbors $j\in {\mathscr{N}}(i)$, the attention mechanism learns how much “attention” each neighboring node should receive based on their feature representations. Let ${{\bf{h}}}_{i}\in {{\mathbb{R}}}^{F}$ be the feature vector of node *i*, ${{\bf{h}}}_{j}\in {{\mathbb{R}}}^{F}$ be the feature vector of node $j$ and ${\bf{a}}\in {{\mathbb{R}}}^{2F}$ the attention weight vector. The attention score ${e}_{{ij}}$ between node *i* and node $j$ is computed as ${e}_{{ij}}={\rm{LeakyReLU}}\left({{\bf{a}}}^{T}\left[{{\bf{Wh}}}_{i}{||}{{\bf{Wh}}}_{j}\right]\right)$, where || denotes concatenation and ${\bf{W}}$ is the learnable weight matrix for the linear transformation of node features. The attention weights ${\alpha }_{{ij}}$ are computed using the softmax function to normalize the attention scores across the neighbors of node *i*:

$${\alpha }_{{ij}}=\frac{\exp \left({e}_{{ij}}\right)}{\mathop{\sum }\limits_{k\in {\mathscr{N}}\left(i\right)}\exp \left({e}_{{ik}}\right)}$$

Once attention weights are computed, the output feature representation for each node is a weighted sum of its neighbors’ features. In your architecture, we have 5 attention heads. The output from multiple attention heads is concatenated or averaged.

$${{\bf{h}}}_{i}^{{\prime} }={{\rm{||}}}_{k=1}^{K}\sigma \left(\mathop{\sum }\limits_{j\in {\mathscr{N}}\left(i\right)}{\alpha }_{{ij}}^{\left(k\right)}{{\bf{w}}}^{\left(k\right)}{{\bf{h}}}_{j}\right)$$

where *σ* is a non-linear activation and *K* the number of attention heads $(K=5)$. The latent space finally contains 1,024 features. Between the GA and sequential layers we performed batch normalization and added a dropout. Additionally, we added a decoder (4 sequential fully-connected layers) which reconstructed the latent space into the gene expression feature matrix. Weights are initialized using the Xavier uniform method. The resulting features are merged into a latent space, and then global mean pooling is applied to create graph-level representations.

For the prediction tasks, separate MLP modules are used. Each MLP consists of a linear layer, a ReLU activation, batch normalization, dropout and a final linear layer that outputs the predictions. The MLPs are structured as follows:

$$h\left(x\right)={W}_{2}\times D\times B\times \phi \left({W}_{1}\times x+{b}_{1}\right)+{b}_{2}$$

where $x$ is the latent space vector to the MLP. ${W}_{1}$ and ${W}_{2}$ are the weight matrices for the first and second linear transformations, respectively. ${b}_{1}$ and ${b}_{2}$ are the bias vectors for the first and second linear transformations, respectively. $\phi$ denotes the ReLU activation function, applied element-wise, where ${\phi }_{z}={ma}{\rm{x}}\left(0,z\right)$, $B$ represents the batch normalization operation applied to the activated output. $D$ represents the dropout operation which randomly zeroes some of the elements of its input with a certain probability to prevent overfitting. We applied different loss strategies for individual prediction heads (MLPs) that were integrated. For categorical variables, we used cross-entropy loss, which is calculated for each class and summed, where for each sample and class pair, it combines $\log \left(\rm{softmax}\right)$ operations:

$$\mathrm{CrossEntropyLoss}=-\mathop{\sum }\limits_{c=1}^{M}{y}_{o,c}\log \left({p}_{o,c}\right)$$

### Model training procedure

The training of the model was conducted over a predefined number of epochs, encapsulated within a progress-tracking loop. For each epoch, the model iterated through batches of data provided by the loader. Within each iteration, the optimizer’s gradients were first reset to zero to prevent accumulation from previous forward passes. The model then performed a forward pass by processing the input data, which had been moved to the appropriate computing device, yielding latent representations and predictions for survival, neuron scores and status outcomes. The neuron score predictions were assessed against the ground-truth neuron scores using the L1norm loss function. This loss was then propagated backward through the network to compute the gradients. Following this, an optimization step was taken, adjusting the model’s weights to minimize the aggregate loss. Adam optimizer was used to minimize corresponding losses in different tasks.

### Evaluation of the subgraph cell composition

To reconstruct the cellular composition of each subgraph, we first identified the cellular positions as described earlier and estimated the likely cellular composition by integrating cell counts per spot with deconvolution scores from Cell2location, using the spAnchor package for implementation. We started by pinpointing the spatial location of each nucleus via the getNucleusPosition function from SPATAwrappers and recorded spot coordinates using SPATA2’s getCoordsDf function. The spatial coordinates of nuclei were represented as $P=\{{{{p}}}_{{{i}}}| i=1,\ldots ,N\}$, where ${p}_{i}$ is the coordinate pair for the ${i}^{\rm{th}}$ nucleus and $N$ is the total number of nuclei. Spatial grid coordinates corresponding to the transcriptomics data points were retrieved, denoted as $G=\{{{{g}}}_{{{j}}}| j=1,\ldots ,M\}$, with each $g_{j}$ representing the coordinate pair for the ${j}^{\rm{th}}$ grid point. For each grid point $g_{j}$, a vector of deconvolution scores ${{\bf{D}}}_{{{j}}}=\{{{{d}}}_{{{jk}}}| k=1,\ldots ,T\}$ was extracted, where ${d}_{{jk}}$ represents the score for the ${k}^{\rm{th}}$ cell type at grid point $j$ and $T$ is the number of cell types. The scores were normalized to a range of [0, 1], and the number of cells of each type at each grid point was estimated as:

$${{{C}}}_{{jk}}={\mathrm{round}}\left(\frac{{{{d}}}_{{jk}}^{{\prime} }\times {{{N}}}_{{{j}}}}{{\sum }_{{{k}}=1}^{{{T}}}{{{d}}}_{{jk}}^{{\prime} }}\right)$$

where ${{{d}}}_{{{jk}}}^{{\prime} }$ is the normalized score and $N_{j}$ is the number of cells at grid point $j$. Cell types were assigned to each grid point $g_{j}$ to create a mapping $M_{j}$, correlating grid points with their respective cell types. This cell-type mapping was integrated with nucleus position data, resulting in a comprehensive spatial map of cell-type distribution: $S=\{\left({{{p}}}_{{{I}}},{{{M}}}_{{{j}}}\right)| {{{p}}}_{{{i}}}\in P,{{\rm{M}}}_{{{j}}}\in M\}$. This methodology allows for the visualization and analysis of cellular composition within the tissue section, providing detailed insights into the spatial organization of the cellular environment.

### Clinical cohorts and predictive modeling

Meningioma methylation (sub)class scores were obtained using the v12.5/v12.8 brain tumor classifier (www.molecularneuropathology.org). Because clear cell (*SMARCE1*-altered) meningioma is not compatible with the integrated risk score, *SMARCE1*-altered meningiomas were excluded from the clinical cohorts for accurate comparisons^41^. This resulted in a retrospective discovery cohort of 506 cases, a retrospective validation cohort of 181 cases and a prospective validation cohort of 283 patients.

To annotate cases with the UCSF meningioma subtypes, a linear support vector machine was trained to predict the subtypes according to the code and annotated training data reported by Choudhury et al.^9^. The Illumina MethylationEPIC (850k) array UCSF training data therefore had to be restricted to loci present in the Illumina HumanMethylation450 (450k) array that was obtained for some cases in the current dataset. The linear support vector machine was then applied to the in-house methylation array data, generated as described above, and each sample was then assigned to one of the three UCSF subtypes.

Copy-number information was determined by a customized pipeline based on the conumee R package^41,53^. Chromosomal arms with segmental gains or losses over 95% of the arm were score as “gain” or “loss” respectively. Arms with losses/gains between 5% and 95% of the arm as “segmental loss”, “segmental gain” or “segmental loss and gain” and cases with less than 5% of gains or losses as “balanced” to account for technical noise. Summarization plots of CNV profiles were generated by dividing the chromosomal arms into 1% segmental bins and by subsequently determining the CNV status for each bin as described above.

The crossover between MCs between primary and recurrent meningioma cases was visualized using the ggsankey v.0.0.99 and ggplot2 v.3.4.3 packages for R. The clinical cohorts were visualized using the ComplexHeatmap v.2.14.0 package for R^61^.

Kaplan-Meier estimates and Cox regression were used for analysis of time to recurrence. To determine and assess discrimination and prediction performance of risk, Harrell’s c-index and Brier score were assessed and subsequently compared^86^. All statistical tests were two sided. Obtained *P* values below 0.05 were considered significant. All analyses were performed using R (v.4.4.2) with add-on packages riskRegression (v.2023.12.21), compareC (v.1.3.2) and forestplot (v.3.1.6).

### Statistics and reproducibility

No statistical methods were used to predetermine sample sizes. To ensure reproducibility, all experiments were performed using biological replicates as outlined in the paper. Where possible, findings were validated using complementary techniques targeting different molecular levels in independent datasets (for example protein level validation of snRNA-seq data in a different set of meningiomas).

An earlier batch of snRNA samples with suboptimal isolation and low reads per cell was excluded after subsequent optimization of protocol yielded substantially higher quality-control values.

Randomization of the data was not applicable. The investigators were not blinded during data analysis.

## Online content

Any methods, additional references, Nature Portfolio reporting summaries, source data, extended data, supplementary information, acknowledgements, peer review information; details of author contributions and competing interests; and statements of data and code availability are available at 10.1038/s41588-025-02475-w.

## Supplementary information

Supplementary Information Supplementary Figures 1–3, The German “Aggressive Meningiomas” Consortium (KAM) members.

Peer Review File

Supplementary Table 1 Probe usage for meningioma subclassifation.

Supplementary Table 2 Multiplexed ion beam imaging antibody information.

## Data Availability

The snRNA sequencing and spatial transcriptomic data have been deposited to the GEO database (https://www.ncbi.nlm.nih.gov/geo/) with the accession codes GSE313693 and GSE313694. Processed data files have been deposited to Zenodo (10.5281/zenodo.17296825). To adhere to local regulations, DNA methylation and clinical outcome data can be shared after completion of a data transfer agreement for noncommercial use. Requests should be made to the corresponding author(s), with responses expected within 14 days of receipt of request. To identify cell-type-distinguishing probes, we obtained purified cell-type-specific methylation data from the GEO. Datasets included were GSE110554 (ref. ^65^) (B cells, CD4^+^ and CD8^+^ T cells, monocytes and natural killer cells), GSE112179 (ref. ^66^) (neurons), GSE122126 (ref. ^67^) (blood endothelium and neurons), GSE140295 (ref. ^68^) (blood endothelium), GSE167998 (ref. ^65^) (B cells, CD4^+^ and CD8^+^ T cells, eosinophils, monocytes, natural killer cells, neutrophils and regulatory T cells), GSE184269 (ref. ^69^) (B cells, CD4^+^ and CD8^+^ T cells, monocytes and natural killer cells), GSE191200 (ref. ^70^) (microglia), GSE63409 (ref. ^71^) (blood cell progenitors), GSE74877 (ref. ^72^) (endothelium and fibroblasts), GSE82234 (ref. ^73^) (blood endothelium), GSE84395 (ref. ^74^) (blood endothelium), and GSE88824 (ref. ^75^) (B cells, CD4^+^ and CD8^+^ T cells, monocytes, natural killer cells and neutrophils).

## Code availability

All R packages, including version numbers, are listed in Methods. The NePSTA implementation can be accessed from GitHub at https://github.com/heilandd/NePSTA. The uwot package used can be access from GitHub at https://github.com/jlmelville/uwot. The SPATA2 tool can be accessed from GitHub at https://github.com/theMILOlab/SPATA2. The Toffy tool for image extraction is available at https://github.com/angelolab/toffy. Code for the implementations and additional subfigure generations are platform and infrastructure specific but available from the corresponding authors for noncommercial use.
