# Supplementary material for: A microenvironment-determined risk continuum refines subtyping in meningioma and reveals determinants of machine learning-based tumor classification
Source: Nat Genet. 2026 Feb 9;58(2):341–54. doi: 10.1038/s41588-025-02475-w (PMC12900647; doi:10.1038/s41588-025-02475-w)
Supplement: Supplementary file 1 — Supplementary Figures 1–3, The German “Aggressive Meningiomas” Consortium (KAM) members. [file 41588_2025_2475_MOESM1_ESM.pdf]

nature genetics

Supplementary information https://doi.org/10.1038/s41588-025-02475-w

# A microenvironment-determined risk continuum refines subtyping in meningioma and reveals determinants of machine learning-based tumor classification

In the format provided by the authors and unedited

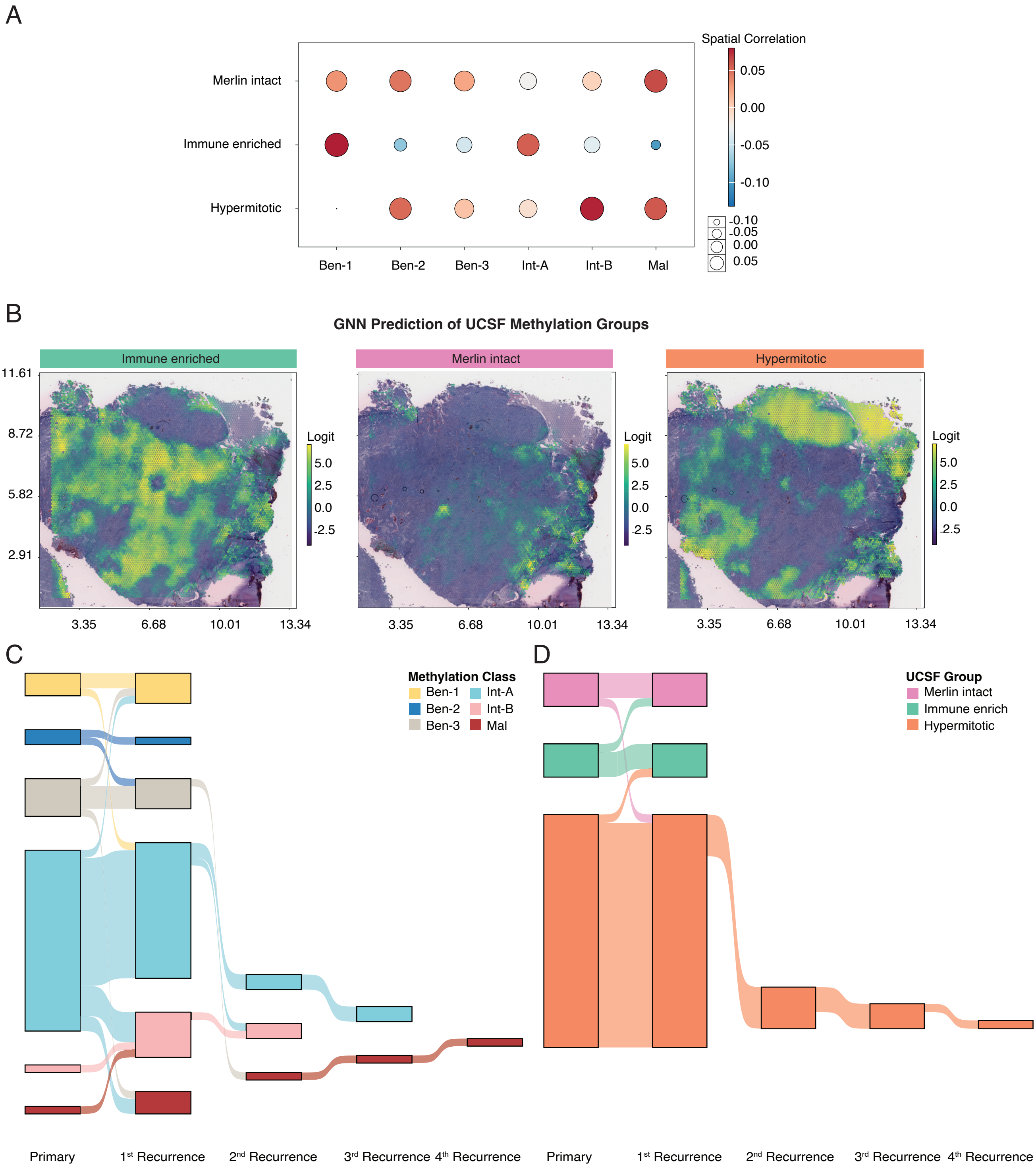

A
Spatial Correlation
0.05
0.00
-0.05
-0.10
-0.10
-0.05
0.00
0.05
Merlin intact
Immune enriched
Hypermitotic
Ben-1
Ben-2
Ben-3
Int-A
Int-B
Mal
B
GNN Prediction of UCSF Methylation Groups
Immune enriched
Merlin intact
Hypermitotic
11.61
8.72
5.82
2.91
Logit
5.0
2.5
0.0
-2.5
3.35
6.68
10.01
13.34
C
Methylation Class
Ben-1
Int-A
Ben-2
Int-B
Ben-3
Mal
Primary
1st Recurrence
2nd Recurrence
3rd Recurrence
4th Recurrence
D
UCSF Group
Merlin intact
Immune enrich
Hypermitotic
Primary
1st Recurrence
2nd Recurrence
3rd Recurrence
4th Recurrence

**Supplementary Figure 1: Spatial correlation between methylation classes and methylation groups and epigenetic switching over primary and recurrent cases** Spatial correlation plot between methylation classes (MC) MC ben-1, ben-2, ben-3, int-A, int-B and Mal and methylation groups (MGs) Immune enriched, Merlin intact and Hypermitotic. (A). Surface heatmaps demonstrate the subclass logit (logarithm of the odds) for the different MGs within the example case (B). A total of 36 cases with multiple resections were identified of which MC-switching occurred for multiple *NF2*-mutant MCs, but not for *NF2*-wildtype ben-2, again indicating that *NF2*-mutant MCs are a spectrum rather than definitive subgroups (C). Annotation of the same 36 cases using the MG annotation, identifies less pronounced switching as is expected for a 3-group versus a 6-group system (D).

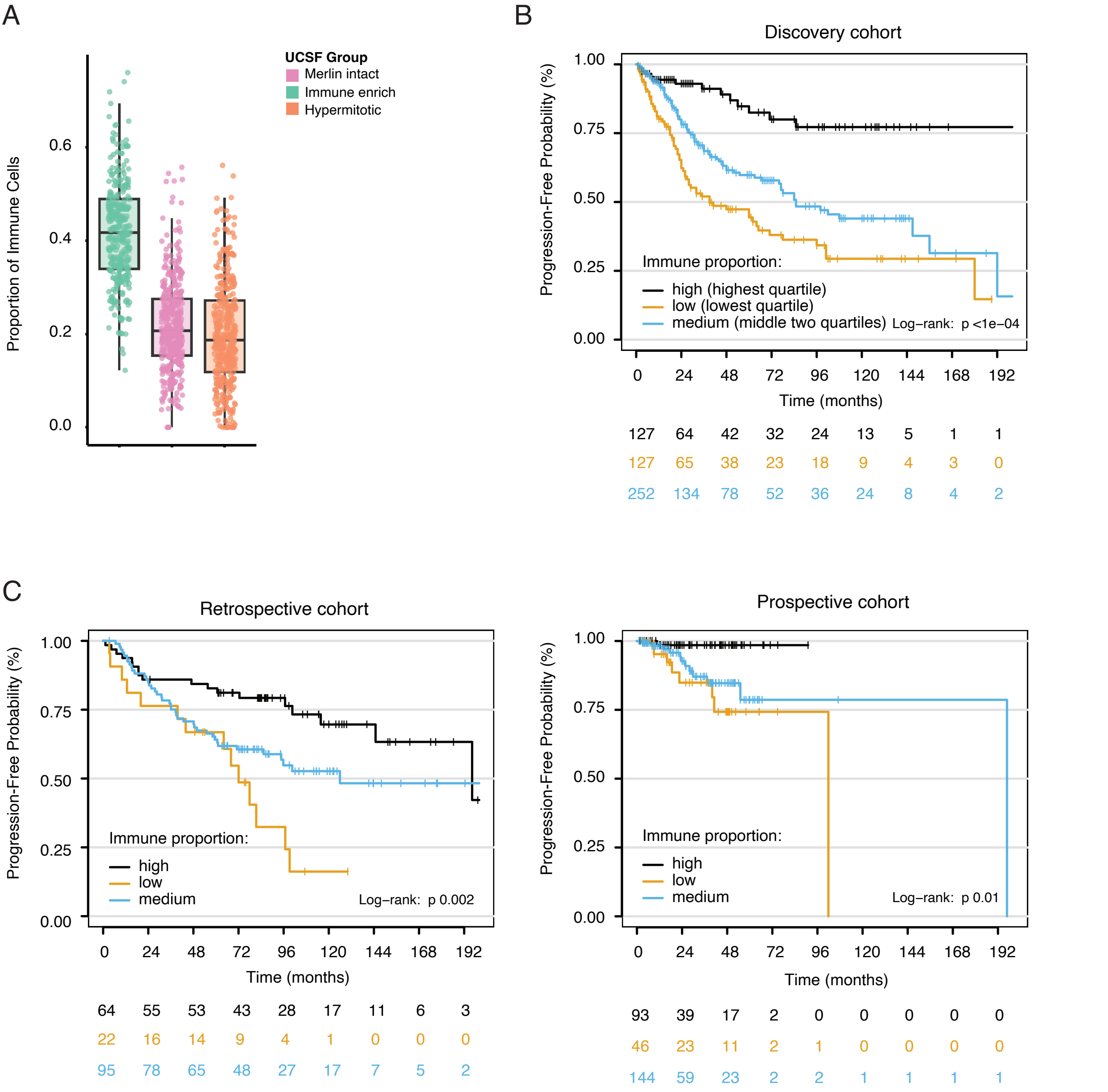
A
UCSF Group
Merlin intact
Immune enrich
Hypermitotic
Proportion of Immune Cells
0.0
0.2
0.4
0.6
B
Discovery cohort
Progression-Free Probability (%)
1.00
0.75
0.50
0.25
0.00
Immune proportion:
high (highest quartile)
low (lowest quartile)
medium (middle two quartiles)
Log−rank: p <1e−04
0 24 48 72 96 120 144 168 192
Time (months)
127 64 42 32 24 13 5 1 1
127 65 38 23 18 9 4 3 0
252 134 78 52 36 24 8 4 2
C
Retrospective cohort
Progression-Free Probability (%)
1.00
0.75
0.50
0.25
0.00
Immune proportion:
high
low
medium
Log−rank: p 0.002
0 24 48 72 96 120 144 168 192
Time (months)
64 55 53 43 28 17 11 6 3
22 16 14 9 4 1 0 0 0
95 78 65 48 27 17 7 5 2
Prospective cohort
Progression-Free Probability (%)
1.00
0.75
0.50
0.25
0.00
Immune proportion:
high
low
medium
Log−rank: p 0.01
0 24 48 72 96 120 144 168 192
Time (months)
93 39 17 2 0 0 0 0 0
46 23 11 2 1 0 0 0 0
144 59 23 2 2 1 1 1 1

**Supplementary Figure 2: Kaplan-Meier analysis of progression-free survival for cases stratified by the quartile distribution epigenetic deconvoluted immune percentages**

The number of immune cells as determined by methylation deconvolution is the highest for the immune-enriched methylation group. Immune enriched n=264, Merlin intact n=374 and Hypermitotic n=332. (A). Kaplan-Meier analysis of progression-free survival for cases stratified by the quartile distribution of immune cell presence into a high (top quartile), intermediate (middle two quartiles) or low (bottom quartile) resulted in a significantly different outcome in the discovery (log-rank test, $chi^2$ test statistic = 35.4, 1 DoF) (B), retrospective validation (log-rank test, $chi^2$ test statistic = 12.9, 1 DoF) (C, left-panel) and prospective validation cohorts (log-rank test, $chi^2$ test statistic = 8.7, 1 DoF) (C, right-panel). Boxplots represent the upper and lower quartiles, while whiskers reach to the most outlying samples that are within 1.5 interquartile ranges (IQR) from the quartiles. Median values are labelled as horizontal lines in the boxes.

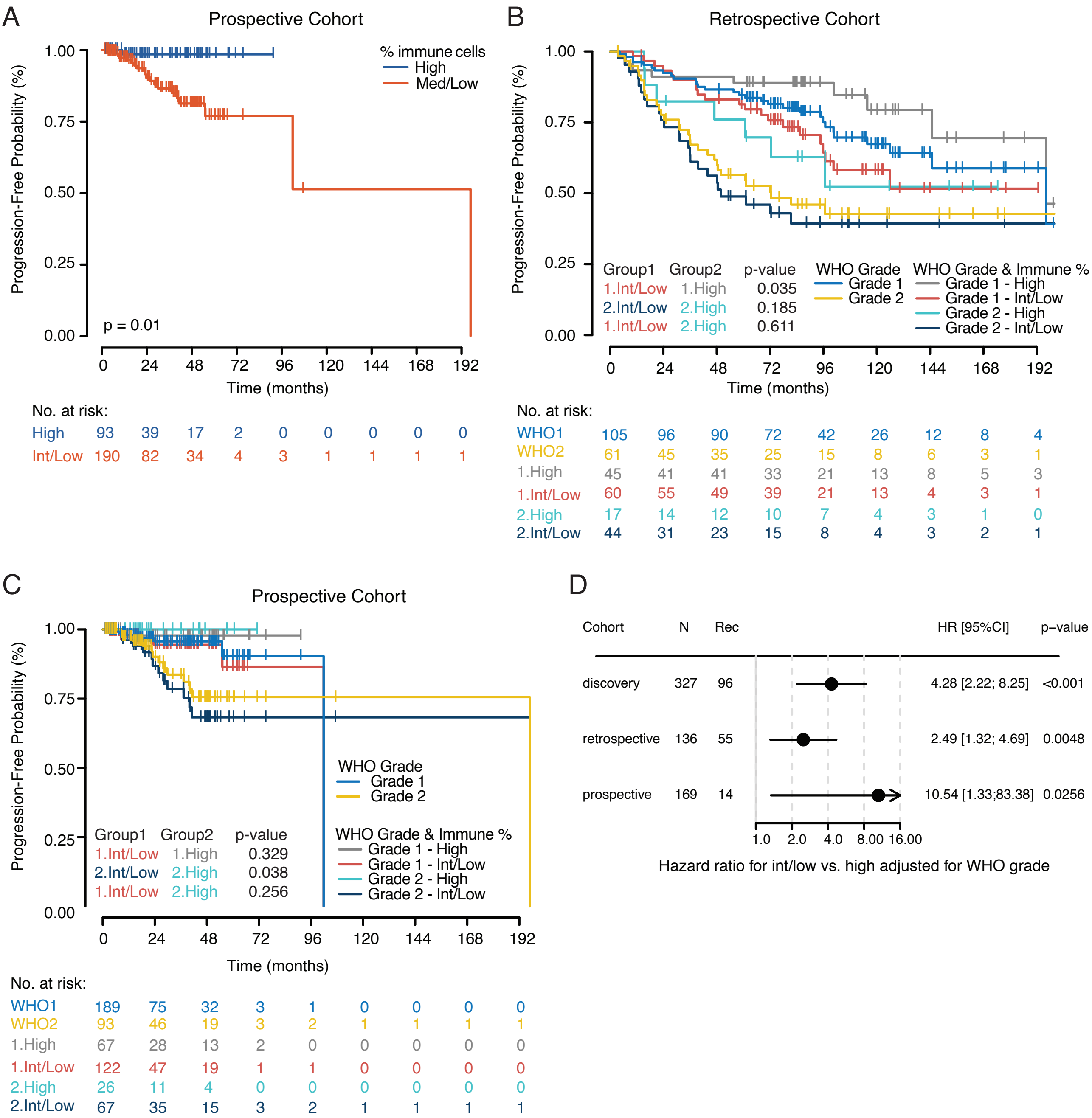
A
Prospective Cohort
% immune cells
High
Med/Low
p = 0.01
Progression-Free Probability (%)
1.00
0.75
0.50
0.25
0.00
0 24 48 72 96 120 144 168 192
Time (months)
No. at risk:
High 93 39 17 2 0 0 0 0 0
Int/Low 190 82 34 4 3 1 1 1 1
B
Retrospective Cohort
Group1 Group2 p-value
1.Int/Low 1.High 0.035
2.Int/Low 2.High 0.185
1.Int/Low 2.High 0.611
WHO Grade
Grade 1
Grade 2
WHO Grade & Immune %
Grade 1 - High
Grade 1 - Int/Low
Grade 2 - High
Grade 2 - Int/Low
Progression-Free Probability (%)
1.00
0.75
0.50
0.25
0.00
0 24 48 72 96 120 144 168 192
Time (months)
No. at risk:
WHO1 105 96 90 72 42 26 12 8 4
WHO2 61 45 35 25 15 8 6 3 1
1.High 45 41 41 33 21 13 8 5 3
1.Int/Low 60 55 49 39 21 13 4 3 1
2.High 17 14 12 10 7 4 3 1 0
2.Int/Low 44 31 23 15 8 4 3 2 1
C
Prospective Cohort
WHO Grade
Grade 1
Grade 2
Group1 Group2 p-value
1.Int/Low 1.High 0.329
2.Int/Low 2.High 0.038
1.Int/Low 2.High 0.256
WHO Grade & Immune %
Grade 1 - High
Grade 1 - Int/Low
Grade 2 - High
Grade 2 - Int/Low
Progression-Free Probability (%)
1.00
0.75
0.50
0.25
0.00
0 24 48 72 96 120 144 168 192
Time (months)
No. at risk:
WHO1 189 75 32 3 1 0 0 0 0
WHO2 93 46 19 3 2 1 1 1 1
1.High 67 28 13 2 0 0 0 0 0
1.Int/Low 122 47 19 1 1 0 0 0 0
2.High 26 11 4 0 0 0 0 0 0
2.Int/Low 67 35 15 3 2 1 1 1 1
D
Cohort N Rec HR [95%CI] p−value
discovery 327 96 4.28 [2.22; 8.25] <0.001
retrospective 136 55 2.49 [1.32; 4.69] 0.0048
prospective 169 14 10.54 [1.33;83.38] 0.0256
1.0 2.0 4.0 8.00 16.00
Hazard ratio for int/low vs. high adjusted for WHO grade

**Supplementary Figure 3: Kaplan-Meier analysis of progression-free survival for cases stratified by high versus intermediate/low number of immune cells**

Kaplan-Meier analysis of progression-free survival for cases stratified by the quartile distribution of immune cell presence into a high (top quartile), or low (bottom)/intermediate (middle two quartiles) resulted in a significantly different outcome in the prospective validation cohort (log-rank test, $chi^2$ test statistic=6.7, 1 DoF) (A). Kaplan-Meier analysis of progression-free survival for WHO grade 1 or 2 cases, or WHO grade 1 or 2 cases split based on the number of immune cells in the retrospective (B) and prospective (C) validation cohorts. Log-rank tests, all with 1 DoF. $Chi^2$ test statistic for retrospective cohort 1.Int/low vs. 1.High 4.4, 2.Int/low vs. 2.High 1.8 and 1.Int/low vs. 2.High 0.3. For the prospective cohort 1.Int/low vs. 1.High 1.0, 2.Int/low vs. 2.High 4.3 and 1.Int/low vs. 2.High 1.3. Forest plot indicating that the immune cell component is a significant predictor after correction for the WHO grade in all 3 cohorts excluding *NF2*-wildtype MC ben-2 cases (F).

## The German „Aggressive Meningiomas” Consortium (KAM) members

Christel Herold-Mende

- Department of Neurosurgery, University Hospital Heidelberg, Heidelberg, Germany

Rolf Warta

- Department of Neurosurgery, University Hospital Heidelberg, Heidelberg, Germany

Wolfgang Wick

- Department of Neurology and Neurooncology Program, National Center for Tumor Diseases, Heidelberg University & German Cancer Research Center (DKFZ), Heidelberg, Germany

Guido Reifenberger

- Institute of Neuropathology, Heinrich Heine University, Medical Faculty, and University Hospital Düsseldorf, Düsseldorf, Germany

Marc Remke

- Department of Pediatric Hematology and Oncology, University Medical Center of Saarland, Saarland University, Homburg/Saar, Germany.

Katrin Lamszus

- Department of Neurosurgery, University Medical Center Hamburg-Eppendorf, Hamburg, Germany

Franz Ricklefs

- Department of Neurosurgery, University Medical Center Hamburg-Eppendorf, Hamburg, Germany.

Christian Mawrin

- Department of Neuropathology, University Hospital Magdeburg, Germany

Jörg-Christian Tonn

- Department of Neurosurgery, Ludwig-Maximilians-University Munich, 81377 Munich, Germany.

Christian Schichor

- Department of Neurosurgery, LMU University Hospital, LMU Munich, Munich, Germany.
- German Cancer Consortium (DKTK), Partner Site Munich, Munich, Germany.

Christine Jungk

- Department of Neurosurgery, University Hospital Heidelberg, Heidelberg, Germany.

Matthias Schlesner
- Biomedical Informatics, Data Mining and Data Analytics, Faculty of Applied Computer Science and Medical Faculty, University of Augsburg, Augsburg, Germany

Gabriele Schackert
- Department of Neurosurgery, Faculty of Medicine and University Hospital Carl Gustav Carus, Technische Universität Dresden, Dresden, Germany

Christian Schichor
- Department of Neurosurgery, LMU University Hospital, LMU Munich, Munich, Germany

Ortrud Uckermann
- Department of Neurosurgery, Faculty of Medicine and University Hospital Carl Gustav Carus, Technische Universität Dresden, Dresden, Germany

Matthias Kirsch
- Department of Neurosurgery, Asklepios Kliniken Schildautal Seesen, Seesen, Germany

Catharina Lotsch
- Department of Neurosurgery, University Hospital Heidelberg, Heidelberg, Germany

Kerim Beseoglu
- Department of Neurosurgery, Heinrich Heine University, Medical Faculty, and University Hospital Düsseldorf, Düsseldorf, Germany

Bastian Malzkorn
- Institute of Neuropathology, Heinrich Heine University, Medical Faculty, and University Hospital Düsseldorf, Düsseldorf, Germany

Michael Schmutzer
- Department of Neurosurgery, LMU University Hospital, LMU Munich, Munich, Germany

Tareq Juratli
- Department of Neurosurgery, Faculty of Medicine and University Hospital Carl Gustav Carus, Technische Universität Dresden, Dresden, Germany

Felix Sahm
- Department of Neuropathology, University Hospital Heidelberg, Heidelberg, Germany
- Clinical Cooperation Unit Neuropathology, German Cancer Research Centre (DKFZ), German Consortium for Translational Cancer Research (DKTK), Heidelberg, Germany
