# Supplementary material for: A microenvironment-determined risk continuum refines subtyping in meningioma and reveals determinants of machine learning-based tumor classification
Source: Nat Genet. 2026 Feb 9;58(2):341–54. doi: 10.1038/s41588-025-02475-w (PMC12900647; doi:10.1038/s41588-025-02475-w)
Supplement: Supplementary file 3 — Probe usage for meningioma subclassifation. [file 41588_2025_2475_MOESM3_ESM.pdf]

nature portfolio

Peer Review File

A microenvironment-determined risk continuum refines subtyping in meningioma and reveals determinants of machine learning-based tumour classification

Corresponding Author: Professor Felix Sahm

Version 0:

Decision Letter:

31st Jan 2025

Dear Professor Sahm,

First, I am so sorry that it has taken so long to get this decision to you. Thank you for bearing with me.

Your Article, "A microenvironment-determined risk continuum replaces subtyping in meningioma and reveals determinants of machine learning-based tumour classification" has now been seen by 3 referees. You will see from their comments below that while they find your work of interest, some important points are raised. We are interested in the possibility of publishing your study in Nature Genetics, but would like to consider your response to these concerns in the form of a revised manuscript before we make a final decision on publication.

We therefore invite you to revise your manuscript taking into account all reviewer and editor comments. Please highlight all changes in the manuscript text file. At this stage we will need you to upload a copy of the manuscript in MS Word .docx or similar editable format.

We are committed to providing a fair and constructive peer-review process. Do not hesitate to contact us if there are specific requests from the reviewers that you believe are technically impossible or unlikely to yield a meaningful outcome.

When revising your manuscript:

*1) Include a “Response to referees” document detailing, point-by-point, how you addressed each referee comment. If no action was taken to address a point, you must provide a compelling argument. This response will be sent back to the referees along with the revised manuscript.

*2) If you have not done so already please begin to revise your manuscript so that it conforms to our Article format instructions, available
here.
Refer also to any guidelines provided in this letter.

*3) Include a revised version of any required Reporting Summary: https://www.nature.com/documents/nr-reporting-summary.pdf
It will be available to referees (and, potentially, statisticians) to aid in their evaluation if the manuscript goes back for peer review.
A revised checklist is essential for re-review of the paper.

Please be aware of our guidelines on digital image standards.

EXTENDED DATA FIGURES

When re-submitting your manuscript, please ensure that any supplementary figures and tables that are crucial to the manuscript’s conclusions are converted into Extended Data figures and tables to increase visibility of these data. Extended Data figures and tables are online-only (present in the online PDF and full-text HTML versions of the paper), peer-reviewed

display items that provide essential background to the article but are not included in the main article due to space constraints. A maximum of ten Extended Data display items (figures and tables) is permitted.

Please use the link below to submit your revised manuscript and related files:

Link Redacted

Note: This URL links to your confidential home page and associated information about manuscripts you may have submitted, or that you are reviewing for us. If you wish to forward this email to co-authors, please delete the link to your homepage.

We hope to receive your revised manuscript within four to eight weeks. If you cannot send it within this time, please let us know.

Please do not hesitate to contact me if you have any questions or would like to discuss these revisions further.

Nature Genetics is committed to improving transparency in authorship. As part of our efforts in this direction, we are now requesting that all authors identified as ‘corresponding author’ on published papers create and link their Open Researcher and Contributor Identifier (ORCID) with their account on the Manuscript Tracking System (MTS), prior to acceptance. ORCID helps the scientific community achieve unambiguous attribution of all scholarly contributions. You can create and link your ORCID from the home page of the MTS by clicking on ‘Modify my Springer Nature account’. For more information please visit please visit www.springernature.com/orcid.

We look forward to seeing the revised manuscript and thank you for the opportunity to review your work.

Sincerely,

Safia Danovi, PhD
Senior Editor, Nature Genetics
ORCiD: 0009-0007-7822-5479

Referee expertise:

Referee #1: genomics, meningioma

Referee #2: neuropathology, epigenetics

Referee #3: computational, brain tumours, TME

Reviewers' Comments:

Reviewer #1 (Remarks to the Author):

The authors of the manuscript entitled “A microenvironment-determined risk continuum replaces subtyping in meningioma and reveals determinants of machine learning-based tumour classification” aim to provide a new tumor microenvironment-based meningioma risk assessment. To do so, they use a total of 6876 meningioma samples and performed multiple experimental approaches such as snRNA-seq and bulk DNA methylation along with extensive computational and machine learning approaches to arrive at their conclusions. By the end of the manuscript, I was convinced by the authors that meningiomas display a continuum rather than discrete groups/subgroups and techniques such as DNA methylation or RNA sequencing only provide a static snapshot of what is actually going on inside the tumor. However, this does not make the previously reported groups/subgroups invalid in classifying meningiomas (e.g. one does not “replace” the other) but rather prompts the use of more sensitive approaches to interrogate meningiomas at a single cell level, which highlights the underlying tumor heterogeneity that may easily be missed by bulk approaches. Despite this rhetorical faux pas, the overall study design is exceptional, and the conclusions are otherwise well supported by the data. This study will provide a significant conceptual advance in our understanding of the fundamental biology of the most common brain tumor, and I hope to have the opportunity to review a revised version of this manuscript.

Major suggestions
1. The 3-hop subgraph analysis of spatial transcriptomic data is thoughtful and interesting, but more details on the development and validation of this classifier that transforms meningioma MCs onto spatial transcriptomes is needed. If the authors did not split their “43 slides” (Is that 43 separate tumors? Or 43 sections from one tumor? Later in the paper, the authors state “43 cases”… what does this mean?) into discovery and validation cohorts, they are encouraged to use the data from Lucas et al. Nature Genetics 2024 to validate their classifier and report only the precision, accuracy, recall, and F1 scores from independent validation cohort (not discovery cohort) samples.
2. The authors appropriately note in the Introduction that previously published meningioma molecular classifiers have identified groups of tumors that are defined by immune signatures and immune cells (Choudhury et al. and Nassiri et al. in

Figure 1A), but almost all of the analyses in their figures are limited to a molecular classifier (Sahm et al. Lancet Oncology 2017) that was not initially built around the observation that immune cells may influence the molecular classification of meningiomas. The breakdown of microenvironment signatures/cells and neoplastic signatures/cells from their various analyses (single-nuclei, spatial, imaging, deconvolution, etc) should at the very least also be shown across molecular groups of meningiomas (Choudhury et al., as the authors have done in Figure 5). Analysis across meningioma subgroups is not necessary, as subgroups of meningiomas are not known to be distinguished by microenvironment composition.
3. The analyses in Supplementary Figure 2 were interested and appreciated, but CD81 and SPP1 feel cherry-picked. Could the authors take all of the CpG probes mapping to differentially expressed genes in Supplementary Figures 1C and 1D and look at the methylation and expression of these genes, as has been done for CD81 and SPP1 in Supplementary Figure 2?
4. The authors make quantitative conclusions from the UMAP visualization in Figure 3C without showing a quantitative analysis (e.g. the reader is expected to rely on UMAP gestalt). The authors should calculate the proximity of each meningioma to other meningiomas with the same DNA methylation class vs the proximity of each meningioma to other meningiomas with different DNA methylation classes using all signatures vs neoplastic signatures vs stromal signatures. See Liu et al. Genome Biology 2024 for a similar analysis. The same analysis is suggested for Figure 4A.
5. It is important to understand if the CNAs from Figure 2B (that come up again in Figures 3 and 4) segregate in single neoplastic nuclei from Figure 2A as would be expected across MCs (Sahm et al.) or DNA methylation groups (Choudhury et al.). The pipelines used for development of these two molecular classifiers were inherently different, with Choudhury et al. using a pipeline that controlled for biases from CNAs for clustering. It would be good to know which classification system provides better segregation of CNAs in cells derived from tumors of different molecular groups.
6. The last paragraph of the Results that is associated with Figure 4, where the authors assert that their data solve "several current controversies and uncertainties in meningioma research at once", is highly speculative and should be moved to the Discussion. (1) The absence of similar analyses in ependymomas or medulloblastomas, (2) the finding that glioblastomas exist along a dynamic and interchangeable molecular continuum, and (3) the absence of mechanistic and functional studies to test these hypotheses need to be specifically mentioned in the Discussion.
7. The stratification of meningioma clinical outcomes in the retrospective and prospective validation cohorts in Supplementary Figure 7 is not great, and only PFS is shown in all KM curves throughout the paper. This limitation needs to be appropriately mentioned in the Results and in the Discussion, and OS data need to be provided adjacent to all KM curves.
8. The stratification of KM curves (for PFS and OS) in Figure 6C with the addition of PU.1 IHC to WHO grade is impressive, particularly as previous attempts to distil meningioma molecular classification systems down to IHC markers (Nassiri et al) has failed to validate (Naslund et al. Brain and Spine 2023). The authors should incorporate these references into their Discussion alongside explanations for why they think their IHC approach is superior and will not be similarly invalidated in subsequent studies.

Minor critiques
1. To say that the microenvironment-determined risk continuum reported here "replaces" the well-established molecular groups of meningiomas (in the title and elsewhere in the manuscript) is an overstatement. I strongly suggest the use of "refines" instead of "replaces" throughout.
2. The focused Introduction is very much appreciated by this meningioma-zealot of a reviewer… but for a broad scientific audience, it is suggested that the authors perhaps contextualize their study (and foreshadow their results) in broader terms. The findings of this study have broad, wide-reaching implications for cancer classification, but as written the Introduction gives the impression that this study is narrow in scope and impact (which it is not).
3. In Figure 1A, it is suggested that Choudhury and Chen et al. Neuro-Oncology 2022 be referenced next to the subdivision of Hypermitotic meningiomas into Hypermetabolic and Proliferative meningiomas. It is confusing that this figure (and the paper more broadly) omits reference to the finding of 2 molecular groups of meningioma from Olar et al. Acta Neuropathology 2017. If this system was found to be not robust or was not validated in subsequent studies, this shortcoming should be mentioned and the authors are encouraged to do so in the Discussion of their manuscript.
4. Do I misinterpret Figure 1B, or is the sex of three patients shaded as neither Female nor Male? The authors are encouraged to also shade these samples according to the DNA methylation groups from Choudhury et al. and DNA methylation subgroups from Nassiri et al. in Figure 1A.
5. Could the clustering in Figure 2C be due to batch effects? Why wasn't Seurrat integration algorithm and/or harmony batch correction performed.
6. Methods to calculate cell cluster abundance (Figures 2D, 2E, 2G) across samples/groups is not present. It is not clear if these data have been normalized to the total cells captured in a particular sample. I understand orthogonal validations have been performed but if this analysis is present in the manuscript, it's methodology and results should be clear.
7. For some of the samples, the % TAM is as high as 90%. How can this be? What are the histological/morphological features of such tumors? Are these tumors regressing and not growing at all?
8. Figure 2J – It's not clear what is represented by the size of the circle.
9. While UMAP is a good tool for visualizing high dimensional data, it should ideally not be used for identifying clusters in the data. I would like to see unsupervised cluster identification using a bonafide clustering algorithm such as kNN or k-means etc. and whether it aligns with what is shown in Figures 3C and 4A.
10. "On top of the evidence for a striking difference in TAM abundance between low-risk and high-risk NF2 mutant meningiomas, these results suggest that differentiation and function of those cells change with malignancy." "Yet, it remains elusive what functional and cellular implications the CpGs relevant for classification confer." Correlation is not causation, and the authors are encouraged to revise these statements (and ones like them) in the absence of mechanistic and functional experiments to appropriately test their stated hypotheses.
11. Figure 4D and 4E – Does this point towards the possibility of differential immune cell recruitment and/or evasion capabilities by different tumor subtypes within the same tumor? Or differential tumor evolution at different sites within the same tumor?

12. What are the red and green cells in Figure 6F?

Reviewer #2 (Remarks to the Author):

In this paper by Mass and co-authors, multi-omic profiling and multiple lines
of orthogonal computational evaluation of a large cohort of meningioma samples, led to the identification and characterisarion of changes in the tumour microenvironment
which contributed to the epigenetic classification of the tumours and enabled prediction of patients outcome. They also provide evidence to support the interpretation that a TME-determined risk continuum is potentially more informative in terms of prognosis assessment than classifying meningiomas into distinct subgroups.

This is an impressive work, the approach is original, the methodology is state of the art, the results are very well presented. The patients cohorts used are impressively large and this ensures the conclusion of the paper are strong.

Overall, the conclusion that the proposed changes to TAM (epigenetics and IHC staining) and the evidence that intratumour heterogenity can lead to the misdiagnosis of patients is a very valuable contribution to the field. At times the authors overstate the novelty or unexpectedness of their findings - for instance I don't think anyone would be surprised that the non-tumour cells are contributing to the bulk Heidelberg classification. Having said that, the paper addresses an important diagnostic issue in the field and has the potential to significantly contribute to a better stratification of meningiomas, which is a significant medical issue, ie not been able to predict which grade 1 tumours will recur and behave more aggressively.

The spatial analysis and teasing apart what is causing the classifier diagnosis is not for me to assess as it does not fall within my field of expertise but all seem reasonable.

Other points

What is driving the large difference in Int-B macrophages from all other macrophages in Fig1A? I might have thought this would result in a unique immune signature for this MC subtype from their deconvolution analysis?

Microglia number changes are more striking than those of the macrophages (Fig.1J), is this biologically relevant?

The analysis of the TAM/MG should be developed further, comparing and contrasting how they behave in the different MC subclasses - perhaps they could use the spatial data to do some receptor ligand analysis etc? This is essential to fully capitalise on the approach in terms of novelty of the findings.

There are a lot of Kaplan Meyers plots without significance values.

Reviewer #3 (Remarks to the Author):

A. Summary of the results

The current state of the art approach to classification of meningiomas (like many other brain tumours) is based on molecular profiling of bulk tumour tissue samples, particularly employing machine learning classification of DNA methylation array data.

However, discrepancies arise across different classifiers (and the established WHO grades) particularly in the case of NF2 mutated tumours, whereas NF2 wild type tumours are generally consistently classified.

This paper investigates the hypothesis that NF2 mutant meningiomas present molecularly as a continuum, rather than neatly defined subtypes. They propose that this contiuum is strongly affected by the abundance and functional behaviour of non-neoplastic cells, particularly tumour associated macrophages (TAMs). They observe that the proportion of TAMs is low in NF2 wild type tumours, and in NF2 mutant tumours that proportion of TAMs decreases with higher grades (which is interestingly opposite to what is seen in other brain tumours). Also, they observe that the composition of TAMs changes with tumour grade. These differences can be used for better diagnostics. While tumour molecular profiling, especially multi-modal, yields the most precise results, the paper also shows that relatively simple tissue staining for the TAM-related marker PU.1 can also be used to discriminate grades, and therefore suggests a fairly cost-effective method of classification.

B. Originality and significance

This study builds and elaborates on previous results from the authors relating to the development of precise methylation-based machine learning classifier for meningiomas. While previous work has shown the efficacy of those methods, the biological explanation of the results has remained elusive. The significance of this new paper is in providing a better understanding of what molecular/cellular factors contribute to differences in proposed meningioma classes. In particular this paper greatly advances our understanding of the important role played by the TME, particularly TAMs, in identifying markers of meningioma progression. Previously this has been difficult to understand, particularly with regards to NF2 mutant tumours. I believe the results of this paper are original and highly significant. As noted in my later remarks about the method, the

paper is also very thorough in its investigation of the overall hypothesis, employing several complementary approaches to derive its conclusions.

C. Data and methodology

There are a lot of parts in this paper, making it difficult to fully comprehend on an initial reading. According to my count there are at least 10 different experiments described in the results, and at times it can be difficult to follow how they all fit together. Indeed it took me a very long time to review the paper because I had to carefully re-read it several times over, taking many notes, to make sure I understood the methodology. I do not think that this is a problem with the methods, rather their presentation, and perhaps the need to squeeze it all into one manuscript. I think the presentation would be greatly improved with more "sign posts" for the reader, and perhaps a summary figure that ties all the experimentation together. Related to this point, given the very large number of experiments described there is a correspondingly large number of data sets used, and it can be very difficult for the reader to keep track of all them and how they relate to each other. A good example of this is that the abstract claims "evaluation in 6,876 meningioma samples": while this may be true in aggregate (and I found it hard to check), the large number of samples mentioned are spread across many different experiments, and I think it is not accurate to present the study in this way. This complexity is also apparent in the very large number of sub-plots within each of the main result figures, especially Figure 2.

Below I briefly summarise each of the main parts in the methods:

- snRNA applied on 26 samples, 44,266 cells, subjected to unsupervised clustering. CD68 expression, TAM cluster enriched for WHO grade1, also TAM cells from higher grade tumours cluster together. TAM abundance is low in NF2 wildtype and TAM abundance decreases with grade for NF2 mutants. Opposite of what is seen in other brain tumours.
- the above findings were validated by CD68 IHC on 120 meningiomas, plus mutliplexed ion beam imaging on WHO grade 2 and 3, finding that the composition of TAMS differs between stages
- spatial transcriptomics was applied to 43 slides, trained a GAN to predict MC based on local TME. This part is not clearly explained in the paper, and its significance is not clear. Accuracy etc is high, but it is not apparent whether that is just overfitting the data
- Single-cell deconvolution of each microenvironment subgraph, and subgraphs from individial samples were stratified by risk. Showed reduced microglia-like cells and increased TAMS
- Investigated correlation between CD68 protein expression and methylation probes in 120 meningiomas when subjected to Brain Tumour Classifier (random forest algorithm), and looked for probes that are significant to classification. Found that probes used to pick ben-1 from other classes were correlated to CD68 expression. Maybe CpGs hypomethylated in TAMs underlie the separation. Then Considered RNA expression of genes related to the probes that were either related to CD68-hypomethylation or specific to MC ben-1 classification. Found increased expression of these genes in the TAM population. Thus the random forest classifier is likely to be picking probes that are related to genes with increased expression in TAMs, especially in MC ben-1.
Factorisation (NMF) of 4502 bulk methylomes, resulting "signatures" were matched against methylation profiles from published isolated cell types, yielding 6 neoplastic singatures and 7 stromal signatures. UMAP clustering showed that MC groups clusterd more strongly when stromal signatures were used, compared to neoplastic signatures only. Characteristic CNV signatures were then investigated against the NMF-defined signatures. It was found that typical risk-related CNV patterns could be identified with different NMF-signatures. Whereas NF2-wildtype have a homogeneous profile of a small number of stromal and neoplastic signatures, the NF2-mutant feature more complex mixtures.
- analysis of sub-clonal heterogeneity. Spatially resolved transcriptomic data from 43 cases, inferCNV + hierarchical clustering, used the GAN model to predict MC assignment based on spatial archetecture and cell type composition. In 16 cases with 38 different clines, nearly half had heterogeneous spatial distribution of MCs, although MC mal was more homogeneous in malignant samples.
- Investigated stability of MCs in recurrent meningiomas showed significant switching of MCs for NF2-mutants, suggesting heterogeneity in molecular states within tumours
- Assessed bulk methylation from three clinical cohorts (sizes not mentioned in the main text). Each case annotated for immune cell enrighment. It found that high immune cell enrichment was identified for WHO grade1 and MC ben-1 cases. Stratification into low, medium and high immune cell proportion was associated with differences in clinical courses. Findings also showed that immune cell proportion could be descriminatory between WHO grades 1 and 2
- Lastly immune cell proportion calculation was investigated using tissue staining for PU.1. Showing that standard (and cheaper) lab techniques can be used to estimate TAMs, which can then be used to inform diagnostics without molecular profiling

D. Appropriate use of statistics and treatment of uncertainties

The statistical and bioinformatics methods applied in the paper are appropriate and, for the most part, thoroughly described in the supplementary materials.

The description of the GAN for classifying spatial transcriptomics on 43 slides was probably overkill for the main body of the text, and overall will probably be difficult for the target audience to follow. Excellent precision/recall results were obtained, but some questions remain about the capacity for generalisation, given the relatively limited amount of data used in training and validation.

Unfortunately the main software employed in the paper is only available upon request from the authors so it is not possible to assess its overall quality or validity, and we must therefore take the authors on trust.

E. Conclusions: robustness, validity, reliability

While there is undoubted complexity in the experimental methods (and sheer number of them) in this paper, I cannot fault the overall conclusions drawn. While the authors briefly allow themselves to speculate about the mechanisms behind the observed phenomenon, they are fairly circumspect in this regard, and I also believe it is best to be cautious at this point. Further study may be required, now that a strong hypothesis has been established. Although some readers may have preferred additional exploration into why meningiomas are apparently so different to other brain tumours in this regard.

F. Suggested improvements: experiments, data for possible revision

There are 2 main methodological points requiring a response from the authors:

1. a major issue is that the study makes a big point of macrophage numbers, distinguishing disease progression states. In the figures/graphs "immune cell" data is shown without explaining which immune cell types -are they measuring all immune cell types or only myeloid cells: macrophages /microglia only. Authors should clarify.

2. In terms of mechanisms relating to myeloid cell infiltration - can the authors comment on whether the myeloid cells exhibit immunosuppressive or inflammatory phenotypes & changes i these states during disease progression?

References: appropriate credit to previous work?

The references are appropriate and up to date.

H. Clarity and context: lucidity of abstract/summary, appropriateness of abstract, introduction and conclusions

From a presentational point of view, I strongly recommend reworking the paper to include more sign posts and expository material for the reader to explain how the various parts fit together.

I would also advise to remove the claim in the abstract the claim about "6,876 meningioma samples" because it gives a misleading representation of the work, and is not needed to convey the significance of the results.

The section on the training and used of a GAN for classification should be reworded to clarify its contribution to the overall study.

Minor issues to amend:

- typo "To investigates"
- typo "management can dependent"
- typo "have significant better outcomes"
- typo "most these probes"
- typo "majorly"
- typo "in significantly different" (missing "a")
- "Less clear-cut CNVs in cases with lower scores can already be suggestive for heterogeneity" ->
not very clear - I assume this means 'low scores' correlates with cellular/genetic heterogeneity?
- typo "can dependent"

Version 1:

Decision Letter:

13th Jun 2025

Dear Professor Sahm,

Your Article, "A microenvironment-determined risk continuum refines subtyping in meningioma and reveals determinants of machine learning-based tumour classification" has now been seen by 3 referees who have some minor requests.

We therefore invite you to revise your manuscript taking into account all reviewer and editor comments. Please highlight all changes in the manuscript text file. At this stage we will need you to upload a copy of the manuscript in MS Word .docx or similar editable format.

We will likely assess your revisions in-house and will only return to reviewers if absolutely necessary.

We are committed to providing a fair and constructive peer-review process. Do not hesitate to contact us if there are specific requests from the reviewers that you believe are technically impossible or unlikely to yield a meaningful outcome.

When revising your manuscript:

*1) Include a “Response to referees” document detailing, point-by-point, how you addressed each referee comment. If no action was taken to address a point, you must provide a compelling argument. This response will be sent back to the referees along with the revised manuscript.

*2) If you have not done so already please begin to revise your manuscript so that it conforms to our Article format instructions, available
here.
Refer also to any guidelines provided in this letter.

*3) Include a revised version of any required Reporting Summary: https://www.nature.com/documents/nr-reporting-summary.pdf
It will be available to referees (and, potentially, statisticians) to aid in their evaluation if the manuscript goes back for peer review.
A revised checklist is essential for re-review of the paper.

Please be aware of our guidelines on digital image standards.

EXTENDED DATA FIGURES

When re-submitting your manuscript, please ensure that any supplementary figures and tables that are crucial to the manuscript’s conclusions are converted into Extended Data figures and tables to increase visibility of these data. Extended Data figures and tables are online-only (present in the online PDF and full-text HTML versions of the paper), peer-reviewed display items that provide essential background to the article but are not included in the main article due to space constraints. A maximum of ten Extended Data display items (figures and tables) is permitted.

Please use the link below to submit your revised manuscript and related files:

Link Redacted

Note: This URL links to your confidential home page and associated information about manuscripts you may have submitted, or that you are reviewing for us. If you wish to forward this email to co-authors, please delete the link to your homepage.

We hope to receive your revised manuscript within four to eight weeks. If you cannot send it within this time, please let us know.

Please do not hesitate to contact me if you have any questions or would like to discuss these revisions further.

Nature Genetics is committed to improving transparency in authorship. As part of our efforts in this direction, we are now requesting that all authors identified as ‘corresponding author’ on published papers create and link their Open Researcher and Contributor Identifier (ORCID) with their account on the Manuscript Tracking System (MTS), prior to acceptance. ORCID helps the scientific community achieve unambiguous attribution of all scholarly contributions. You can create and link your ORCID from the home page of the MTS by clicking on ‘Modify my Springer Nature account’. For more information please visit please visit www.springernature.com/orcid.

We look forward to seeing the revised manuscript and thank you for the opportunity to review your work.

Sincerely,

Safia Danovi, PhD
Senior Editor, Nature Genetics
ORCiD: 0009-0007-7822-5479

Reviewers' Comments:

Reviewer #1 (Remarks to the Author):

I would like to commend and congratulate the authors on their thorough and thoughtful revision, and careful responses to each and every one of my suggestions for improvement (which were admittedly voluminous). The revised study now sets a new standard in an already-exciting era of integrated histological and molecular classification of cancer, and of brain tumors in particular. The integration of single-nuclei, spatial, and bulk bioinformatic approaches alongside traditional histological techniques in this study not only provides fundamental insights into the most common primary intracranial tumor but is also potentially practice changing as the authors’ data relate to the classification of meningiomas. In sum, this study provides a framework and a roadmap that is simultaneously innovative and accessible for integrating bioinformatic and histological

techniques to refine our understanding and classification of brain tumors. I would additionally note that this study is likely to inspire future investigations of the differences between immune populations (and microenvironment cell types more broadly) in different molecular groups of meningiomas, but my personal perspective is that such analyses (receptor-ligand, functional assays, etc.) are beyond the scope of this investigation, which is complete in it's current form.

Reviewer #2 (Remarks to the Author):

The authors have adequately addressed all points I had raised.

Reviewer #3 (Remarks to the Author):

This is the first revision of the paper: "A microenvironment-determined risk continuum replaces (now: refines) subtyping in meningioma and reveals determinants of machine learning-based tumour classification, submitted to Nature Genetics". Given that an extensive summary of the paper was provided in the previous round of reviews I will not recapitulate the paper again here.

I thank the authors for their careful consideration of the comments made by reviewers on the original submission and their efforts to address the points raised.

This is a complex paper and I am pleased that the authors have made attempts to include more sign posts to help readers follow the underlying processes and logic. I think it has been improved somewhat (though I maintain that it is a challenging piece of work to follow). I am a bit surprised in a paper with this level of complexity that no overarching figure for the experiments/methodology is provided. Perhaps Figure 1 could be expanded with a sub-figure that summarises the methodology(ies) and samples used?

The revised paper clarifies the number of participants/samples in the study, which was a point of confusion in the original version. Importantly, the abstract (and paper) now does not overstate the number of samples used in the study.

The 3-hop subgraph model method has now been published in a separate manuscript, which benefits the current paper in avoiding the need for an overly lengthy description. An effort was made to validate the results of this model on an external data set (Lucas et al). While overall accuracy remains good (dropping from 0.97 to 0.93), precision, recall and F1 drop substantially. This could be because the validation set is very small (only 16 tumours), it could also be a sign of overfitting in the original classifier (as originally suggested in the first reviews). I think we should take the results of the GAN classifier with some level of healthy suspicion until much larger training and validation sets are available (recognising the difficulty of getting such large sets). I would encourage the authors to comment in the next revision of the paper on the potential for overfitting and the relatively small data sets used here.

The use of more nuanced "refines" instead of "replaces" in the title is a very sensible amendment.

I am sceptical about the point raised in the Materials and Methods regarding batch correction "To avoid batch-correction obscuring biological effects, only samples that were processed in one batch were included, avoiding the need for batch correction". This sounds to me like the authors were in a hurry and decided to take the most expedient route (and I can sympathise with this tendency). However, methods such as the RUV family of statistical normalisation/correction techniques have been shown to effectively retain biological information while taming batch effects. I would recommend further consideration whether the batches really need to be abandoned.

Overall this is an impressive body of work, and an important step forward in understanding the variation observed in meningioma progression and outcomes. It has improved substantially since the initial submission. I would consider the comments I made above to be relatively minor in the scheme of things and therefore I think the paper should be considered for publication after minor adjustments.

Version 2:

Decision Letter:

Our ref: NG-A67028R1

18th Jul 2025

Dear Dr Sahm,

How are you? I hope you're well.

Thank you for submitting your revised manuscript "A microenvironment-determined risk continuum refines subtyping in meningioma and reveals determinants of machine learning-based tumour classification" (NG-A67028R1). It has now been seen by the Nature Genetics editorial team and I'm delighted to say that we'll be happy in principle to publish it in Nature Genetics, pending minor revisions to comply with our editorial and formatting guidelines.

If the current version of your manuscript is in a PDF format, please email us a copy of the file in an editable format (Microsoft Word or LaTex)-- we can not proceed with PDFs at this stage.

We are now performing detailed checks on your paper and will send you a checklist detailing our editorial and formatting requirements soon. Please do not upload the final materials and make any revisions until you receive this additional information from us.

Thank you again for your interest in Nature Genetics Please do not hesitate to contact me if you have any questions.

Sincerely,

Safia Danovi, PhD
Senior Editor, Nature Genetics
ORCiD: 0009-0007-7822-5479

Open Access This Peer Review File is licensed under a Creative Commons Attribution 4.0 International License, which permits use, sharing, adaptation, distribution and reproduction in any medium or format, as long as you give appropriate credit to the original author(s) and the source, provide a link to the Creative Commons license, and indicate if changes were made.
In cases where reviewers are anonymous, credit should be given to 'Anonymous Referee' and the source.
The images or other third party material in this Peer Review File are included in the article's Creative Commons license, unless indicated otherwise in a credit line to the material. If material is not included in the article's Creative Commons license and your intended use is not permitted by statutory regulation or exceeds the permitted use, you will need to obtain permission directly from the copyright holder.
To view a copy of this license, visit https://creativecommons.org/licenses/by/4.0/

REVIEWER #1 (meningioma, genomics)

The authors of the manuscript entitled "A microenvironment-determined risk continuum replaces subtyping in meningioma and reveals determinants of machine learning-based tumour classification" aim to provide a new tumor microenvironment-based meningioma risk assessment. To do so, they use a total of 6876 meningioma samples and performed multiple experimental approaches such as snRNA-seq and bulk DNA methylation along with extensive computational and machine learning approaches to arrive at their conclusions. By the end of the manuscript, I was convinced by the authors that meningiomas display a continuum rather than discrete groups/subgroups and techniques such as DNA methylation or RNA sequencing only provide a static snapshot of what is actually going on inside the tumor. However, this does not make the previously reported groups/subgroups invalid in classifying meningiomas (e.g. one does not "replace" the other) but rather prompts the use of more sensitive approaches to interrogate meningiomas at a single cell level, which highlights the underlying tumor heterogeneity that may easily be missed by bulk approaches. Despite this rhetorical faux pas, the overall study design is exceptional, and the conclusions are otherwise well supported by the data. This study will provide a significant conceptual advance in our understanding of the fundamental biology of the most common brain tumor, and I hope to have the opportunity to review a revised version of this manuscript.

Response:
We thank the reviewer for the time taken to review our manuscript, the nice summary of the work and the supportive comments regarding our manuscript. We worked hard to generate a revised manuscript with almost all of the suggestions/comments/recommendations of the three reviewers included. We hope that the reviewer appreciates the revised version of our manuscript even more than the original.

Major suggestions

1. The 3-hop subgraph analysis of spatial transcriptomic data is thoughtful and interesting, but more details on the development and validation of this classifier that transforms meningioma MCs onto spatial transcriptomes is needed. If the authors did not split their "43 slides" (Is that 43 separate tumors? Or 43 sections from one tumor? Later in the paper, the authors state "43 cases"… what does this mean?) into discovery and validation cohorts, they are encouraged to use the data from Lucas et al. Nature Genetics 2024 to validate their classifier and report only the precision, accuracy, recall, and F1 scores from independent validation cohort (not discovery cohort) samples.

Response:
Thank you for the interest in this approach. The development of the 3-hop subgraph model has been published after the submission of this manuscript (Ritter et. al. Nature Cancer 2025). All technical details are comprehensively discussed in that publication dedicated to this method and therefore to save space we do not extend our description of the technique further (also since reviewer #3 asked to reduce this section in length) but reference to the Ritter et al. publication instead.

The 43 slides/cases description is indeed confusing. Thank you for spotting this imprecise language. These were neither samples nor cases, but runs, in part with more than one class-matched sample. More clearly: we eventually investigated 42 samples from 37 individual meningioma tumours. This information is now included in the revised manuscript.

We appreciate the suggestion to validate our model using the data from the Lucas et. al. publication. Validating our model in an external dataset increases the impact of our findings and therefore results from this additional experiment are now included in the revised manuscript.

Revisions made in response to the comment:

1. A reference to the Ritter et. al. publication is added to the manuscript
2. On lines 191, 399 and 990 we have now explicitly described that the discovery spatial RNAseq data is from 42 samples from 37 independent meningioma
3. The results from the spatial RNAseq validation cohort are added on lines 198-200 and in Suppl. Figure 3C.

2. The authors appropriately note in the Introduction that previously published meningioma molecular classifiers have identified groups of tumors that are defined by immune signatures and immune cells (Choudhury et al. and Nassiri et al. in Figure 1A), but almost all of the analyses in their figures are limited to a molecular classifier (Sahm et al. Lancet Oncology 2017) that was not initially built around the observation that immune cells may influence the molecular classification of meningiomas. The breakdown of microenvironment signatures/cells and neoplastic signatures/cells from their various analyses (single-nuclei, spatial, imaging, deconvolution, etc) should at the very least also be shown across molecular groups of meningiomas (Choudhury et al., as the authors have done in Figure 5). Analysis across meningioma subgroups is not necessary, as subgroups of meningiomas are not known to be distinguished by microenvironment composition.

Response:
We agree with the reviewer that our results should be shown beyond just one of the established molecular based risk-prediction models for meningioma. We have therefore added additional analyses for each subfigure containing data for the methylation classes from the Sahm et. al. Lancet Oncology publication. As suggested by the reviewer we have included data for the molecular groups from the Choudhury et. al. Nature Genetics publication. The results put forward in the original publication are replicated and confirmed for the immune-enriched, Merlin intact and hypermitotic molecular groups (MGs). Adding this information makes our manuscript much more robust and we therefore thank the reviewer again for the suggestion. Due to the large set of additional subfigures, we have placed most of the data for the molecular groups in supplementary figures.

Revisions made in response to the comment:

1. The following supplementary figures were added:
    - Suppl. Figure 1B: snRNA UMAP showing MG distribution

- Suppl. Figure 1D: shRNA quantification of TAMs for MGs
- Suppl. Figure 1E: CD68 immunohistochemical quantification for MGs
- Suppl. Figure 1F: shRNA quantification of microglia within TAMs for MGs
- Suppl. Figure 2C: Marimekko plot showing TAM subset distribution for MGs
- Suppl. Figure 8B: NMF-signature distribution for the discovery cohort of 4502 meningioma for MGs
- Suppl. Figure 8C: NMF-signature distribution for the validation cohort of 970 meningioma for MGs
- Suppl. Figure 11B: GAN spatial RNAseq MG prediction
- Suppl. Figure 11D: primary-recurrence flow for MGs
- Suppl. Figure 12A: deconvolution celltype distribution for the clinical cohorts for the MGs

3. The analyses in Supplementary Figure 2 were interested and appreciated, but CD81 and SPP1 feel cherry-picked. Could the authors take all of the CpG probes mapping to differentially expressed genes in Supplementary Figures 1C and 1D and look at the methylation and expression of these genes, as has been done for CD81 and SPP1 in Supplementary Figure 2?

Response:
Describing and showing only a handful out of all 10,000 CpG probes involved with meningioma subclassification can indeed suggest cherry-picking. We have therefore decided to make several changes to be more transparent about the process. First, we have added a supplementary table with information on all 10,000 CpG probes and their role in meningioma subclassification. To elaborate on the methods for probe identification we now also reference the recent publication (Benfatto et. al. Nature Communications) that describes the method in great detail. Lastly, we have changed suppl. figure 2 (in the revised manuscript suppl. figure 4) where subfigure B no longer show CD81 and SPP1 (they are still discussed in the text) and the new subfigure E shows all significantly up- and downregulated genes mapping to CpG probes from subfigure B.

Revisions made in response to the comment:

1. Supplementary table 1 is added with information on all 10,000 CpG probes and their role in meningioma subclassification.
2. A reference is added to Benfatto et. al. Nature Communications 2025 with in-depth information on classifier probe extraction.
3. Suppl. figure 4 subfigures B and E changes/added

4. The authors make quantitative conclusions from the UMAP visualization in Figure 3C without showing a quantitative analysis (e.g. the reader is expected to rely on UMAP gestalt). The authors should calculate the proximity of each meningioma to other meningiomas with the same DNA methylation class vs the proximity of each meningioma to other meningiomas with different DNA methylation classes using all

signatures vs neoplastic signatures vs stromal signatures. See Liu et al. Genome Biology 2024 for a similar analysis. The same analysis is suggested for Figure 4A.

Response:
Adding quantitative information adds value to the subfigures listed by the reviewer and we therefore appreciate the suggestion. After careful consideration we concluded that the method used in Liu et. al. Genome Biology 2024 may not be directly applicable to methylation beta values (of note: in 2024 there were 7 publications by a first author called Liu in Genome Biology so we may have looked at the wrong subfigure). Therefore, in looking for a workable approach inspired by the approach in Liu et al. 2024, we concluded that a better method for quantifying the distance between clusters, is to use the mean pairwise cosine distance as, by definition, this metric is used for comparison of groups of vectors. In our experience, the use of cosine distance is also better suited than euclidean distance to quantify meaningful differences in beta values as their values are bounded between -1 to 1. As suggested, we then applied this strategy to target the specific questions relevant to figures 3C and 4A, by adding the relevant quantifications comparing orientation within subclasses and between subclasses to figure 3C and suppl. figure 8C respectively.

Revisions made in response to the comment:

1. Results from the mean pairwise cosine distance calculations were added on lines 301-308 and 353-356
2. Figure 3C was extended with the right-panel
3. Suppl. figure 9B was added corresponding to figure 4A.

5. It is important to understand if the CNAs from Figure 2B (that come up again in Figures 3 and 4) segregate in single neoplastic nuclei from Figure 2A as would be expected across MCs (Sahm et al.) or DNA methylation groups (Choudhury et al.). The pipelines used for development of these two molecular classifiers were inherently different, with Choudhury et al. using a pipeline that controlled for biases from CNAs for clustering. It would be good to know which classification system provides better segregation of CNAs in cells derived from tumors of different molecular groups.

Response:
We appreciate the point raised by the reviewer and have added additional analyses based on the comment. As the CNV-clusters are identified from cells of one tumor that has an overarching methylation class (MC) or methylation group (MG) annotation, we couldn't completely execute what the reviewer suggested, since overlaying cells from the snRNA CNV-clusters onto the UMAP would yield the same MC/MG. Instead, we calculated the MC/MG likelihood score based on RNA-expression for each cell and plotted this for the different CNV-clusters (Suppl. Figure 1C) in Figure 2B. These data show some difference between the MC/MG likelihood between the CNV-Clusters, but not as dramatic as would be expected if the MC/MG annotation would completely differ due to differences in underlying methodology.

Revisions made in response to the comment:

1. The CNV-clusters are more explicitly annotated in Figure 2B and Suppl. Figure 1C.
2. RNA scores for the MC/MG per CNV-clusters are added to Figure 2B and corresponding results are added on lines 104-121.

6. The last paragraph of the Results that is associated with Figure 4, where the authors assert that their data solve “several current controversies and uncertainties in meningioma research at once”, is highly speculative and should be moved to the Discussion. (1) The absence of similar analyses in ependymomas or medulloblastomas, (2) the finding that glioblastomas exist along a dynamic and interchangeable molecular continuum, and (3) the absence of mechanistic and functional studies to test these hypotheses need to be specifically mentioned in the Discussion.

Response:
The reviewer raises an important point and we agree that the referred text better fits the discussion than the results section. We have removed the text from the results and expanded the discussion covering the points made in the previous version of the results section. Here we have also included the suggestions to discuss the absence of similar and mechanistical experiments in ependymoma and medulloblastoma. Even with extensive searching we couldn’t find similar studies performed in glioblastoma so for now, we have not included this in the revision.

Revisions made in response to the comment:

1. We’ve moved the contents of the last paragraph of the results section to the discussion and extended this section with a discussion on mechanistical and similar experiments that should still be performed in ependymoma and medulloblastoma on lines 540-547 and 559-561.

7. The stratification of meningioma clinical outcomes in the retrospective and prospective validation cohorts in Supplementary Figure 7 is not great, and only PFS is shown in all KM curves throughout the paper. This limitation needs to be appropriately mentioned in the Results and in the Discussion, and OS data need to be provided adjacent to all KM curves.

Response:
We agree with the reviewer that the stratification in the former supplementary figure 7 (now supplementary figure 12) could technically be better. However, the major goal was the clinical applicability of the approach, requiring a pragmatic set-up. For a neuropathologist investigating a meningioma it is not relevant or even applicable to work with multiple tiers of immune-cell percentages. What is relevant is identifying a high number of immune cells, or by seeing a reduced number. This is why in figure 5C and D, we work with a dichotomous model: high vs. reduced. We’ve added this on line 454-455.
Our clinical datasets containing three independent clinical cohorts currently does not contain information on overall survival. We are gathering this information, but since the different cohorts contain almost a thousand cases spanning the globe, obtaining

this information is time-consuming. Secondly, we believe that for meningioma patients that often live for decades after the first surgery, overall survival is less relevant than progression-free survival. Therefore, until the harvesting of additional information is finalized, we can't add KM-plots based on overall-survival data, but we believe that this does not compromise the impact of our manuscript.

Revisions made in response to the comment:

1. Additional information is added to lines 454-455

8. The stratification of KM curves (for PFS and OS) in Figure 6C with the addition of PU.1 IHC to WHO grade is impressive, particularly as previous attempts to distil meningioma molecular classification systems down to IHC markers (Nassiri et al) has failed to validate (Naslund et al. Brain and Spine 2023). The authors should incorporate these references into their Discussion alongside explanations for why they think their IHC approach is superior and will not be similarly invalidated in subsequent studies.

Response:
We thank the reviewer for the compliment. We too have attempted to validate the immunohistochemical subclassification put forward in the Nassiri et. al. Nature publication, but failed (data not published). One difference between our approach here and the data from Nassire/Naslund is that those publications attempt to use stainings as proxies for molecular classifications, whereas we use data from molecular classification and extract a quantitative marker (immune enrichment), that can be quantified using immunohistochemistry. As suggested, we have added the information regarding immunohistochemistry to the discussion.

Revisions made in response to the comment:

1. On lines 579-585 we've added additional information about immunohistochemical risk prediction for meningioma to the discussion

Minor critiques

1. To say that the microenvironment-determined risk continuum reported here "replaces" the well-established molecular groups of meningiomas (in the title and elsewhere in the manuscript) is an overstatement. I strongly suggest the use of "refines" instead of "replaces" throughout.

Response:
We agree that "refines" better covers the contents of our manuscript. We have made changes accordingly.

Revisions made in response to the comment:

1. The word replaces is changed to refines in the title and abstract of the revised manuscript

2. The focused Introduction is very much appreciated by this meningioma-zealot of a reviewer… but for a broad scientific audience, it is suggested that the authors perhaps contextualize their study (and foreshadow their results) in broader terms. The findings of this study have broad, wide-reaching implications for cancer classification, but as written the Introduction gives the impression that this study is narrow in scope and impact (which it is not).

Response:

As suggested by the reviewer we've extended the introduction significantly. We realize that the added details regarding molecular changes in meningioma make the manuscript easier to understand for readers outside of the meningioma field. We therefore thank the reviewer for this suggestion.

Revisions made in response to the comment:

1. Lines 30-48 and 72-79 were added to make the introduction much more comprehensive and to also introduce the results presented in the manuscript

3. In Figure 1A, it is suggested that Choudhury and Chen et al. Neuro-Oncology 2022 be referenced next to the subdivision of Hypermitotic meningiomas into Hypermetabolic and Proliferative meningiomas. It is confusing that this figure (and the paper more broadly) omits reference to the finding of 2 molecular groups of meningioma from Olar et al. Acta Neuropathology 2017. If this system was found to be not robust or was not validated in subsequent studies, this shortcoming should be mentioned and the authors are encouraged to do so in the Discussion of their manuscript.

Response:

We appreciate the detailed investigation by the reviewer. The model from Olar et. al. was initially not included since the later models are more extensive than this early work. However, we understand that omitting this paper may incorrectly suggest that the findings are no longer relevant. As we have no indication at all that the findings are incorrect or non-relevant, we've added the Olar et. al. model to figure 1A.

Revisions made in response to the comment:

1. Figure 1A is updated to include the Olar et. al. model

4. Do I misinterpret Figure 1B, or is the sex of three patients shaded as neither Female nor Male? The authors are encouraged to also shade these samples according to the DNA methylation groups from Choudhury et al. and DNA methylation subgroups from Nassiri et al. in Figure 1A.

Response:
Initially the sex of three patients was indeed not known and therefore not included in the figure. We've subsequently extracted the sex from the epigenetic information for the cases and updated the figure accordingly. Simultaneously, we've added the UCSF methylation group for each sample to figure 1B too. Since the Nassiri et. al. subgrouping data is not determinable for our cases from public resources, this information is not included.

Revisions made in response to the comment:

1. Figure 1B is updated to contain the methylation group and sex for each sample

5. Could the clustering in Figure 2C be due to batch effects? Why wasn't Seurrat integration algorithm and/or harmony batch correction performed.

Response:
In theory the observed clustering could indeed be due to batch effect. However, batch-correction can however also obscure relevant biological differences between samples. To avoid this, we have only included samples that were processed in one batch, avoiding the need for batch correction. We've added this information to the methods.

Revisions made in response to the comment:

1. Lines 650-652 in the methods section list the processing of the samples in more detail

6. Methods to calculate cell cluster abundance (Figures 2D, 2E, 2G) across samples/groups is not present. It is not clear if these data have been normalized to the total cells captured in a particular sample. I understand orthogonal validations have been performed but if this analysis is present in the manuscript, it's methodology and results should be clear.

Response:
We agree with the reviewer that more details could be added. We've decided to add this information to the figure legends of the mentioned figures (Figures 2D-G) as well as for the newly added corresponding figures for the MGs (Suppl. Figure 1D-F).

Revisions made in response to the comment:

1. Additional information regarding the calculations has been added on lines 1196-1197, 1199-1200 and 1209-1210 for figure 2 and on lines 1343-1344 for supplementary figure 1.

7. For some of the samples, the % TAM is as high as 90%. How can this be? What are the histological/morphological features of such tumors? Are these tumors regressing and not growing at all?

Response:

We agree with the reviewer that a TAM percentage approaching 90% feels too high. For snRNA sequencing it is known that sample processing can introduce a bias regarding cell type isolation. This is also the reason why we performed additional immunohistochemical and MIBI-based orthogonal methods to confirm the data obtained from snRNA sequencing. To make this clearer to the reader we've explicitly mentioned the possible artificial enrichment in the manuscript.

Revisions made in response to the comment:

1. On line 137 we've added that snRNA sample preparation can lead to the enrichment of myeloid cells

8. Figure 2J – It's not clear what is represented by the size of the circle.

Response:

We thank the reviewer for spotting the missing information. The diameter of the circles corresponds to correlation. We've added this information to the legend of the figure. This also applies to the new suppl. figure 3D.

Revisions made in response to the comment:

1. The figure legends for figure 2J and suppl. figure 3D have been extended on lines 1222 and 1371-1372.

9. While UMAP is a good tool for visualizing high dimensional data, it should ideally not be used for identifying clusters in the data. I would like to see unsupervised cluster identification using a bonafide clustering algorithm such as kNN or k-means etc. and whether it aligns with what is shown in Figures 3C and 4A.

Response:
We agree with the reviewer that UMAP alone is not an ideal clustering strategy. Much of this limitation is addressed by our response to Major Point 4 above, which uses quantitative analysis of cluster distances to tackle the specific questions addressed by each UMAP visualization. However, we agree that applying a clustering algorithm is another good strategy for addressing this matter. As k-means assumes central tendency and normality in the data clusters, which may not be suitable to methylation beta values, we chose to instead apply hierarchical clustering to both Figure 3C and 4A to further illustrate the conclusions drawn from both UMAP figures.

Revisions made in response to the comment:

1. Hierarchical clustering by all signatures, neoplastic signatures, and stromal signatures corresponding to the data points in Figure 3C is now shown in Suppl. Figure 5D.

2. For Figure 4A, hierarchical clustering dendrogram and an example clustering by cutting the dendrogram tree are now shown in suppl. Figure 9A.

10. “On top of the evidence for a striking difference in TAM abundance between low-risk and high-risk NF2 mutant meningiomas, these results suggest that differentiation and function of those cells change with malignancy.” “Yet, it remains elusive what functional and cellular implications the CpGs relevant for classification confer.” Correlation is not causation, and the authors are encouraged to revise these statements (and ones like them) in the absence of mechanistic and functional experiments to appropriately test their stated hypotheses.

Response:

Based on suggestions by reviewer 2 and 3, we’ve added additional analysis investigating the functional state of the TAMs. We believe that the addition of this data that shows significant differences in proliferation/mitotic as well as functional TAM-related pathways better supports the first of the two above mentioned statements. However, we acknowledge that with the lack of mechanistic (in vitro) experiments, we still show correlations. We have added additional sentences to acknowledge this in the manuscript. For the second statement we’ve added additional information contextualizing the statement.

Revisions made in response to the comment:

1. Lines 540-547 are added that explicitly acknowledge that mechanistic experiments are required to confirm the observed correlations.
2. For the second statement we have added a nuancing statement on lines 234-235.

11. Figure 4D and 4E – Does this point towards the possibility of differential immune cell recruitment and/or evasion capabilities by different tumor subtypes within the same tumor? Or differential tumor evolution at different sites within the same tumor?

Response:

We too find these observations very interesting. However, to fully investigate this and to avoid overstating correlations for causations as pointed out by the reviewer in minor point 10, we believe that investigating this properly is beyond the scope of the current manuscript. In general, we believe that with increased aggressiveness of the tumour, our data does suggest a change in immune cell differentiation and activation. This does not fully answer the reviewer’s question though, as our analyses regarding activation states does not include comparisons within one tissue block. To properly investigate this, snRNA analyses from multiple tumors for $CD68^{high}$ and $CD68^{low}$ areas would be required, a complete project on its own.

Revisions made in response to the comment:

So far, no changes were made.

12. What are the red and green cells in Figure 6F?

Response:

The schematic overview of our findings in figure 6F, highlight the fact that cells from different subclones/MC are present in different configurations. The cells in the cartoon correspond to the colors used for the MCs throughout the manuscript. We've made this more explicit by including lines and labels for the different subclones in the cartoon.

Revisions made in response to the comment:

1. Lines and labels are added to figure 6F to better indicate the different subclones/MC

REVIEWER #2 (neuropathology, epigenetics)

In this paper by Mass and co-authors, multi-omic profiling and multiple linesof orthogonal computational evaluation of a large cohort of meningioma samples, led to the identification and characterisarion of changes in the tumour microenvironmentwhich contributed to the epigenetic classification of the tumours and enabled prediction of patients outcome. They also provide evidence to support the interpretation that a TME-determined risk continuum is potentially more informative in terms of prognosis assessment than classifying meningiomas into distinct subgroups. This is an impressive work, the approach is original, the methodology is state of the art, the results are very well presented. The patients cohorts used are impressively large and this ensures the conclusion of the paper are strong. Overall, the conclusion that the proposed changes to TAM (epigenetics and IHC staining) and the evidence that intratumour heterogenity can lead to the misdiagnosis of patients is a very valuable contribution to the field. At times the authors overstate the novelty or unexpectedness of their findings - for instance I don't think anyone would be surprised that the non-tumour cells are contributing to the bulk Heidelberg classification. Having said that, the paper addresses an important diagnostic issue in the field and has the potential to significantly contribute to a better stratification of meningiomas, which is a significant medical issue, ie not been able to predict which grade 1 tumours will recur and behave more aggressively. The spatial analysis and teasing apart what is causing the classifier diagnosis is not for me to assess as it does not fall within my field of expertise but all seem reasonable.

Response:
We thank the reviewer for the extensive review of our manuscript and are happy with the positive words regarding the originality, the methodology and final conclusions. We acknowledge the fact that theoretically it makes sense that non-tumour cells contribute to bulk Heidelberg classification. However, in our experience proof for this has been asked for many times before at conferences and in previous reviewer

comments, including the original Capper et. al. Nature publication introducing the Heidelberg brain tumor classifier. In this manuscript we now provide data to support this concept. However, we do understand the point raised by the reviewer and have made changes accordingly, also based on reviewer #1 minor point 10.

Revisions made in response to the comment:

1. Textual changes already listed under reviewer #1 minor point 10
2. Regarding the above-mentioned statement, we've added text to better contextualize and quantify the findings on lines 266-269, 301-311 and 352-356 as also suggested by reviewer #1 major point 4.

Other points

1. What is driving the large difference in Int-B macrophages from all other macrophages in Fig1A? I might have thought this would result in a unique immune signature for this MC subtype from their deconvolution analysis?

Response:
We agree with the reviewer that the distinct localization of the int-B TAMs in the UMAP presented in figure 1A may hint at a distinct expression profile for int-B TAMs. However, we believe that we have too few datapoints to make strong conclusions on this. In our dataset we have only 2 int-B snRNA samples included of which 93.1% of cells belong to one of the two samples. We therefore don't want to include too strong conclusions in our manuscript regarding the int-B snRNA expression profiles. We do however now include the int-B cells in the Marimekko plot included in suppl. figure 2C. In this analysis we see a similar distribution of the TAM subsets as obtained for the other MCs. Our limited data does therefore not immediately confirm the suggestion that int-B TAMs are very different from the other TAMs.

Revisions made in response to the comment:

1. We now include the Marimekko plot in suppl. figure 2C that shows both the absolute number of cells as well as the TAM subset distribution.

2. Microglia number changes are more striking than those of the macrophages (Fig.1J), is this biologically relevant?

Response:
We appreciate the suggestion by the reviewer, however we don't think that this can be concluded from a correlation plot. However, other data in our manuscript can help to identify number changes for microglia and macrophages. When we look at figure 2G we see that within the TAM compartment the percentage of microglia decreases from a median of 24% for Ben-1 to 7% for Mal, so just over a 3.4 foldchange. This also means that the number of macrophages goes from 76% to 93%, so a 1.22 fold increase. This does indeed show a more pronounced relative change for microglia than macrophages.

This pure numeral approach can be valuable for risk-prediction efforts when quantifying the number of microglia, however this fold-change may not represent a biological relevant difference in microglia/macrophage function. For this we've added additional pathway enrichment analyses regarding the activation state of the microglia and macrophages (see also reviewer #2 minor point 3 below) where we see more pronounced and more biologically relevant changes for macrophages compared to microglia.

Revisions made in response to the comment:

1. The revised manuscript now includes snRNA pathway gene set enrichment analyses (GSEA) comparing microglia and macrophages between low-risk and high-risk meningioma in supplementary figure 3A and B
2. The above data was confirmed in the spatial RNAseq data as shown in the new supplementary figure 3D
3. Lines 171-179 and 212-215 were added discussing the above results

3. The analysis of the TAM/MG should be developed further, comparing and contrasting how they behave in the different MC subclasses - perhaps they could use the spatial data to do some receptor ligand analysis etc? This is essential to fully capitalise on the approach in terms of novelty of the findings.

Response:
We thank the reviewer for their suggestion and added additional analyses as suggested. We believe that the gene set enrichment analyses and confirmatory spatial RNAseq data showing pronounced differences in mostly the macrophages that draw parallels to tumour-associated/tumour-supportive macrophages in other (brain) tumours make the revised manuscript much stronger.

Revisions made in response to the comment:

1. Refer to the previous point of reviewer #2 for the changes to the figures and manuscript.

4. There are a lot of Kaplan Meyers plots without significance values.

Response:
We appreciate the reviewer for pointing out this omission. Since the KM-plots that did not contain p-values show multiple sets of data, we believe that pairwise p-values comparing the most relevant groups add the most value. We have now added relevant p-values to all KM-plots.

Revisions made in response to the comment:

1. Pairwise comparative p-values were added to the following subfigures:
    a. Figure 5E
    b. Figure 6C

c. Suppl. Figure 13B (previous Suppl. Figure 8B)
d. Suppl. Figure 13C (previous Suppl. Figure 8C)

REVIEWER #3 (computational, brain tumours, TME)

A. Summary of the results

The current state of the art approach to classification of meningiomas (like many other brain tumours) is based on molecular profiling of bulk tumour tissue samples, particularly employing machine learning classification of DNA methylation array data.However, discrepancies arise across different classifiers (and the established WHO grades) particularly in the case of NF2 mutated tumours, whereas NF2 wild type tumours are generally consistently classified.This paper investigates the hypothesis that NF2 mutant meningiomas present molecularly as a continuum, rather than neatly defined subtypes. They propose that this contiuum is strongly affected by the abundance and functional behaviour of non-neoplastic cells, particularly tumour associated macrophages (TAMs). They observe that the proportion of TAMs is low in NF2 wild type tumours, and in NF2 mutant tumours that proportion of TAMs decreases with higher grades (which is interestingly opposite to what is seen in other brain tumours). Also, they observe that the composition of TAMs changes with tumour grade. These differences can be used for better diagnostics. While tumour molecular profiling, especially multi-modal, yields the most precise results, the paper also shows that relatively simple tissue staining for the TAM-related marker PU.1 can also be used to discriminate grades, and therefore suggests a fairly cost-effective method of classification.

B. Originality and significance

This study builds and elaborates on previous results from the authors relating to the development of precise methylation-based machine learning classifier for meningiomas. While previous work has shown the efficacy of those methods, the biological explanation of the results has remained elusive. The significance of this new paper is in providing a better understanding of what molecular/cellular factors contribute to differences in proposed meningioma classes. In particular this paper greatly advances our understanding of the important role played by the TME, particularly TAMs, in identifying markers of meningioma progression. Previously this has been difficult to understand, particularly with regards to NF2 mutant tumours. I believe the results of this paper are original and highly significant. As noted in my later remarks about the method, the paper is also very thorough in its investigation of the overall hypothesis, employing several complementary approaches to derive its conclusions.

C. Data and methodology

There are a lot of parts in this paper, making it difficult to fully comprehend on an initial reading. According to my count there are at least 10 different experiments described in the results, and at times it can be difficult to follow how they all fit together. Indeed it took me a very long time to review the paper because I had to carefully re-read it

several times over, taking many notes, to make sure I understood the methodology. I do not think that this is a problem with the methods, rather their presentation, and perhaps the need to squeeze it all into one manuscript. I think the presentation would be greatly improved with more "sign posts" for the reader, and perhaps a summary figure that ties all the experimentation together. Related to this point, given the very large number of experiments described there is a correspondingly large number of data sets used, and it can be very difficult for the reader to keep track of all them and how they relate to each other. A good example of this is that the abstract claims "evaluation in 6,876 meningioma samples": while this may be true in aggregate (and I found it hard to check), the large number of samples mentioned are spread across many different experiments, and I think it is not accurate to present the study in this way. This complexity is also apparent in the very large number of sub-plots within each of the main result figures, especially Figure 2.

Below I briefly summarise each of the main parts in the methods:

- snRNA applied on 26 samples, 44,266 cells, subjected to unsupervised clustering. CD68 expression, TAM cluster enriched for WHO grade1, also TAM cells from higher grade tumours cluster together. TAM abundance is low in NF2 wildtype and TAM abundance decreases with grade for NF2 mutants. Opposite of what is seen in other brain tumours.

- the above findings were validated by CD68 IHC on 120 meningiomas, plus mutliplexed ion beam imaging on WHO grade 2 and 3, finding that the composition of TAMS differs between stages

- spatial transcriptomics was applied to 43 slides, trained a GAN to predict MC based on local TME. This part is not clearly explained in the paper, and its significance is not clear. Accuracy etc is high, but it is not apparent whether that is just overfitting the data

- Single-cell deconvolution of each microenvironment subgraph, and subgraphs from individial samples were stratified by risk. Showed reduced microglia-like cells and increased TAMS

- Investigated correlation between CD68 protein expression and methylation probes in 120 meningiomas when subjected to Brain Tumour Classifier (random forest algorithm), and looked for probes that are significant to classification. Found that probes used to pick ben-1 from other classes were correlated to CD68 expression. Maybe CpGs hypomethylated in TAMs underlie the separation. Then Considered RNA expression of genes related to the probes that were either related to CD68-hypomethylation or specific to MC ben-1 classification. Found increased expression of these genes in the TAM population. Thus the random forest classifier is likely to be picking probes that are related to genes with increased expression in TAMs, especially in MC ben-1.

Factorisation (NMF) of 4502 bulk methylomes, resulting "signatures" were matched against methylation profiles from published isolated cell types, yielding 6 neoplastic singatures and 7 stromal signatures. UMAP clustering showed that MC groups clusterd more strongly when stromal signatures were used, compared to neoplastic signatures only. Characteristic CNV signatures were then investigated against the

NMF-defined signatures. It was found that typical risk-related CNV patterns could be identified with different NMF-signatures. Whereas NF2-wildtype have a homogeneous profile of a small number of stromal and neoplastic signatures, the NF2-mutant feature more complex mixtures.

- analysis of sub-clonal heterogeneity. Spatially resolved transcriptomic data from 43 cases, inferCNV + hierarchical clustering, used the GAN model to predict MC assignment based on spatial archetecture and cell type composition. In 16 cases with 38 different clines, nearly half had heterogeneous spatial distribution of MCs, although MC mal was more homogeneous in malignant samples.

- Investigated stability of MCs in recurrent meningiomas showed significant switching of MCs for NF2-mutants, suggesting heterogeneity in molecular states within tumours

- Assessed bulk methylation from three clinical cohorts (sizes not mentioned in the main text). Each case annotated for immune cell enrighment. It found that high immune cell enrichment was identified for WHO grade1 and MC ben-1 cases. Stratification into low, medium and high immune cell proportion was associated with differences in clinical courses. Findings also showed that immune cell proportion could be descriminatory between WHO grades 1 and 2

Lastly immune cell proportion calculation was investigated using tissue staining for PU.1. Showing that standard (and cheaper) lab techniques can be used to estimate TAMs, which can then be used to inform diagnostics without molecular profiling

D. Appropriate use of statistics and treatment of uncertainties

The statistical and bioinformatics methods applied in the paper are appropriate and, for the most part, thoroughly described in the supplementary materials. The description of the GAN for classifying spatial transcriptomics on 43 slides was probably overkill for the main body of the text, and overall will probably be difficult for the target audience to follow. Excellent precision/recall results were obtained, but some questions remain about the capacity for generalisation, given the relatively limited amount of data used in training and validation.Unfortunately the main software employed in the paper is only available upon request from the authors so it is not possible to assess its overall quality or validity, and we must therefore take the authors on trust.

E. Conclusions: robustness, validity, reliability

While there is undoubted complexity in the experimental methods (and sheer number of them) in this paper, I cannot fault the overall conclusions drawn. While the authors briefly allow themselves to speculate about the mechanisms behind the observed phenomenon, they are fairly circumspect in this regard, and I also believe it is best to be cautious at this point. Further study may be required, now that a strong hypothesis has been established. Although some readers may have preferred additional exploration into why meningiomas are apparently so different to other brain tumours in this regard.

Response:
We thank the reviewer for the extensive review and summarization of our manuscript. Reading the detailed description really makes us appreciate the effort undertaken to review our manuscript. We acknowledge that many different parts and experiments are integrated into one manuscript. We believe that the scientific audience is better served with one "big" publication tying many different things together than multiple "small" publications forcing the reader to connect the different findings themselves. We appreciate that summarizing the total number of cases analyzed with 1 number in the abstract does not optimally reflect all of the different experiments performed. We have changed the text accordingly.

The reviewer briefly mentioned that the trained GAN could be overfitting but does not ask for validation experiments. However, reviewer #1 did, so we tested the model in an independent cohort (Lucas et. al. Nature Genetics).
The reviewer also mentions that the sizes of the clinical cohorts was not included. We added a clarification on this also to the main text.

Revisions made in response to the comment:

1. On line 8 we have removed the statement regarding "6,876 meningioma"
2. The developed GAN spatial network is not validated in an independent cohort. Refer to reviewer #1 major point 1 for additional information and the changes applied.
3. On line 280 the number of cases per clinical cohort are now explicitly mentioned.

F. Suggested improvements: experiments, data for possible revision

There are 2 main methodological points requiring a response from the authors:

1. a major issue is that the study makes a big point of macrophage numbers, distinguishing disease progression states. In the figures/graphs "immune cell" data is shown without explaining which immune cell types -are they measuring all immune cell types or only myeloid cells: macrophages /microglia only. Authors should clarify.

Response:
After carefully re-reading of the manuscript, we understand the point raised by the reviewer. On multiple places in the manuscript, we now explicitly mention the type of immune cells where possible.

When re-evaluating the deconvolution method used in figure 5, we did identify that the classifier for meningioma subclassification was based on the independent meningioma classifier as explained in supplementary figure 4. This is different from the data in figures 3 and 4 and therefore the oncoplot and histogram of figure 5A and 5B are updated to be concordant with figures 3 and 4. This does not change any conclusions.

Revisions made in response to the comment:

1. Where possible we now explicitly mention the type of immune cells. Specific changes have been made on:
    - Line 137
    - Line 144
    - Line 152
    - Lines 154-155
    - Line 156
    - Lines 171-179
    - Lines 214-215
    - Line 444
2. Figures 5A and 5B were updated to us brain tumor classifier v12.5 for MC annotation

2. In terms of mechanisms relating to myeloid cell infiltration - can the authors comment on whether the myeloid cells exhibit immunosuppressive or inflammatory phenotypes & changes i these states during disease progression?

Response:
We thank the reviewer, as well as reviewer #2, for suggesting us to perform additional analyses figuring out the phenotype of the macrophages and microglia. We believe that the addition of this data makes the manuscript much strong. Refer to reviewer #2 point 2 for more information.

Revisions made in response to the comment:

1. Refer to reviewer #2 point 2 for changes made to the text and figures.

H. Clarity and context: lucidity of abstract/summary, appropriateness of abstract, introduction and conclusions

3. From a presentational point of view, I strongly recommend reworking the paper to include more sign posts and expository material for the reader to explain how the various parts fit together.I would also advise to remove the claim in the abstract the claim about "6,876 meningioma samples" because it gives a misleading representation of the work, and is not needed to convey the significance of the results.The section on the training and used of a GAN for classification should be reworded to clarify its contribution to the overall study.

Response:
With the very extensive list regarding the flow of the experiments and conclusion written by the reviewer, we understand that more guidance is needed. We have therefore added more “sign posts” and connections between sections of text to help the reader understand why certain choices were made for follow-up experiments. The suggestion to compress the section on the GAN classifier is difficult as contrary to this reviewer, reviewer #1 is asking for even more details on the classifier development. Since the technique is based on the recently published Ritter et. al. Nature Cancer publication, we decided to go for a compromise and keep the text as

is (no major removals and no major additions), but to refer to the Ritter. et. al. publication instead.

Revisions made in response to the comment:

1. Throughout different sections of the manuscript, we have added small and large sections of text summarizing the findings, bridging different sections and help explain why we performed subsequent analyses.
2. To facility readability, subfigures 6D and 6E have been swapped.

4. Minor issues to amend:

- - typo "To investigates"
- - typo "management can dependent"
- - typo "have significant better"
- - typo "most these probes"
- - typo "majorly"
- - typo "in significantly different" (missing "a")
- - "Less clear-cut CNVs in cases with lower scores can already be suggestive for heterogeneity" -> not very clear - I assume this means 'low scores' correlates with cellular/genetic heterogeneity?
- - typo "can dependent"

Response:
We thank the reviewer for pointing out the typos and have made changes accordingly.

Revisions made in response to the comment:

1. Throughout the manuscript the typos have been fixed

REVIEWER #1 (meningioma, genomics)

I would like to commend and congratulate the authors on their thorough and thoughtful revision, and careful responses to each and every one of my suggestions for improvement (which were admittedly voluminous). The revised study now sets a new standard in an already-exciting era of integrated histological and molecular classification of cancer, and of brain tumors in particular. The integration of single-nuclei, spatial, and bulk bioinformatic approaches alongside traditional histological techniques in this study not only provides fundamental insights into the most common primary intracranial tumor but is also potentially practice changing as the authors' data relate to the classification of meningiomas. In sum, this study provides a framework and a roadmap that is simultaneously innovative and accessible for integrating bioinformatic and histological techniques to refine our understanding and classification of brain tumors. I would additionally note that this study is likely to inspire future investigations of the differences between immune populations (and microenvironment cell types more broadly) in different molecular groups of meningiomas, but my personal perspective is that such analyses (receptor-ligand, functional assays, etc.) are beyond the scope of this investigation, which is complete in it's current form.

Response:
We thank the reviewer for the kind words and want to especially thank the reviewer again for all of the constructive comments and suggestions that greatly improved the quality and depth of the revised manuscript.

We agree that further investigation into the immune populations, including additional receptor-ligand and functional assays are beyond the scope of this, already very elaborative, manuscript. We do however see the comments as encouragements to dive deeper and further in future projects.

REVIEWER #2 (neuropathology, epigenetics)

The authors have adequately addressed all points I had raised.

Response:
We thank the reviewer, the constructive comments provided before and for the time taken to evaluate our revised manuscript. We are happy that our updated manuscript has adequately addressed the points raised by the reviewer.

REVIEWER #3 (computational, brain tumours, TME)

1. This is the first revision of the paper: "A microenvironment-determined risk continuum replaces (now: refines) subtyping in meningioma and reveals determinants of machine learning-based tumour classification, submitted to Nature Genetics". Given that an extensive summary of the paper was provided in the previous round of reviews I will not recapitulate the paper again here.

I thank the authors for their careful consideration of the comments made by reviewers on the original submission and their efforts to address the points raised.

<u>Response</u>:
We thank the reviewer for the investment made to carefully review our revision of the submitted manuscript. We are happy that the major comments were properly addressed and that only few minor items remain. We appreciate these minor suggestions and have made adjustments to this second revision as outlined below.

2. This is a complex paper and I am pleased that the authors have made attempts to include more sign posts to help readers follow the underlying processes and logic. I think it has been improved somewhat (though I maintain that it is a challenging piece of work to follow). I am a bit surprised in a paper with this level of complexity that no overarching figure for the experiments/methodology is provided. Perhaps Figure 1 could be expanded with a sub-figure that summarises the methodology(ies) and samples used?

<u>Response</u>:
After re-reading and analyzing all figures included, we agree that an overarching figure may be beneficial to the reader. Hence, we added a graphical / procedural abstract to Figure 1 as subfigure 1C. The figure displays the different steps of the manuscript and how each individual section is constructed. If the editorial team finds that the subfigure better fits as a separate figure, we will be happy to do so.

While reviewing all figures, we noticed that two dots in the dot-plot of Figure 2J had incorrect size due to an error in the script. This is corrected in the revised version. This correction has no effect on the conclusion drawn from that analysis. Hence there are no other changes to the text or elsewhere resulting from this.

<u>Revisions made in response to the comment:</u>

1. Subfigure 1C added to explain the different parts of the manuscript and what experiments are performed to investigate these different parts.
2. The text of the manuscript has been adjusted at two places to accommodate for this change:
    a. Lines 85-86 to introduce this project outline
    b. Lines 1184-1189 were added to the figure 1 legend to explain the schematic including the abbreviations used

3. The 3-hop subgraph model method has now been published in a separate manuscript, which benefits the current paper in avoiding the need for an overly lengthy description. An effort was made to validate the results of this model on an external data set (Lucas et al). While overall accuracy remains good (dropping from 0.97 to 0.93), precision, recall and F1 drop substantially. This could be because the validation set is very small (only 16 tumours), it could also be a sign of overfitting in the original classifier (as originally suggested in the first reviews). I think we should take the results of the GAN classifier with some level of healthy suspicion until much larger training and validation sets are available (recognising the difficulty of getting

such large sets). I would encourage the authors to comment in the next revision of the paper on the potential for overfitting and the relatively small data sets used here.

Response:
We thank the reviewer for pointing this out and agree with the issue raised. As the reviewer already correctly identified, there are currently no larger validation datasets available, which also emphasizes the novelty and uniqueness of our data. So we follow the reviewer's suggestion to comment on this aspect in the revised manuscript.

Revisions made in response to the comment:

1. Lines 200-203 were added to point out the drop in precision, recall and F1 and have listed the explanations listed by the reviewer.

4. The use of more nuanced "refines" instead of "replaces" in the title is a very sensible amendment.

Response:
Like reviewer #3, we also appreciate the suggestion by reviewer #1 to change the wording in the title.

5. I am sceptical about the point raised in the Materials and Methods regarding batch correction "To avoid batch-correction obscuring biological effects, only samples that were processed in one batch were included, avoiding the need for batch correction". This sounds to me like the authors were in a hurry and decided to take the most expedient route (and I can sympathise with this tendency). However, methods such as the RUV family of statistical normalisation/correction techniques have been shown to effectively retain biological information while taming batch effects. I would recommend further consideration whether the batches really need to be abandoned.

Response:
We realize that the wording used in the revision may indeed introduce unnecessary confusion. We completely agree with the reviewer that there are statistical methods, such as the ones listed above, that can tame batch effects efficiently. In our snRNA-seq experiments, we only included high-quality samples processed in one batch. This allowed us to avoid batch-effects by design and hence also batch-correction. We now realize that the sentence introduced in the revision only confused the message as in no way we tried to suggest that there are no valuable batch-correction methods available. We have therefore, again, changed this section in the material and methods slightly. In a broader context, there were earlier meningioma single nuclei-data generated in our lab (from the beginning of single nuclei-seq around 2018) which do not meet today's standards of single cell/nuclei data and are not included here.
We had also added this in the reporting summary when submitting the manuscript: "*An earlier batch of snRNA samples with sub-optimal isolation and low reads per cell was excluded after subsequent optimization of protocol yielded substantially higher QC values.*"

Revisions made in response to the comment:

1. On line 656 we trunctated the sentence mentioned above to "*Only samples that were processed in one batch were included, avoiding the need for batch correction*"

6. Overall this is an impressive body of work, and an important step forward in understanding the variation observed in meningioma progression and outcomes. It has improved substantially since the initial submission. I would consider the comments I made above to be relatively minor in the scheme of things and therefore I think the paper should be considered for publication after minor adjustments.

Response:
We thank the reviewer for the encouraging comments and hope that the (minor) adjustments made to the second revision are of sufficient quality to satisfy the reviewer.
