# Supplementary material for: A microenvironment-determined risk continuum refines subtyping in meningioma and reveals determinants of machine learning-based tumor classification
Source: Nat Genet. 2026 Feb 9;58(2):341–54. doi: 10.1038/s41588-025-02475-w (PMC12900647; doi:10.1038/s41588-025-02475-w)
Supplement: Supplementary file 2 — Peer Review File [file 41588_2025_2475_MOESM2_ESM.pdf]

# nature portfolio

Corresponding author(s): S.L.N. Maas
F. Sahm

Last updated by author(s): Oct 8, 2025

# Reporting Summary

Nature Portfolio wishes to improve the reproducibility of the work that we publish. This form provides structure for consistency and transparency in reporting. For further information on Nature Portfolio policies, see our Editorial Policies and the Editorial Policy Checklist.

## Statistics

For all statistical analyses, confirm that the following items are present in the figure legend, table legend, main text, or Methods section.

| n/a | Confirmed | |
|---|---|---|
| ☐ | ☒ | The exact sample size (*n*) for each experimental group/condition, given as a discrete number and unit of measurement |
| ☒ | ☐ | A statement on whether measurements were taken from distinct samples or whether the same sample was measured repeatedly |
| ☐ | ☒ | The statistical test(s) used AND whether they are one- or two-sided<br>*Only common tests should be described solely by name; describe more complex techniques in the Methods section.* |
| ☐ | ☒ | A description of all covariates tested |
| ☐ | ☒ | A description of any assumptions or corrections, such as tests of normality and adjustment for multiple comparisons |
| ☐ | ☒ | A full description of the statistical parameters including central tendency (e.g. means) or other basic estimates (e.g. regression coefficient) AND variation (e.g. standard deviation) or associated estimates of uncertainty (e.g. confidence intervals) |
| ☐ | ☒ | For null hypothesis testing, the test statistic (e.g. *F*, *t*, *r*) with confidence intervals, effect sizes, degrees of freedom and *P* value noted<br>*Give P values as exact values whenever suitable.* |
| ☒ | ☐ | For Bayesian analysis, information on the choice of priors and Markov chain Monte Carlo settings |
| ☒ | ☐ | For hierarchical and complex designs, identification of the appropriate level for tests and full reporting of outcomes |
| ☒ | ☐ | Estimates of effect sizes (e.g. Cohen's *d*, Pearson's *r*), indicating how they were calculated |

*Our web collection on statistics for biologists contains articles on many of the points above.*

## Software and code

Policy information about availability of computer code

| | |
|---|---|
| Data collection | The methods for data collection have been extensively described in the methods section. No software was used for data collection. |
| Data analysis | The methods used for code generation has been described in the methods section. The implementations are platform and infrastructure specific, however available from the corresponding authors upon reasonable request for non-commercial use. |

For manuscripts utilizing custom algorithms or software that are central to the research but not yet described in published literature, software must be made available to editors and reviewers. We strongly encourage code deposition in a community repository (e.g. GitHub). See the Nature Portfolio guidelines for submitting code & software for further information.

## Data

Policy information about availability of data

All manuscripts must include a data availability statement. This statement should provide the following information, where applicable:

- Accession codes, unique identifiers, or web links for publicly available datasets
- A description of any restrictions on data availability
- For clinical datasets or third party data, please ensure that the statement adheres to our policy

The snRNA sequencing data has been deposited to NCBI BioProject: PRJNA1338118 (temporary access until release to the public: https://dataview.ncbi.nlm.nih.gov/object/PRJNA1338118?reviewer=fgrhv1m5d8h1bqrm6364s6vhq3). The raw data for spatial sequencing data has been deposited at NCBI BioProject: PRJNA1338697. Additional data files have been deposited to https://zenodo.org/records/17296826 (with the following share link:https://zenodo.org/

records/17296826?
preview=1&token=eyJhbGciOiJIUzUxMiJ9.eyJpZCI6ImE2YWU2ZGY3LTg4NTctNDU3ZC04NGIyLWUwZjJlMDgxMGI4YiIsImRhdGEiOnt9LCJyYW5kb20iOiJkZGYyZWFjMDM3NWQ5ZDNlODRlNDVmOWZkODQ3YWYwOSJ9.quY-ynFfvYD2zzTb6zKA7y53U-EoiEkljv1L7tu20iBxEXnF7p8pUInArgmp6_WqMIe17MAYiXEUgIrAZNYCKA)
To adhere to local regulations, upon reasonable request DNA methylation and clinical outcome data can be shared after the completion of a data transfer agreement (DTA) for non-commercial use. Requests should be made to the corresponding author(s) with responses expected within 14 days of receipt of request.
To identify cell-type distinguishing probes, we obtained purified cell-type specific methylation data from the Gene Expression Omnibus (GEO). Datasets included were GSE110554 (B cells, CD4 and CD8 T cells, monocytes, natural killer), GSE112179 (neurons), GSE122126 (blood endothelium, neurons), GSE140295 (blood endothelium), GSE167998 (B cells, CD4 and CD8 T cells, eosinophils, monocytes, natural killer, neutrophils, T reg), GSE184269 (B cells, CD4 and CD8 T cells, monocytes, natural killer), GSE191200 (microglia), GSE63409 (blood cell progenitors), GSE74877 (endothelium, fibroblasts), GSE82234 (blood endothelium), GSE84395 (blood endothelium), and GSE88824 (B cell, CD4 and CD8 T cells, monocytes, natural killer, neutrophils).

## Research involving human participants, their data, or biological material

Policy information about studies with human participants or human data. See also policy information about sex, gender (identity/presentation), and sexual orientation and race, ethnicity and racism.

| | |
|---|---|
| Reporting on sex and gender | This is not the focus of the current study. We have only listed the sex of the patients included in the figures to show that both sexes are included in the samples analyzed. |
| Reporting on race, ethnicity, or other socially relevant groupings | N/A |
| Population characteristics | The current study has the focus on the tumor micro-enviroment and meningioma classification. Some findings are confirmed in three clinical cohorts for which the clinical characteristics have been decribed extensively before (ref: Maas et. al. Journal of Clinical Oncology 2021). |
| Recruitment | Most cohorts included are of retrospective design and therefore inherit the limitations of such cohorts. One validation cohort is a prospective cohort not including these limitations. |
| Ethics oversight | Tissue collection and processing for RNA sequencing were performed in accordance with local ethics regulations (IRB Heidelberg, S-318/2022). Collection and generation of the meningioma tissue micro-array (TMA) were performed in accordance with local regulations (IRB Tübingen, 618/2014BO2 and 191/2021BO2). The other clinical cohorts included in this manuscript have been described in detail before. In brief, after approval of local authorized regulators, meningioma tissue and patient data were collected from the (archival) databanks of collaborating hospitals in Germany, Austria, Switzerland, United Kingdom and United States. |

Note that full information on the approval of the study protocol must also be provided in the manuscript.

# Field-specific reporting

Please select the one below that is the best fit for your research. If you are not sure, read the appropriate sections before making your selection.

☒ Life sciences ☐ Behavioural & social sciences ☐ Ecological, evolutionary & environmental sciences

For a reference copy of the document with all sections, see nature.com/documents/nr-reporting-summary-flat.pdf

# Life sciences study design

All studies must disclose on these points even when the disclosure is negative.

| | |
|---|---|
| Sample size | No statistical methods were used to determine the samples sizes used in the analyzes. The included clinical cohorts have been used in different previous studies where they were of appropriate size to identify statistical significant patterns that were validated in the validation cohorts. |
| Data exclusions | An earlier batch of snRNA samples with sub-optimal isolation and low reads per cell was excluded after subsequent optimization of protocol yielded substantially higher quality-control values |
| Replication | The experiments included in the manuscript contain numerous replications, almost exclusively, samples from unique patients. Additionally, all core findings in the manuscript are based on multiple types of experiments in different cohorts that contain epigenetic deconvolution, (spatial) RNAseq and protein quantification, thus also including replication in different modes of data presented. |
| Randomization | N/A |
| Blinding | During data collection the investigators were blinded to the outcome of the individual patients. The investigators were not blinded during data analysis. |

# Reporting for specific materials, systems and methods

We require information from authors about some types of materials, experimental systems and methods used in many studies. Here, indicate whether each material, system or method listed is relevant to your study. If you are not sure if a list item applies to your research, read the appropriate section before selecting a response.

## Materials & experimental systems

| n/a | Involved in the study |
|---|---|
| ☐ | ☒ Antibodies |
| ☒ | ☐ Eukaryotic cell lines |
| ☒ | ☐ Palaeontology and archaeology |
| ☒ | ☐ Animals and other organisms |
| ☐ | ☒ Clinical data |
| ☒ | ☐ Dual use research of concern |
| ☒ | ☐ Plants |

## Methods

| n/a | Involved in the study |
|---|---|
| ☒ | ☐ ChIP-seq |
| ☒ | ☐ Flow cytometry |
| ☒ | ☐ MRI-based neuroimaging |

# Antibodies

Antibodies used

anti-CD68 for IHC (Dako M0876, clone PG-M1)
anti-PU.1 (Abcam ab76543, clone EPR3158Y)
anti-HH3 (MSVA MSVA-903M, clone MSVA-903M)
anti-dsDNA (Abcam ab27156, clone 35I9 DANN)
anti-IBA1 (Abcam ab220815, clone EPR16588)
anti-CD68 for MIBI (CST D4B9C, clone 26042SF)
anti-TMEM119 (CST 83308SF, clone E3E4T)
anti-P2RY12 (Abcam ab300141, clone EPR26298-93)

Validation

All antibodies were selected based on multiple lines of evidence, including knockout (KO) validation, published references, cell-type specificity in previous studies, and compatibility with our current MIBI staining protocols. Purchased antibodies underwent rigorous validation on tissue microarrays containing various human tissues (brain, meninges, meningioma, tonsil, colon, and colorectal cancer) to evaluate antibody performance and determine optimal titers. Conjugated antibodies were then tested in MIBI and IHC to confirm successful conjugation and reconstitution. Throughout, we adhered to recommendations from the International Working Group for Antibody Validation (IWGAV), focusing on specificity and reproducibility.
The anti-CD68 and anti-PU.1 stainings for immunohistochemistry were performed in (ISO) accredited clinical pathology laboratories, where each antibody lot is validated using negative and positive controls.

# Clinical data

Policy information about clinical studies
All manuscripts should comply with the ICMJE guidelines for publication of clinical research and a completed CONSORT checklist must be included with all submissions.

Clinical trial registration

N/A

Study protocol

N/A

Data collection

The study was not a clinical trial, but includes clinical data from retrospective analyses to correlate findings with outcome. Data collection for all samples was done by their respective local centers. Retrospective data was obtained from archives of the respective centers as mentioned in the methods section.

Outcomes

The primary outcome measurement is progression-free survival, as determined by the clinicians treating each patient.

# Plants

Seed stocks

N/A

Novel plant genotypes

N/A

Authentication

N/A
